# Integrative In Silico and FFPE Tissue Analyses Elucidate Upregulated Genes in Colorectal Cancer Enriched for Tie2-Expressing Macrophages/Monocytes

**DOI:** 10.3390/ijms27083645

**Published:** 2026-04-19

**Authors:** Eman Amin M. Ali, Alaa Muayad Altaie, Reem Sami Alhamidi, Nival Ali, Anania Boghossian, Marwa Almazrouei, Vidya Bijosh Mohan, Riyad Bendardaf, Rawia Mohamed, Iman M. Talaat, Rifat Hamoudi

**Affiliations:** 1 Research Institute for Medical and Health Sciences, University of Sharjah, Sharjah P.O. Box 27272, United Arab Emirates; u21105945@sharjah.ac.ae (E.A.M.A.); alaa.abed@sharjah.ac.ae (A.M.A.); ralhamidi@sharjah.ac.ae (R.S.A.); nevalalmaleh@gmail.com (N.A.); aboghossian@sharjah.ac.ae (A.B.); marwa.almazrouei@sharjah.ac.ae (M.A.); vmohan@sharjah.ac.ae (V.B.M.); riyad.bendardf@uhs.ae (R.B.); 2 Center of Excellence for Precision Medicine, Research Institute for Medical and Health Sciences, University of Sharjah, Sharjah P.O. Box 27272, United Arab Emirates; 3 Clinical Sciences Department, College of Medicine, University of Sharjah, Sharjah P.O. Box 27272, United Arab Emirates; 4 Oncology Department, University Hospital Sharjah, Sharjah P.O. Box 72772, United Arab Emirates; 5 Center of Excellence for Cancer Research, Research Institute for Medical and Health Sciences, University of Sharjah, Sharjah P.O. Box 27272, United Arab Emirates; 6 Anatomical Pathology Department, Burjeel Medical City, Abu Dhabi P.O. Box 9054, United Arab Emirates; rawia.mohamed@burjeelmedicalcity.com; 7 Pathology Department, Faculty of Medicine, Alexandria University, Alexandria 21131, Egypt; 8 Division of Surgery and Interventional Science, University College London, London NW3 2PS, UK; 9 BIMAI-Lab—Biomedically Informed Artificial Intelligence Laboratory, University of Sharjah, Sharjah P.O. Box 27272, United Arab Emirates; 10 ASPIRE Precision Medicine Research Institute Abu Dhabi, University of Sharjah, Sharjah P.O. Box 27272, United Arab Emirates

**Keywords:** colorectal cancer (CRC), Tie2-expressing macrophages/monocytes (TEMs), microvessel density (MVD), gene set enrichment analysis (GSEA), metastasis, progression, survival analysis

## Abstract

Tumor-associated Tie2-expressing monocytes/macrophages (TEMs) have been implicated in promoting angiogenesis and metastasis in colorectal cancer (CRC), yet the molecular mechanisms linking TEMs infiltration to tumor metastasis and progression remain incompletely defined. This study investigated the distribution of TEMs in CRC and their association with gene expression profiles, microvessel density (MVD), and clinical outcomes. Immunohistochemistry on 30 formalin-fixed paraffin-embedded (FFPE) primary CRC samples revealed that TEMs, which characteristically express tyrosine kinase with immunoglobulin and epidermal growth factor homology domains 2 (Tie2) receptor and CD14, preferentially localize to perivascular regions and are associated with higher histological grade, tumor size, lymph node metastasis, and increased MVD. However, Tie2^−^/CD14^+^ macrophages and CD68^+^ tumor-associated macrophages (TAMs) showed uniform stromal distribution. Gene set enrichment analysis (GSEA) of in silico transcriptomic datasets of metastatic CRC (mCRC) identified enrichment of pathways related to cell–cell recognition, calcium signaling, transcription regulation, and metalloexopeptidase activity in Tie2^+^/CD14^+^ tumors. Subsequent qRT-PCR validation on FFPE primary CRC samples confirmed significant upregulation of C-C chemokine receptor 7 (CCR7), platelet-derived growth factor A (PDGFRA), CBP/p300-interacting transactivator with glutamic acid/aspartic acid-rich carboxyl-terminal domain 2 (CITED2), and carboxypeptidase E (CPE) in TEMs^+^ regions. Notably, angiopoietin1 (Ang1), but not angiopoietin2 (Ang2), was significantly elevated in TEMs^+^ primary tumors. Kaplan–Meier analysis on 1336 CRC patients indicated that high expression of CITED2, CPE, and Ang2 is associated with reduced overall survival. Collectively, these findings suggest that TEM infiltration is linked to transcriptional regulation, biological processes, and enzymatic programs in CRC, potentially contributing to tumor progression and poor prognosis, and highlight CCR7, PDGFRA, CITED2, CPE, and Ang1 as candidate biomarkers for further mechanistic exploration.

## 1. Introduction

Colorectal cancer (CRC), comprising malignancies of the colon, rectosigmoid junction, and rectum, remains a significant global health challenge. In 2022, an estimated 1,926,425 new CRC cases were diagnosed globally, accompanied by roughly 904,000 deaths, accounting for around 9.3% of cancer mortality worldwide [1]. While high-income countries have begun to observe declines in mortality largely attributed to effective screening and earlier detection, many low- and middle-income regions are witnessing rising burdens, particularly among younger populations [2]. Projections suggest that by 2040, yearly new cases may rise to 3.2 million, with deaths approaching 1.6 million, underscoring the pressing need for increasing the implementation of prevention, screening, and treatment strategies [3].

CRC develops through the interplay of modifiable and non-modifiable risk factors. Non-modifiable determinants include older age, male sex, a family history of CRC or advanced adenomas, and inherited syndromes such as Lynch syndrome and familial adenomatous polyposis [4]. Modifiable risk factors with strong epidemiologic evidence include obesity, metabolic syndrome (including hypertension and non-alcoholic fatty liver disease), physical inactivity, diets high in red and processed meats and low in fiber and certain micronutrients (e.g., vitamin D), alcohol consumption, and tobacco use [4,5]. Inflammatory bowel disease, namely ulcerative colitis and Crohn’s disease, also significantly increases CRC risk. Emerging evidence suggests that metabolic biomarkers, such as elevated triglycerides and sedentary behavior, further compound risk, especially in early-onset CRC (diagnosed before age 50) [6].

Recent advances in transcriptomic and multi-omics profiling have delineated several molecular subtypes of CRC that differ markedly in biology and clinical outcome. One widely adopted schema is the Consensus Molecular Subtypes (CMS) classification, which stratifies CRC into four subtypes: CMS1 (MSI (microsatellite instability-immune), CMS2 (canonical), CMS3 (metabolic), and CMS4 (mesenchymal). CMS1 tumors are hypermutated, microsatellite unstable, often harbor BRAF^V600E mutations, and are enriched for immune infiltrates, but paradoxically have poor survival rates; CMS2 tumors show elevated chromosomal instability (CIN), Wingless-related integration site (WNT) and myelocytomatosis oncogene (MYC) pathway activation, and tend to have better prognosis. CMS3 is marked by metabolic dysregulation and mixed genetic features, while CMS4 exhibits strong mesenchymal signatures, transforming growth factor-β (TGF-β) activation, stromal infiltration, and has the worst overall prognosis [7]. Recent work using multi-omics clustering (including gene expression, methylation, and mutation profiles) has identified two major subtypes (CS1 vs. CS2), with CS1 associated with poorer survival, higher hallmark pathway activity, greater genomic alterations, and a less favorable tumor microenvironment than CS2 [8]. Moreover, studies of KRAS-mutant CRC have revealed molecular distinctions (KM1 vs. KM2), with KM2 aligning with features of CMS4, including TGF-β and epithelial–mesenchymal transition (EMT) activation and a worse prognosis, whereas KM1 shows more proliferative and cell-cycle activity associated with better outcomes [9].

The tumor microenvironment (TME) refers to the complex and dynamic milieu surrounding cancer cells, comprising non-malignant stromal components (fibroblasts, endothelial cells, pericytes), extracellular matrix, immune cell infiltrates (e.g., T lymphocytes, macrophages, myeloid-derived suppressor cells (MDSCs)), soluble factors (cytokines, chemokines, growth factors), and biophysical conditions such as hypoxia, nutrient depletion, and interstitial pressure. The interaction among these components influences tumor initiation, progression, metastatic potential, and therapy resistance [10]. In CRC, high infiltration of cytotoxic (CD8^+^) and memory (CD45RO^+^) T cells is strongly associated with reduced recurrence and improved overall survival, even independently of traditional TNM staging. Conversely, an immunosuppressive TME, characterized by regulatory T cells, tumor-associated macrophages (TAMs) (often of the M2 phenotype), dense fibroblast-rich stroma, hypoxia, and metabolic reprogramming, is associated with a worse prognosis and diminished response to chemotherapy, targeted therapy, radiotherapy, and immunotherapy [11]. Moreover, emerging prognostic signatures based on TME-related gene expression successfully stratify CRC patients by risk and predict likely responsiveness to immunomodulatory or cytotoxic regimens [10].

A unique constituent of the TME is a subset of TAMs, known as Tie2 (tyrosine kinase with immunoglobulin and epidermal growth factor homology domains 2)-expressing macrophages (TEMs), which represent a distinct, perivascular population of macrophages that engage the angiopoietin-Tie2 (Ang/Tie2) signaling axis, and growing evidence implicates them in CRC progression, angiogenesis, metastasis, and resistance to therapy [12]. In CRC tumors, elevated levels of angiopoietin 2 (Ang2) and Tie2 are correlated with higher tumor microvessel density (MVD), more advanced stage, increased metastatic potential, and worse overall survival [13,14]. TEMs are thought to respond to hypoxic cues in the TME, localize near blood vessels, and destabilize vascular structures by secreting pro-angiogenic factors such as vascular endothelial growth factor A (VEGFA) and matrix metalloprotease-9 (MMP-9), which can facilitate tumor cell intravasation [15]. Typically, Tie2 receptor activation on myeloid cells is primarily driven by Ang2 in the TME. This signaling enhances pro-angiogenic functions by upregulating thymidine phosphorylase, cathepsin B, MMP-9, and VEGFA, thereby promoting vessel remodeling, sprouting, and tumor angiogenesis via paracrine mechanisms [16,17]. Concurrently, it augments immunosuppressive activity through increased arginase-1, IL-10, and other mediators [18]. These effects reinforce an M2-like, tissue-remodeling phenotype with reduced proinflammatory gene expression [19]. Thus, Ang2/Tie2 activation links angiogenesis and immune evasion, driving tumor progression.

Preclinical models show that CRC metastasis to the liver is worsened when the Ang/Tie2-PI3K/AKT signaling pathway is upregulated in macrophages, such as via loss of SHP-2 (a negative regulator), which suggests that TEMs function contributes to metastatic dissemination and may dampen the efficacy of anti-angiogenic therapies [20]. However, controversy persists: some recent studies question whether Tie2 expression in macrophages is functionally required for tumor angiogenesis, relapse, or metastasis in all settings [21], suggesting that TEMs may vary in significance depending on the tumor model and context. On the other hand, a considerable number of studies provide evidence that targeting TEMs specifically, or components of the Ang/Tie2 axis in general, may hold promise as adjuncts to conventional chemotherapy and anti-angiogenic therapy in CRC [12]. Therefore, more definitive studies are needed to explore TEMs’ biology and validate their role in CRC, which may uncover valuable related biomarkers or therapeutic targets.

While TEMs have been identified as a specialized, perivascular macrophage subset that promotes tumor angiogenesis and progression in various cancers, including CRC, through non-redundant pro-angiogenic functions [17,22], their relationship to MDSCs remains incompletely resolved. Notably, certain monocytic MDSCs (M-MDSCs) populations express Tie2 and display dual immunosuppressive and pro-angiogenic activities, contributing to T-cell inhibition and tumor vascularization. For instance, in melanoma, advanced-stage patients exhibit elevated circulating Tie2^+^ M-MDSCs, where Ang2 activates Tie2 signaling to enhance arginase-1 expression and suppress melanoma-specific T-cell responses, thereby fostering an immunosuppressive TME [18]. Similar mechanisms may apply in CRC, as myeloid cells with overlapping Tie2 expression can theoretically bridge angiogenesis and immune evasion across solid tumors. Addressing this distinction through multi-marker phenotyping (e.g., combining Tie2 with MDSC-defining markers such as CD14^+^, HLA-DR^−^/low) [23] and functional assays is essential to clarify whether the observed TEMs represent a distinct subpopulation or a subset of immunosuppressive M-MDSCs, which could refine therapeutic targeting strategies.

In this study, we compared metastatic CRC (mCRC) samples with high levels of TEMs (Tie2^+^/CD14^+^) to those with low levels of TEMs (Tie2^−^/CD14^+^) by analyzing their molecular differences using gene set enrichment analysis (GSEA) on publicly available datasets. We then confirmed these findings using RT-qPCR on primary FFPE CRC samples that were classified as TEMs low or high based on Tie2 and CD14 double staining. We also used immunohistochemistry (IHC) to examine where TEMs are localized in the primary CRC tissues and how many are present, and to see how they relate to tumor stage, grade, and MVD. Finally, we evaluated the prognostic value of the selected genes through survival analysis using the Kaplan–Meier Plotter platform.

## 2. Results

### 2.1. Immunohistochemical Assessment of TEM and TAM Distribution in Primary CRC

**Figure 1:**
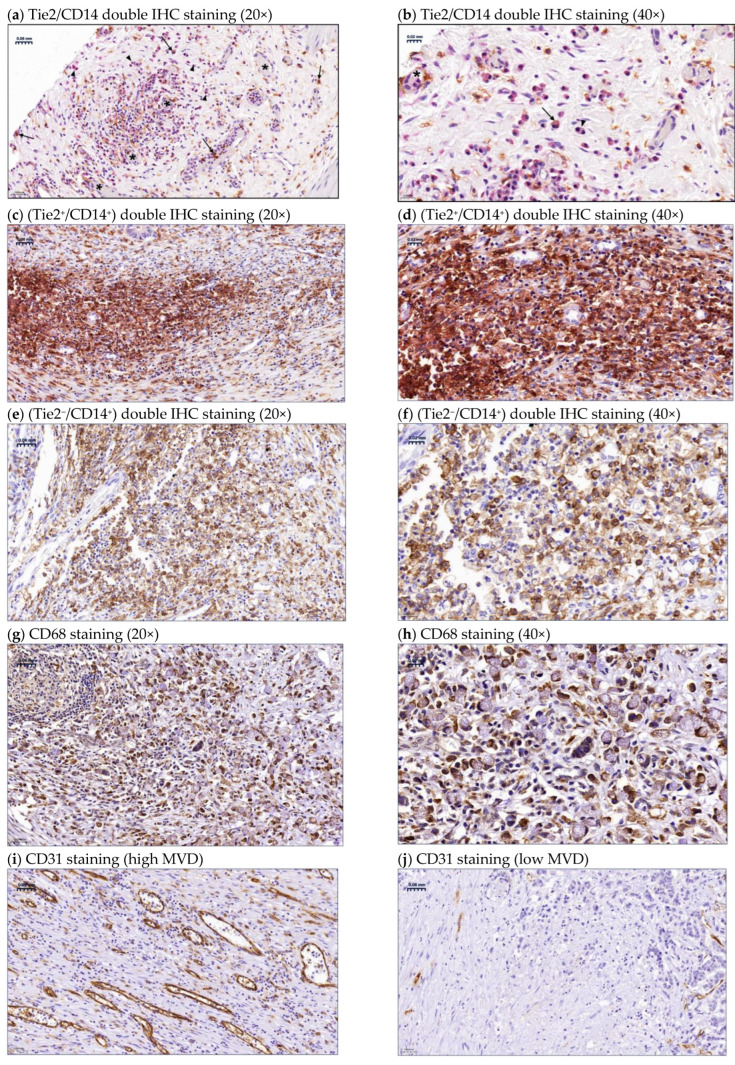
Immunohistochemical detection of tyrosine kinase with immunoglobulin and epidermal growth factor homology domains 2 (Tie2)-expressing macrophages (TEMs): Tie2^+^ (pink)/CD14^+^ (brown), and Tie2^−^/CD14^+^ macrophage subset. (**a**) (20×) and (**b**) (40×) showing their localization at areas of neovascularization, as Tie2 stains endothelial cells as well (pink); arrows indicate Tie2^+^/CD14^+^ TEMs, arrowheads indicate Tie2^+^/CD14^−^ TEMs (Tie2-expressing mononuclear cells, morphologically consistent with macrophages), and asterisks indicate microvessels. (**c**) (20×) and (**d**) (40×) show an area of high density of Tie2^+^/CD14^+^ TEMs. (**e**) (20×) and (**f**) (40×) show the uniform distribution of Tie2^−^/CD14^+^ macrophages in the tumor stroma. (**g**) (20×) and (**h**) (40×) show the uniform distribution of CD68^+^ TAMs. Microvessel density (MVD) assessment using CD31 (endothelial cell marker): (**i**) (high MVD), (**j**) (low MVD). Left side: 20× magnification (scale bar 0.06 mm); right side: 40× magnification (scale bar 0.02 mm).

Typical images for the tissue distribution of TEMs (Tie2^+^/CD14^+^), (Tie2^−^/CD14^+^) macrophages, and (CD68^+^) TAMs are shown in Figure 1a–h; the respective statistical evaluation of all patients is summarized in Table 1 and Table 2. TEMs are preferentially located in areas of tumor neovascularization and double-stained for Tie2^+^ (pink)/CD14^+^ (brown), with some Tie2^+^ mononuclear cells (pink) that are morphologically consistent with macrophages but negative for CD14 being counted as TEMs [24], and they showed a preference for perivascular regions, similar to the Tie2^+^/CD14^+^ category (Figure 1a,b). Tie2 is also expressed by endothelial cells [25]; therefore, Tie2 staining can delineate blood vessels, as shown in Figure 1a,b. On the other hand, Tie2^−^/CD14^+^ macrophages (Figure 1e,f) and CD68^+^ TAMs (Figure 1g,h) showed uniform density throughout the tumor section. Figure 1i,j show MVD using CD31 as an endothelial marker. Of note, areas of high MVD (Figure 1i) coincided with TEM-rich foci, while areas of low MVD (Figure 1j) coincided with TEM-low regions in most samples.

#### TEMs Associate with Histological Grade, Tumor Size, Lymph Node Metastasis and Tumor Neovascularization in CRC

In 30 FFPE CRC samples, TEM infiltration is significantly associated with histological grade (*p* = 0.008), with 77.78% of TEMs^+^ tumors being moderately/poorly differentiated, while 22.22% of TEMs^+^ samples are well differentiated. TEMs infiltration also shows a significant association with tumor size, as 94.12% of TEMs^+^ samples have an advanced tumor stage (T3/T4) according to the TNM staging system, while only 5.88% of TEMs patients have a stage of T3/T4 (*p* < 0.0001). Moreover, the presence of tumor spread to lymph nodes is significantly higher in TEMs^+^ cases 94.12%, compared to 5.88% in TEMs^−^ counterparts (*p* < 0.0001). Additionally, tumor MVD showed significantly higher scores in TEMs^+^ tumors (*p* = 0.0012), with 88.89% of TEMs^+^ tumors showing high MVD, and 11.11% showing low MVD (Table 2). However, distant metastasis showed a trend to be higher in TEMs^+^ patients (*p* = 0.0568), while patient’s gender and age were not significantly associated with TEMs infiltration. Nevertheless, total TAM counts did not show a significant variation between TEMs^+^ and TEMs^−^ patients, as revealed by CD68 and CD14 single staining (Table 2).

### 2.2. Gene-Set Enrichment Analysis of Differentially Expressed Genes on In Silico Data of mCRC Highlights Cell–Cell Recognition, Calcium-Mediated Signaling, Transcription Regulation, and Enzymatic Activity as Distinct Molecular Signatures Between Tie2^+^/CD14^+^ and Tie2^−^/CD14^+^ mCRC

Absolute (absGSEA) and Standard GSEA were conducted as described in [26] to identify significantly enriched pathways in Tie2^+^/CD14^+^ vs. Tie2^−^/CD14^+^ mCRC groups. Transcriptomic data has been obtained from the Gene Expression Omnibus “https://www.ncbi.nlm.nih.gov/geo (accessed on 15 January 2025)”. Accession number: GSE41568. Utilizing the MSigDB database that encompasses diverse gene sets from the C1 to C8 collections, and using a nominal *p*-value threshold of <0.05 and a False Discovery Rate (FDR) of q-value < 0.25, a total of 6, 182, 317, 54, 476, 36, 624, and 43 significant pathways were identified from C1, C2, C3, C4, C5, C6, C7, and C8, respectively. The enriched pathways demonstrate diverse biological processes and molecular functions involving cell–cell recognition, calcium-mediated signaling, transcription regulation, and enzymatic activity. Gene sets used (C1–C8) from the MSigDB database. Their descriptions are shown in Table S1 in the Supplementary Materials, and the full list of significant pathways from each collection is provided in Tables S2–S9. After identifying the significant pathways, gene frequency within each set was analyzed to assess their level of association and relevance to Tie2^+^/CD14^+^ vs. Tie2^−^/CD14^+^ groups of mCRC samples. Table S10 presents the top 20 frequent genes that belonged to C3, C4, and C5, selected as most relevant to cancer biology, and ranked from highest to lowest based on their frequency, all with (*p* < 0.05). The log2 fold change values are oriented towards the Tie2^+^/CD14^+^ group, indicating differences in expression in this condition. Genes with higher frequency are likely to play critical roles in pathways enriched for Tie2^+^/CD14^+^ mCRC, explaining the association of those two markers (Tie2 and CD14) and possibly TEMs’ role in CRC metastasis. Our analysis centered on C3 and C5 to better characterize the regulatory genes and the biological processes or molecular functions that TEMs infiltration may influence in mCRC.

**Figure 2:**
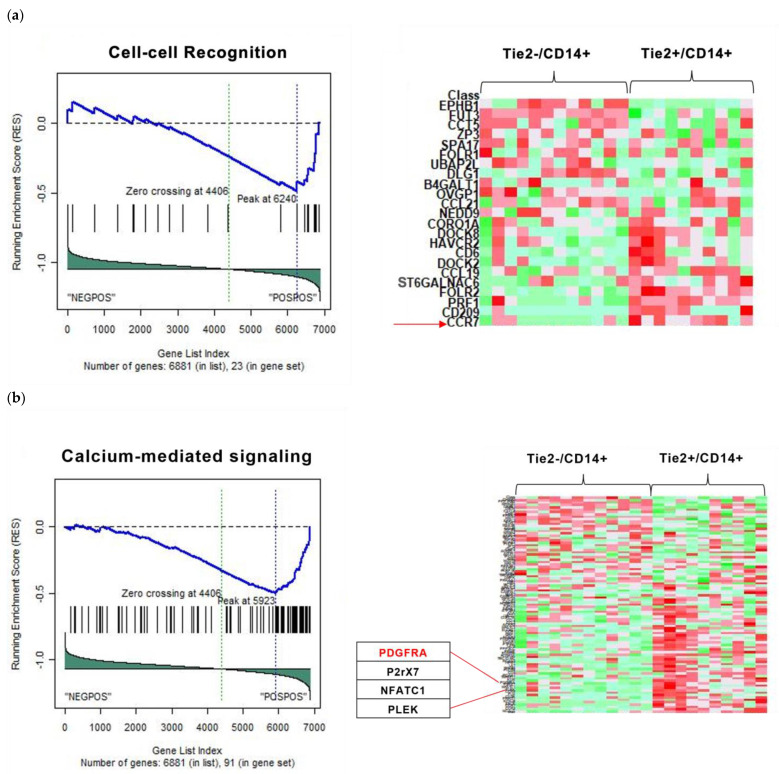

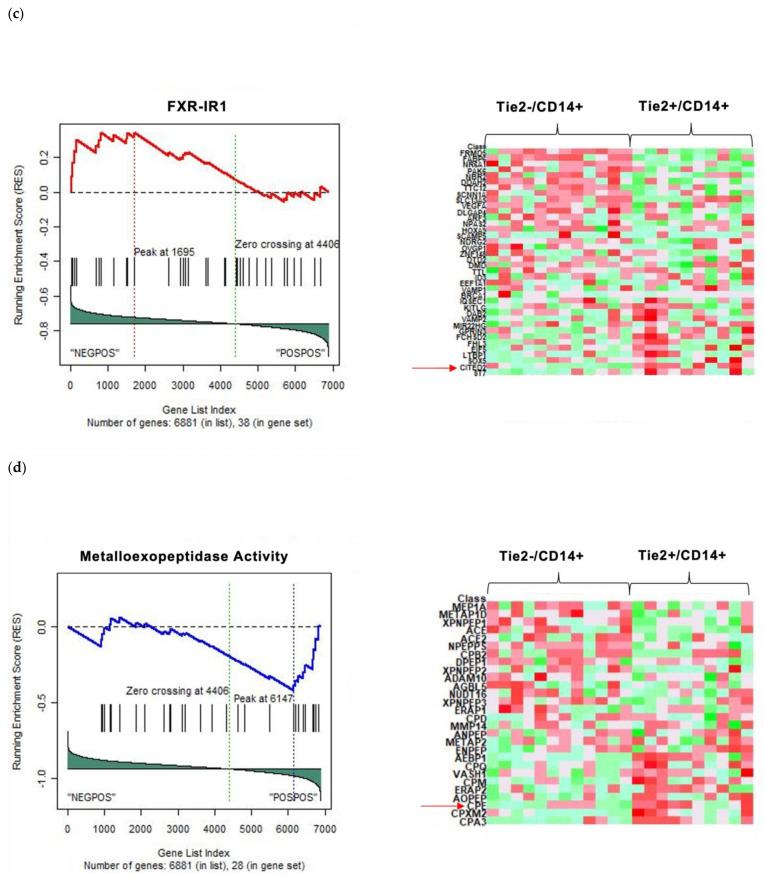
Gene set enrichment analysis (GSEA) output display of the selected pathways. **Left side**: enrichment plots obtained from data analysis of Tie2^+^/CD14^+^ vs. Tie2^−^/CD14^+^ mCRC samples. The blue line represents the running enrichment score, and black vertical ticks mark the positions of genes in the gene set. **Right side**: corresponding heatmaps display the ranked gene list with red and its shades indicate gene upregulation, and green and its shades indicate gene downregulation: (**a**) Cell–cell recognition pathway includes significantly upregulated genes in Tie2^+^/CD14^+^ group, such as C-C chemokine receptor 7 (CCR7) (*p* = 0.00085), CD209 (*p* = 0.021939), folate receptor 2 (FOLR2) (*p* = 0.000729), and perforin1(PRF1) (*p* = 0.004596). CCR7 (pointed to by the red arrow) was selected for downstream validation based on its frequency. (**b**) The calcium-mediated signaling pathway was found to be differentially expressed, with significantly upregulated genes in the Tie2^+^/CD14^+^ group, including platelet-derived growth factor A (PDGFRA) (*p* = 6.64 × 10^−5^), purinergic receptor P2X 7 (P2RX7) (*p* = 0.02205), nuclear factor of activated T-cells 1 (NFATC1) (*p* = 0.023506), and pleckstrin (PLEK) (*p* = 0.036616). PDGFRA (in red) was selected for downstream validation based on frequency. (**c**) The transcription regulation pathway FXR response element (FXRE) of the IR1 type (inverted repeat separated by one nucleotide) (FXR-IR1), with suppression of tumorigenicity 7 (ST7) (*p* = 0.041322) and CBP/p300-interacting transactivator with glutamic acid/aspartic acid-rich carboxyl-terminal domain 2 (CITED2) (*p* = 0.001282) are upregulated in the Tie2^+^/CD14^+^ group. CITED2 (pointed to with red arrow) was chosen for downstream validation based on frequency. (**d**) The metalloexopeptidase gene set was significantly enriched, with members such as aminopeptidase O (putative) (AOPEP) (*p* = 0.039798), carboxypeptidase E (CPE) (*p* = 0.027692), carboxypeptidase X, M14 family member 2 (CPXM2) (*p* = 0.042482), and carboxypeptidase A3 (CPA3) (*p* = 0.006433) upregulated in the Tie2^+^/CD14^+^ group. CPE (indicated by the red arrow) was selected for downstream validation based on frequency.

The cell–cell recognition pathway was identified in C5, with DEGs in the Tie2^+^/CD14^+^ cohort compared to the Tie2^−^/CD14^+^ control group. Examples of genes upregulated in Tie2^+^/CD14^+^ samples are C-C chemokine receptor 7 (CCR7) (*p* = 0.00085), CD209 (*p* = 0.021939), folate receptor 2 (FOLR2) (*p* = 0.000729), and perforin1 (PRF1) (*p* = 0.004596) (Figure 2a). CCR7 was selected for downstream validation, as it is the most frequently occurring gene within the top 20 gene list. CCR7 plays an important role in the mechanism of lymph node spread in CRC [27] and has been highlighted as a significant independent predictor of reduced overall survival in CRC patients [28]. Consequently, it is crucial to investigate the mechanisms underlying its upregulation.

Furthermore, the calcium-mediated signaling pathway in C5 was found to be differentially expressed, with platelet-derived growth factor A (PDGFRA) (*p* = 6.64 × 10^−5^), purinergic receptor P2X 7 (P2RX7) (*p* = 0.02205), nuclear factor of activated T-cells 1 (NFATC1) (*p* = 0.023506), and pleckstrin (PLEK) (*p* = 0.036616) showing significant upregulation in the Tie2^+^/CD14^+^ group (Figure 2b). PDGFRA was selected for downstream validation based on its frequency.

Additionally, the transcription regulation pathway FXR-IR1 in C3, which refers to an FXR response element (FXRE) of the IR1 type (inverted repeat separated by one nucleotide), a specific DNA sequence motif found in the promoter region of target genes regulated by the farnesoid X receptor (FXR) protein [29], was detected to be differentially expressed, with members like suppression of tumorigenicity 7 (ST7) (*p* = 0.041322) and CBP/p300-interacting transactivator with glutamic acid/aspartic acid-rich carboxyl-terminal domain 2 (CITED2) (*p* = 0.001282) being upregulated in the Tie2^+^/CD14^+^ group (Figure 2c). CITED2 was selected for qPCR validation based on frequency.

Furthermore, the metalloexopeptidase gene set was significantly enriched in C5, with members such as aminopeptidase O (putative) (AOPEP) (*p* = 0.039798), carboxypeptidase E (CPE) (*p* = 0.027692), carboxypeptidase X, M14 family member 2 (CPXM2) (*p* = 0.042482), and carboxypeptidase A3 (CPA3) (*p* = 0.006433) upregulated in the Tie2^+^/CD14^+^ group (Figure 2d). CPE was selected for downstream validation based on its frequency. Metallocarboxypeptidases (i.e., metalloexopeptidases) have been increasingly implicated in CRC. Several studies have demonstrated that CPE expression is markedly upregulated in CRC cell lines and patient tumor samples compared to normal colorectal epithelium [30], further underscoring the necessity to investigate its biological significance in CRC.

**Figure 3:**
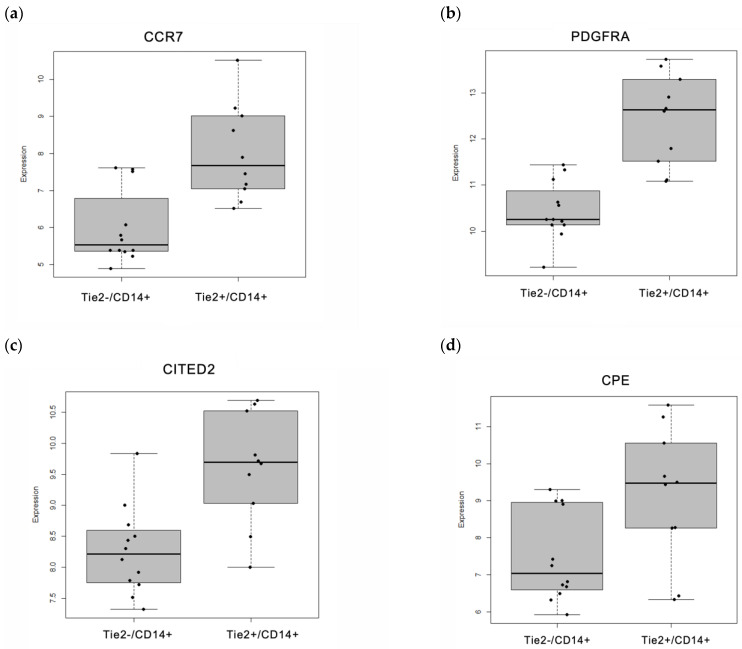

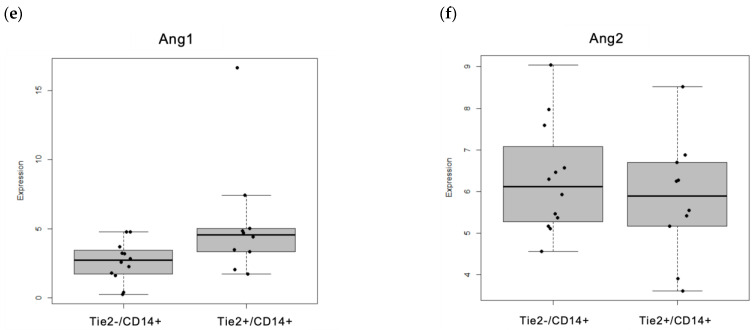
Differentially expressed genes (DEGs) selected for validation are presented as boxplots generated using R version 4.3.2, revealing distinct differential expression profiles between Tie2^+^/CD14^+^ and Tie2^−^/CD14^+^ groups. (**a**) CCR7 (*p* = 0.00085), (**b**) PDGFRA (*p* = 6.64 × 10^−5^), (**c**) CITED2 (*p* = 0.001282), (**d**) CPE (*p* = 0.027692), (**e**) Ang1 (*p* = 0.0798), and (**f**) Ang2 (*p* = 0.1197). *p*-values were calculated using the F-test. These boxplots illustrate differential expression patterns identified through analysis of raw transcriptomic data.

Boxplot graphs in Figure 3a–d present the significant gene expression differences of the selected biomarkers in the Tie2^+^/CD14^+^ group compared to the Tie2^−^/CD14^+^ controls.

Conversely, the two canonical Tie2 ligands, angiopoietin 1 (Ang1) and Ang2, did not exhibit significant differences between the Tie2^−^/CD14^+^ and Tie2^+^/CD14^+^ groups (Figure 3e,f). Notably, Ang2, which is widely recognized for its role in TEMs recruitment [31], showed no statistical significance (*p* = 0.1197), while Ang1, a constitutive Tie2 agonist capable of engaging Tie2 on monocytes/macrophages and promoting a shift toward a more inflammatory phenotype [32], displayed a trend toward higher expression in the Tie2^+^/CD14^+^ group (*p* = 0.0798). These findings suggest that Ang2 may not be the primary chemoattractant for TEMs in this context, pointing to Ang1 as a key regulator of TEMs’ function. As Ang1 and Ang2 were not identified among the significantly enriched pathways, heatmaps were not generated for these genes. Instead, their raw expression levels were examined directly, and Figure 3e,f present boxplots displaying Ang1 and Ang2 expression, respectively, in Tie2^+^/CD14^+^ samples compared with Tie2^−^/CD14^+^ controls.

### 2.3. Experimental Validation of In Silico Identified Candidate Genes Through RT-qPCR Analysis

**Figure 4:**
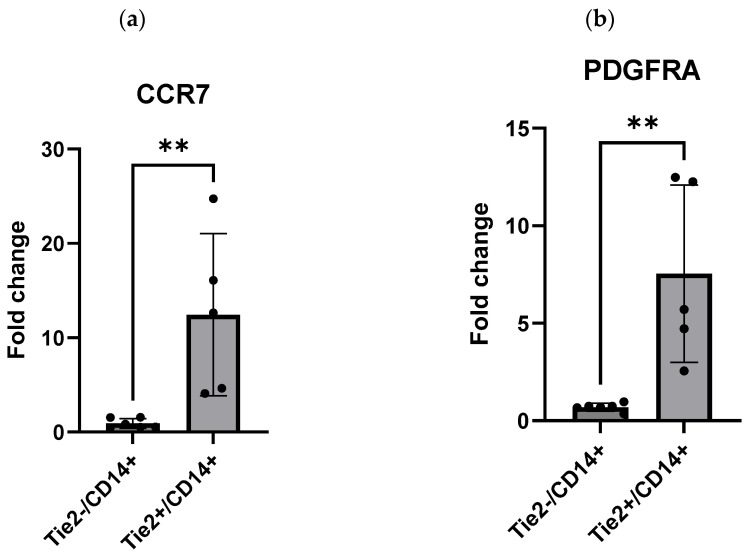

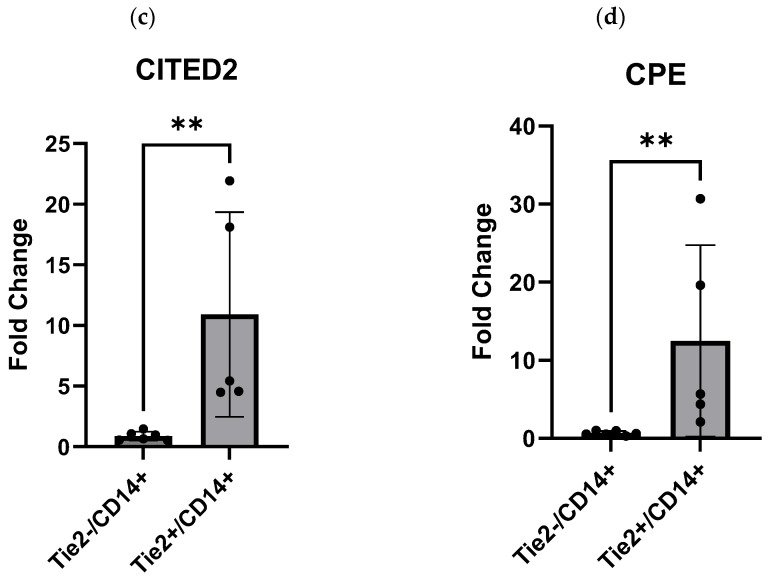
Quantitative real-time PCR of selected genes identified in enriched pathways. Upregulation of (**a**) CCR7 in Tie2^+^/CD14^+^ specimens compared to Tie2^−^/CD14^+^ (as a control) (*p* = 0.0043), (**b**) PDGFRA in Tie2^+^/CD14^+^ specimens compared to Tie2^−^/CD14^+^ (as a control) (*p* = 0.0043), and (**c**) CITED2 in Tie2^+^/CD14^+^ samples compared to Tie2^−^/CD14^+^ controls (*p* = 0.0043). (**d**) CPE in Tie2^+^/CD14^+^ samples compared to Tie2^−^/CD14^+^ controls (*p* = 0.0043). Each sample was technically replicated three times. The data displays the fold change in gene expression between groups. The data were analyzed using the Mann–Whitney test. A *p* ≤ 0.05 was considered significant; ** represents a *p* < 0.01.

qRT-PCR on macrodissected Tie2^+^/CD14^+^ and Tie2^−^/CD14^+^ regions from FFPE samples of primary CRC aligned with the GSEA findings of mCRC data, showing significant upregulation of CCR7, PDGFRA, CITED2, and CPE in Tie2^+^/CD14^+^ tumors (*p* = 0.0043 for each using the Mann–Whitney test; Figure 4a–d). These results align with the heatmaps and boxplots from the in silico analysis (Figure 2a–d and Figure 3a–d), further supporting an association between TEM infiltration and the elevated expression of these four key genes in CRC metastasis.

#### Angiopoietin1 Shows Upregulation in Tie2^+^/CD14^+^ FFPE Samples

**Figure 5:**
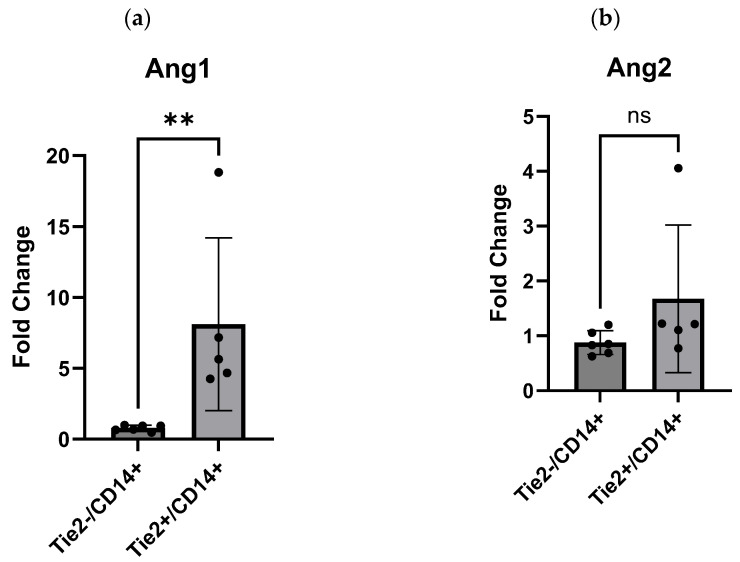
Quantitative real-time PCR of angiopoietin 1 (Ang1) and angiopoietin 2 (Ang2). (**a**) Ang1 (*p* = 0.0043) and (**b**) Ang2 (*p* = 0.0823). Each sample was technically replicated three times. The data displays the fold change in gene expression between groups. The data were analyzed using Mann–Whitney test. A *p* ≤ 0.05 was considered significant; ** represents a *p* < 0.01, ns represents not significant.

qRT-PCR validation of Ang1 and Ang2 in FFPE primary CRC samples demonstrated that Ang1 was significantly upregulated in the Tie2^+^/CD14^+^ group (*p* = 0.0043) (Figure 5a), whereas Ang2 showed only a non-significant trend toward higher expression in the same group (*p* = 0.0823) (Figure 5b). These findings align with the GSEA-derived observations in Section 2.2. suggesting that Ang1 may play a more prominent role in TEMs biology and CRC metastasis. In contrast, despite its established involvement in TEMs recruitment and function [31], Ang2 may not represent the primary chemoattractant driving TEMs infiltration in CRC.

### 2.4. Kaplan–Meier Survival Analysis Shows Reduced Survival in Patients with High Expression of the Selected Candidate Genes (CCR7-PDGFRA-CITED2-CPE), Ang1, and Ang2, with Varying Levels of Significance

**Figure 6:**
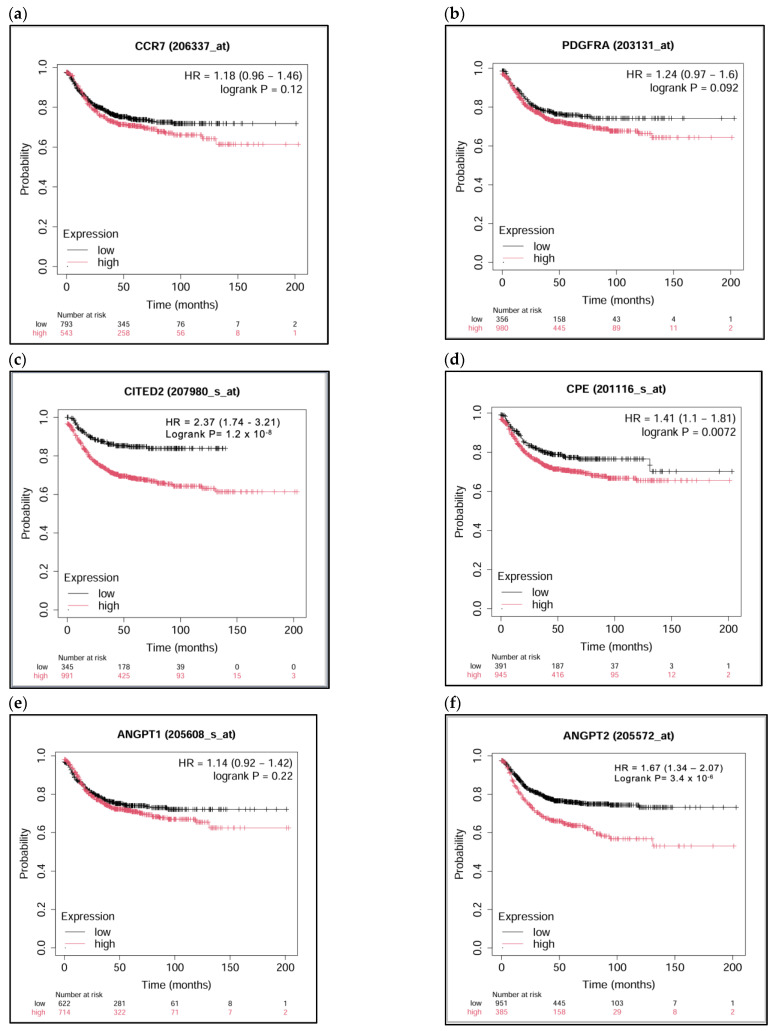
Kaplan–Meier survival analysis of the selected candidate genes, along with angiopoietin1 (Ang1) and angiopoietin2 (Ang2), using KM Plotter. Panels show: (**a**) CCR7, (**b**) PDGFRA, (**c**) CITED2, (**d**) CPE, (**e**) Ang1, and (**f**) Ang2. *p*-values are indicated within each plot; HR: hazard ratio.

Survival analysis using the online KM Plotter platform [33] demonstrated that elevated expression of CITED2 (*p* = 1.2 × 10^−8^) (Figure 6c), CPE (*p* = 0.0072) (Figure 6d), and Ang2 (*p* = 3.4 × 10^−6^) (Figure 6f) was significantly associated with poorer overall survival in CRC patients. PDGFRA exhibited a trend toward reduced survival with higher expression (*p* = 0.092) (Figure 6b), whereas CCR7 (*p* = 0.12) (Figure 6a) and Ang1 (*p* = 0.22) (Figure 6e) did not show statistically significant associations. Nevertheless, Kaplan–Meier curves for CCR7 and Ang1 suggested a tendency toward decreased survival over time with increased expression.

These findings suggest that CITED2, CPE, and Ang2 may serve as valuable prognostic markers in CRC, given their strong association with reduced patient survival. PDGFRA, CCR7, and Ang1, while not reaching statistical significance, may still hold predictive value in specific contexts or in combination with other markers.

## 3. Discussion

Based on current evidence, this study is the first to investigate differential gene expression profiles between TEMs-high and TEMs-low mCRC using GSEA and correlating the signature of mCRC to an independent primary CRC cohort of a diverse population residing in the UAE using IHC and RT-qPCR in the context of TEMs infiltration. Our findings offer important insights into potential TEM-associated molecular mechanisms driving CRC progression, unveiling distinct transcriptional patterns and enriched pathways that may underlie disease advancement and the poorer prognosis associated with elevated TEM infiltration.

Our results align with the emerging recognition of TEMs as a distinct pro-angiogenic macrophage subset contributing to tumor vascularization and aggressive tumor behavior in CRC. Our recent review focusing on the role of TEMs in CRC highlights that TEMs localize in perivascular tumor areas, respond to Ang/Tie2 signaling and contribute to vessel destabilization and the formation of permissive niches for cancer cell intravasation, thereby linking TEMs’ presence to increased angiogenesis and metastasis in CRC [12]. IHC results in this study further confirm TEMs’ correlation to higher tumor grade, size, lymph node metastasis, and MVD in CRC, supporting the notion that TEMs infiltration may serve as both a histopathological marker of aggressive CRC and a potential prognostic biomarker.

Furthermore, our study demonstrated through GSEA on in silico mCRC transcriptomic data followed by quantitative real-time PCR validation of selected enriched genes on primary CRC FFPE samples that CCR7, PDGFRA, CITED2, and CPE genes are significantly upregulated in Tie2^+^/CD14^+^ (TEMs^+^) tumors compared with Tie2^−^/CD14^+^ (TEMs^−^) parallels. This association may help elucidate the advanced disease stage linked to TEMs infiltration in CRC, complementing the well-established role of TEMs in promoting angiogenesis and facilitating metastatic progression [12].

CCR7 has emerged as a potentially important mediator in CRC, contributing to tumor progression, treatment resistance, and metastatic behavior. Although its roles are complex and context-dependent, accumulating evidence points toward a protumorigenic function in CRC. Recent studies suggest that CCR7 is overexpressed in CRC tissues compared to normal counterparts, and this overexpression is associated with more aggressive tumor features. For instance, Gao et al. (2019) demonstrated that high CCR7 expression correlates with greater tumor depth and lymph node metastasis, as well as reduced overall survival (OS) and disease-free survival (DFS) in CRC patients [34]. Moreover, mechanistic investigations suggest that CCL21/CCR7 signaling can enhance features like EMT, stem-cell-like traits, and chemotherapy resistance via pathways including PI3K/AKT/GSK-3β in CRC cell models [27]. A broader analysis across solid tumors further supports this link; a meta-analysis found that elevated CCR7 generally predicts worse survival outcomes [35]. Conversely, our survival analysis using the online KM Plotter platform in a cohort of 1336 CRC patients did not reveal a statistically significant difference between high and low CCR7 expression; however, the survival curves still indicate a trend toward reduced survival in patients with high CCR7 levels. Clinically, CCR7 might become a therapeutic target, as structural and pharmacologic studies of CCR7 have revealed a binding pocket at its cytoplasmic side that might be suitable for antagonist binding [36]. Importantly, CCR7’s dual role in immune-cell trafficking (e.g., in dendritic cells) and cancer-cell dissemination necessitates a careful therapeutic balance, as inhibiting CCR7 in tumor cells may block metastasis but may also perturb immune surveillance [37].

PDGFRA is a tyrosine kinase receptor that plays an increasingly recognized role in the biology of CRC. Its overexpression and altered signaling promote key oncogenic processes such as angiogenesis, proliferation, chemotaxis and EMT. For example, high PDGFRA expression is enriched in the aggressive CMS4 molecular subtype of CRC and correlates with younger patient age, metastasis-related gene signatures and increased sensitivity to the multi-kinase inhibitor regorafenib in patient-derived cells [38]. Additionally, our survival analysis results revealed a trend toward lower survival rates in CRC associated with high PDGFRA expression. Moreover, studies demonstrate a significant association between PDGFRA overexpression and RAS-wild-type status, suggesting that PDGFRA signaling may play a particularly prominent role in CRC tumors lacking RAS mutations; therefore, it might be a contributor to resistance to anti-EGFR therapy in this context [39]. Interestingly, a cross-signaling study by Moench et al. (2022) demonstrated that, in CRC cells, PDGFs, including PDGFRA, can engage non-canonical receptors such as VEGFRs and EGFR when classical PDGFR expression is low, thereby sustaining PI3K/AKT/mTOR signaling [40]. Accordingly, combination strategies potentially including VEGFR, EGFR, or downstream pathway inhibitors may be required to overcome pathway redundancy and adaptive signaling. Overall, PDGFRA offers promise both as a prognostic marker and as a targetable molecule in CRC stratification and treatment approaches.

CITED2 is a transcriptional co-factor that modulates numerous cellular processes by interacting with transcription factors and the p300/CBP coactivator complex [41]. Although it has been widely studied in various cancers, its role in CRC is not fully understood. Knockdown of CITED2 has been shown to increase cell invasiveness. Bai et al. (2007) demonstrated that silencing CITED2 in RKO colon cancer cells upregulated the expression of matrix metalloproteinase-13 (MMP-13), a protease linked to cancer cell migration and invasion [42]. This single in vitro study is insufficient to conclude that CITED2 functions as a tumor suppressor in CRC, as broader data indicate opposing effects. For instance, high CITED2 expression has been linked to irinotecan resistance in CRC cell lines, where CITED2 was upregulated in resistant cells, implying a pro-tumorigenic contribution in therapy contexts [43]. Interestingly, using the SW480 cell line demonstrated that silencing of CITED4, which is a member of the CITED protein family (CITED 1-4) and shares some structural and functional similarities with CITED2, modulates the expression of tight junction-related genes and reduces cellular proliferation while exerting no significant effects on apoptosis, colony formation, migration, invasion, or cell adhesion [44]. These findings contrast with the Bai et al. (2007) conclusion and align with observations in other cancers where CITED2 promotes metastasis or hypoxia adaptation [41], though direct studies about the role of the CITED2 gene in CRC remain sparse. CITED2’s dual potential—suppressing invasion in some settings while correlating with drug resistance in others—highlights the need for further in vivo and clinical investigations to clarify its context-specific functions in CRC. Additionally, our survival analysis results showed that significantly lower survival rates are associated with high CITED2 expression levels, supporting that the CITED2 gene might have a stronger pro-tumorigenic than tumor suppressor role.

CPE is a metalloexopeptidase classically known for its role in prohormone and neuropeptide processing. Emerging data implicate CPE in cancer biology, including CRC, where it may function as an oncogenic driver of tumor growth and proliferation [30]. CPE mRNA and protein expression was found to be significantly upregulated in CRC cell lines, primary tumors, and especially metastatic lesions compared to normal colon tissue, with substantial increases observed during early CRC onset and lymph node metastasis [45].

In functional assays, overexpression of full-length CPE promoted proliferation, increased the fraction of cells in S phase, enhanced colony formation, and augmented tumorigenicity in CRC cells. Additionally, knockdown of CPE reduced proliferation and impaired anchorage-independent growth, indicating a growth-promoting role. At the molecular level, CPE appears to regulate the cell cycle by modulating expression of key factors; CPE overexpression suppresses the cyclin-dependent kinase (CDK) inhibitors p21 and p27 while upregulating cyclin D1, thereby facilitating the G1–S transition and increased cell proliferation [30]. Mechanistically, beyond its classical enzymatic role in peptide processing, CPE appears to engage signaling pathways; it can modulate the Wnt/β-catenin pathway and is also known to activate ERK and mTOR signaling [46]. Intriguingly, in other contexts, a truncated splice variant of CPE (CPE-ΔN) localizes to the nucleus and acts as a transcriptional regulator of genes involved in metastasis, suggesting that different isoforms of CPE may contribute to tumor progression via distinct mechanisms [45]. Clinically, elevated CPE (and its splice variants) has been proposed as a prognostic biomarker in several cancers, including CRC, and might represent a promising therapeutic target [30,45]. Our KM Plotter survival analysis of CPE in CRC showed a significantly reduced survival rate, with high CPE expression aligning with its known role in CRC and reinforcing CPE’s potential as a diagnostic/prognostic marker and therapeutic target in CRC.

On the other hand, focusing on Ang1 and Ang2, the two principal ligands that bind and activate the Tie2 receptor [25], our study disclosed a differential pattern of expression across TEMs^+^ and TEMs^−^ CRC samples in Ang1, while Ang2 showed non-significant differences. Our GSEA analysis of mCRC data showed a trend toward higher Ang1 expression in Tie2^+^/CD14^+^ tumors compared to Tie2^−^/CD14^+^ controls, whereas qRT-PCR analysis confirmed a significant upregulation of Ang1 in Tie2^+^/CD14^+^ primary tumors, underscoring a potentially important role for Ang1 in TEM biology in CRC. Ang1 is a paracrine ligand expressed mainly by pericytes and acts as a strong Tie2 receptor agonist that supports endothelial cell survival, vessel stability, and endothelial barrier function [47]. However, in mCRC, especially in the liver, Ang1 seems to promote tumor cell motility and vessel co-option, thereby contributing to therapy resistance and aggressive growth [48]. In contrast, Ang2 expression did not differ significantly between groups based on GSEA results, although qRT-PCR results in primary CRC samples suggested a modest trend toward higher Ang2 levels in Tie2^+^/CD14^+^ samples. These findings imply that Ang2 may not serve as the dominant chemoattractant for TEMs as previously proposed [31], and that alternative chemotactic pathways may play a more prominent role. For instance, the CXCL12/CXCR4 axis has been implicated in TEMs recruitment in experimental N202 mammary tumors, where CXCR4 expression was significantly higher in TEMs than in Tie2^−^ TAMs [49]. Therefore, further investigation into the mechanisms governing TEM recruitment and infiltration in CRC is essentially needed, as this may reveal actionable targets to reduce TEM accumulation and enhance patient responses to anti-angiogenic and immunotherapeutic strategies, particularly given the documented role of TEMs in CRC treatment resistance [12]. In the past, much work has focused on how TEMs are regulated by Ang2 [12], and on their paracrine support of endothelial cell proliferation [50,51]. By contrast, fewer studies focused on Ang1 in the context of TEMs; therefore, direct evidence that TEMs themselves are a major source of Ang1 to explain the higher expression levels in TEMs^+^ CRC revealed by our findings is limited. Nonetheless, several old studies report Ang1 expression in macrophages generally, without specifying a subtype. It was detected in tissue injury and certain culture settings. For example, histological studies of injured brain detected Ang1 in macrophage populations after 2 to 6 days of injury, and the progressive increase in Ang1 mRNA and protein, along with a decrease in Ang2, coincided with cerebrovascular angiogenesis [52]. Moreover, in vitro work demonstrated that primary human macrophages could exhibit Ang1 mRNA and protein depending on culture substrate and activation conditions [53]. These observations indicate that macrophages can produce Ang1 in some physiological and pathological contexts. But large-scale or definitive demonstrations that TEMs specifically produce functional Ang1 in vivo remain scarce. Therefore, we hypothesize that TEMs, under certain conditions, like hypoxia, matrix composition, contact with particular stromal cells, or exposure to cytokines or damage signals, can reprogram their transcriptomes toward vessel maturation and repair programs that include Ang1 upregulation, as in vitro substrate experiments show that macrophage Ang1 expression depends on contact cues [53]. Moreover, TEMs are heterogeneous; as we highlighted previously in (2.1.), TEMs are generally identified as Tie2^+^/CD14^+^, but some show a Tie2^+^/CD14^−^ phenotype. Therefore, a subfraction of TEMs might upregulate Ang1 in specific niches while others do not. Single-cell resolution studies are required to resolve this issue. Should TEMs indeed produce Ang1, this could have two key implications; firstly, an autocrine feedback loop in which Ang1–Tie2 interaction modulates macrophage phenotype and inflammatory responsiveness [32], and secondly, a paracrine effect on endothelial cells to enhance vascular stabilization and pericyte recruitment [54], which can theoretically aid in forming more competent blood vessels that further nourish tumors and resist anti-angiogenic therapy. In summary, the unexpected upregulation of Ang1 (but not Ang2) in TEMs-high primary colorectal tumors is noteworthy, as Ang2 is the primary ligand implicated in recruiting and activating TEMs to promote pro-angiogenic functions through Tie2 signaling [16,55], whereas Ang1 classically stabilizes quiescent vessels and acts as a Tie2 agonist in non-malignant contexts [12]. This discrepancy suggests context-dependent, tumor-specific roles for Ang1 in TEMs-rich CRC, potentially involving vessel maturation, pericyte recruitment, or direct enhancement of tumor cell motility (e.g., via ARP2/3 pathway activation in mCRC as mentioned earlier [48], in addition to classical sprouting angiogenesis, and raising the possibility that elevated Ang1 in TEMs-high tumors may support extra mechanisms such as vessel co-option and metastatic dissemination [48].

While direct proof connecting TEMs to the upregulation of CCR7, PDGFRA, CITED2, or CPE in cancer cells is currently lacking, literature-based evidence indicates a potential link between TEMs and the upregulation of CCR7 and PDGFRA, which is possibly mediated through cyclooxygenase-2 (COX-2). Importantly, TEMs have been shown to produce pro-angiogenic factors, including COX-2, at significantly higher levels than other macrophage subsets [16,56], especially upon Ang2-mediated Tie2 receptor activation [16]. Ang2 abundance is noted in hypoxic tumors, produced and released by endothelial cells [57] and cancer cells [58], and is known to participate in TME modulation [58]. Notably, COX-2 has been shown to upregulate CCR7 expression under different conditions [59,60,61]. COX-2 has also been shown to be co-upregulated with PDGFRA and to mediate its constitutive activation in aggressive colonic fibromatosis (colonic desmoid tumors) [62]. In contrast, there is presently no mechanistic evidence demonstrating that COX-2 directly induces transcriptional upregulation of CITED2 or CPE. Nonetheless, because prostaglandin E2 (PGE2), the major COX-2 product, is known to modulate several oncogenic signaling pathways via E-prostanoid receptors (EP1–EP4), including MAPK, NF-κB, cAMP/PKA, PI3K/Akt, and GSK3β–β-catenin axes [63], it remains possible that CITED2 and/or CPE expression in cancer cells may be indirectly influenced through these EP receptor-activated downstream pathways.

The pivotal role of COX-2 in CRC biology is well established. COX-2 is frequently upregulated in colorectal tumors and their stromal compartments, where it catalyzes synthesis of PGE_2_, a bioactive lipid that profoundly remodels the TME. COX-2/PGE2 signaling promotes tumor cell proliferation, survival, and angiogenesis and is implicated in the maintenance and expansion of CRC stem-like cells [64,65]. Furthermore, COX-2/PGE2 signals enhance cell-cycle progression, inhibit apoptosis, stimulate matrix remodeling and increase motility, collectively favoring invasion and metastasis. PGE2 also drives angiogenesis directly and indirectly by upregulating pro-angiogenic mediators within tumor and stromal cells [66,67].

Beyond tumor-intrinsic effects, COX-2/PGE2 exerts potent immunomodulatory actions within the TME. PGE2 suppresses anti-tumor immunity by impairing dendritic cell function, skewing myeloid populations, including TAMs and neutrophils, toward immunosuppressive phenotypes, and enhancing regulatory T-cell activity, changes that promote immune escape and may reduce responsiveness to immunotherapies [65,68]. Clinically, epidemiologic and interventional evidence supports a chemopreventive and therapeutic role for COX inhibition in colorectal neoplasia; regular aspirin and some NSAIDs reduce adenoma formation and CRC incidence, and selective COX-2 inhibitors (coxibs) have demonstrated activity in high-risk syndromes. However, cardiovascular and other safety concerns, together with heterogeneity in tumor COX-2 expression and pathway activation, limit universal application; thus, careful patient selection and biomarker-guided approaches are required. Combining COX-2 pathway antagonists with other targeted or immune therapies is an active area of investigation [69,70]. Given that TEMs represent a major local source of COX-2 within the TME, their infiltration may serve as a potential predictive marker for identifying CRC patients who could benefit from coxib therapy.

Overall, future studies should focus on experimentally validating the proposed TEMs–COX-2–PGE2 regulatory axis and its influence on CCR7, PDGFRA, CITED2, and CPE expression in CRC, for example, by conducting in vitro co-culture experiments using TEMs like macrophage models, tumor-derived organoids, and selective COX-2 or EP-receptor inhibition. Parallel investigations should also dissect the relative contributions of different TEM chemotactic pathways, such as the CXCL12/CXCR4 and Ang2/Tie2 axes in CRC. Ultimately, integrating these mechanistic insights may enable the development of targeted interventions aimed at modulating TEM abundance or activity to improve CRC patients’ outcomes.

## 4. Materials and Methods

**Figure 7:**
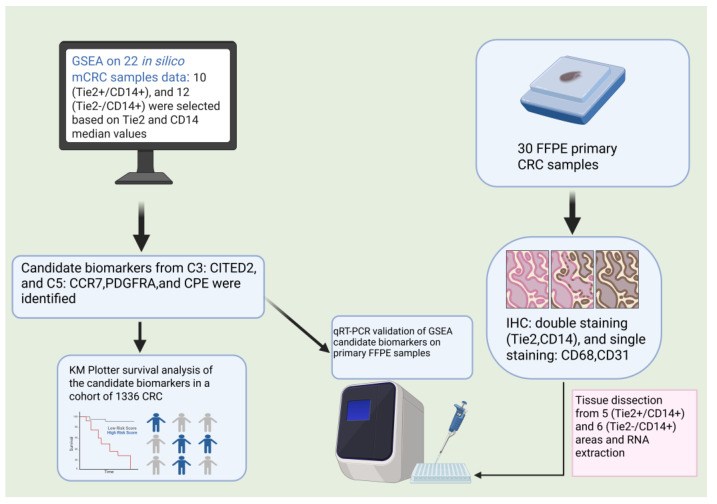
The flowchart summarizes the bioinformatics and experimental workflow used to identify differentially expressed genes between (Tie2^+^/CD14^+^) and (Tie2^−^/CD14^+^) metastatic colorectal cancer (mCRC) of in silico data with validation on FFPE primary CRC samples. The figure was created using BioRender.com (https://app.biorender.com/illustrations/canvas-beta/6927d8bfe89a03e8d476cd12) (accessed on 27 November 2025).

A schematic overview of the research methodology is presented in Figure 7.

### 4.1. FFPE Tissue Specimens from Endoscopic Biopsies of CRC Patients, and Immunohistochemistry

This study included a cohort of 30 patients with CRC, all diagnosed at Sheikh Shakhbout Medical City (SSMC), Abu Dhabi, United Arab Emirates. The clinicopathological characteristics of the included patients are represented in Table 3. Ethical approval was granted by the Institutional Review Board of Sheikh Shakhbout Medical City (SSMC), Abu Dhabi, UAE, number: (SSMC/RES/PP/221). All procedures were carried out in accordance with the ethical standards outlined in the Declaration of Helsinki and the Belmont Report. Tumor staging and classification, including the assessment of tumor, lymph nodes and metastasis (TNM), were conducted under the supervision of two pathologists, I.T. and R.H. The FFPE tissue biopsies analyzed in this study were obtained from patients diagnosed with colorectal adenocarcinoma, including samples from both the colon and rectum. The inclusion criteria were adult patients with a histologically confirmed diagnosis of colorectal adenocarcinoma, no prior history of inflammatory bowel disease (IBD), and each case representing a first-time diagnosis of colorectal neoplasia with verified pathological confirmation. As the primary aim of this study was to investigate the role of TEMs in CRC broadly, anatomical location (colon versus rectum) was not considered as a stratification factor. The 30 FFPE patient samples were cut at 3 µm and placed onto non-charged slides (epredia^®^, Kalamazoo, MI, USA, reference No: AG00008032E01MNZ10) for hematoxylin and eosin histopathological assessment and onto positively charged slides (epredia^®^, USA, reference No: J1800AMNZ) for IHC staining.

#### 4.1.1. Immunohistochemistry

Immunohistochemical staining and quantification of cellular infiltrates in formalin-fixed, paraffin-embedded tissue sections were performed according to previously published protocols [57,71]. Details of the primary antibodies used are summarized in Table 4. In brief, 3 µm tumor sections were deparaffinized and rehydrated, followed by antigen retrieval and suppression of endogenous peroxidase or alkaline phosphatase activity. The sections were then incubated sequentially with the appropriate primary antibodies and enzyme-conjugated secondary antibodies, and signal detection was achieved using the corresponding chromogenic substrates. Sections were counterstained with hematoxylin, dehydrated, mounted with coverslips, and left to air-dry before microscopic examination and obtaining images using an Olympus BX43 microscope (Hachioji-shi, Tokyo, Japan) equipped with an Olympus DP75 camera with Cell Sens Entry Software (v4.4).

#### 4.1.2. Immunohistochemistry Scoring

To quantify TEMs, cells double-stained for Tie2 (red) and CD14 (brown) were counted [72], along with considering macrophage morphology, as some TEMs do not fall in the CD14-positive category [24]. Therefore, all cells that are morphologically consistent with macrophages and are positive for Tie2 were included in TEMs counting, as well [24]. CD68 was used as a pan-macrophage marker, and CD31 staining was used to estimate MVD. After staining, the whole tumor area was thoroughly inspected for the presence of antibody-positive cells, and three randomly selected hot spots for infiltrating immune cells, namely TEMs (Tie2^+^), and TAMs in general (CD68^+^), were identified. A cutoff point of 5% was used: (0–5% positive cells, score 0, negative/absent) and (>5% positive cells, score 1, positive/present) [72].

For MVD scoring, the area of highest MVD, referred to as the vascular “hot spot,” was identified by scanning the entire tumor section. Within this region, all microvessels were counted, and MVD was determined as the total number of vessels across ten random optical fields per section. For analysis, MVD was classified as low (MVD low) or high (MVD high) using a cutoff of 50 microvessels per 10 optical fields [72].

### 4.2. Gene Set Enrichment Analysis

Human CRC tissue transcriptomic profiles were obtained from the publicly available database gene expression omnibus (GEO), accession number GSE41568 [73], (https://www.ncbi.nlm.nih.gov/geo/query/acc.cgi?acc=GSE41568) (accessed on 15 January 2025), and based on grouping of samples using median values for Tie2 and CD14 expression as a cutoff, 10 mCRC Tie2^+^/CD14^+^ profiles vs. 12 mCRC Tie2^−^/CD14^+^ profiles were selected as a study group and a control group, respectively.

To determine the activated and enriched cellular pathways in Tie2^+^/CD14^+^ and Tie2^−^/CD14^+^ groups, absolute gene set enrichment analysis (absGSEA) and standard GSEA were conducted [26]. The expression matrix was analyzed using the absGSEA custom script implemented in R (v4.3.2), which computes sample-specific enrichment scores for each gene set. To ensure reproducibility, the complete R script and corresponding gene set inputs are available upon request. Gene set enrichment was assessed using the Molecular Signatures Database (MSigDB), comprising approximately 90,000 annotated cellular pathways from the Broad Institute “https://www.gsea-msigdb.org (accessed on 15 January 2025)” supplemented with custom pathways. Pathways were considered significantly enriched at a threshold of nominal *p* < 0.05. Selected pathways were further examined to identify differentially enriched genes between the two groups using leading-edge analysis, as detailed in [26]. The resulting gene sets were refined through systematic cross-referencing of genes enriched within statistically significant pathways. Finally, genes recurrently represented across multiple enriched pathways were prioritized for downstream validation.

### 4.3. RNA Extraction

For the RNA extraction from FFPE samples, five regions with (Tie2^+^/CD14^+^) IHC staining as the test group and six regions with (Tie2^−^/CD14^+^) staining as a control group were selected and delineated by two pathologists on reference IHC slides double-stained for Tie2 and CD14. Corresponding areas were identified on unstained 5 µm sections, and 4 slides from each of the selected samples of FFPE tissue biopsies were used and subsequently macro-dissected using sterile needles. The collected tissue fragments were then subjected to RNA extraction using the RecoverAll™ kit (Thermo Fisher Scientific, Waltham, MA, USA) in accordance with the manufacturer’s protocol.

RNA concentrations and purity checks were done using a spectrophotometer (Thermo Fisher Scientific 2000, USA). Selected samples for RT-qPCR validation yielded absorbance values as follows: A260/A280 ratios of 1.93–2.2 and A260/A230 ratios > 1.8. Additionally, all the primers used in RT-qPCR reactions were designed to produce < 150 base pair amplicons, since FFPE samples are known to have short, degraded RNA.

Genomic DNA was removed from all RNA samples by treating the extracted RNA with the TURBO DNAase-free™ Kit (Invitrogen, Carlsbad, CA, USA).

### 4.4. Validation of Selected Candidate Biomarkers Using Quantitative Reverse Transcriptase-PCR (RT-qPCR)

Complementary DNA (cDNA) was synthesized from 400 ng of total RNA using the High-Capacity cDNA Reverse Transcription Kit (Applied Biosystems, Waltham, MA, USA), which incorporates random hexamers primers, according to the manufacturer’s instructions. Gene expression was assessed by quantitative PCR (qPCR) using the Maxima SYBR Green/ROX qPCR Master Mix (2×) (Thermo Fisher Scientific) on a QuantStudio 3 Real-Time PCR System (Applied Biosystems). Primer sequences are provided in Table 5. GAPDH served as a housekeeping gene, and relative target gene expression was determined using the 2^−ΔΔCt^ method, relative to GAPDH.

### 4.5. KM Plot Platform for Survival Analysis of the Selected Validation Biomarkers

To estimate the correlation between the candidate gene level of expression and survival time in CRC patients, an independent CRC cohort comprising 1336 patient samples was evaluated using the KM plot platform to assess the prognostic relevance of each biomarker. This platform integrates gene expression and clinical data from multiple publicly available resources, including GEO (Gene Expression Omnibus), TCGA (The Cancer Genome Atlas), EGA (European Genome-phenome Archive), and GTEx (Genotype-Tissue Expression Project), thus encompassing diverse patient populations across multiple geographic regions [33].

### 4.6. Statistical Analysis

All statistical analyses were conducted using GraphPad Prism 10.6.1 (892), and a *p*-value of 0.05 was determined as a cutoff point for significance. The type of statistical test was chosen according to the data analyzed. For clinicopathological data analysis in relation to TEMs^+^ vs. TEMs^−^ groups, Fisher’s exact test was used. The F-test was used on in silico transcriptomic data to calculate the significance of differential expression of the selected genes between the (Tie2^−^/CD14^+^) and (Tie2^+^/CD14^+^) groups. For analysis of RT-qPCR data, the Mann–Whitney test was chosen.

## 5. Conclusions

**Figure 8:**
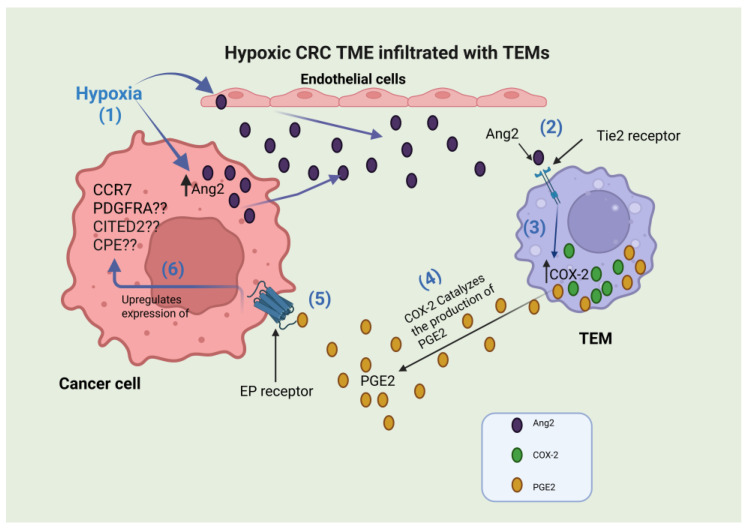
A schematic representation of the hypothetical association between Tie2-expressing macrophages/monocytes (TEMs) and candidate gene upregulation in colorectal cancer (CRC): (1) Hypoxia stimulates the upregulation of angiopoietin2 (Ang2) in endothelial cells [57] and cancer cells [58]. (2) Ang2 binding to Tie2 (tyrosine kinase with immunoglobulin and epidermal growth factor homology domains 2) receptor on Tie2-expressing macrophage (TEM) [16]. (3) Ang2/Tie2 binding reprograms TEM expression profile and upregulates cyclooxygenase2 (COX-2) expression [16]. (4) Elevated COX-2 levels lead to the production of high levels of prostaglandin E2 (PGE2). (5) PGE2 binding to E-prostanoid (EP) receptors [74]. (6) Activation of EP receptors through PGE2 binding is found to be involved directly in chemokine receptor 7 (CCR7) upregulation [59,60] and is co-upregulated with platelet-derived growth factor receptor A (PDGFRA) without proof of a direct link [62], while the COX-2/PGE2 connection to CITED2 (CBP/p300-interacting transactivator 2) and carboxypeptidase E (CPE) is still questionable. Figure created using Biorender.com (https://app.biorender.com/illustrations/692881ee7bd35c729eee69f2) (accessed on 28 November 2025).

This study demonstrates that TEMs constitute a noteworthy immunologic component of the CRC microenvironment, defined by their perivascular enrichment and association with higher tumor grade and size, lymph node metastasis, and increased MVD. Combined in silico analysis of mCRC data and qRT-PCR validation on primary CRC FFPE samples revealed consistent upregulation of CCR7, PDGFRA, CITED2, and CPE in TEMs-high tumors, with CITED2 and CPE additionally correlating significantly with poorer patient survival. These findings support a role for TEMs in fostering tumor-advancing pathways in CRC, which theoretically could be potentiated through the high COX-2 levels produced by TEMs upon Ang2/Tie2 axis activation in hypoxic TME (Figure 8). Furthermore, it identifies TEM-related gene signatures as promising prognostic markers and/or potential therapeutic targets. Continued investigation is required to elucidate the mechanisms underlying TEMs’ function and to evaluate their value in future CRC-targeted therapies.

## 6. Limitations and Future Perspectives

This study is limited by the small sample size (number = 30) for IHC, which restricts generalizability, and the ability to account for tumor heterogeneity or clinicopathological subgroups. Moreover, while qRT-PCR validation was performed, the in silico transcriptomic analysis would benefit from single-cell resolution to better define TEM-specific signatures. Furthermore, while the Discussion provides a potential role of Ang1 in TEMs^+^ CRC, this remains largely associative in nature, as this correlation is derived from observational data of expression levels without direct functional validation through targeted interventions, such as genetic knockdown/overexpression experiments of Ang1 in relevant settings. Finally, survival associations were derived from a large external dataset without direct TEM quantification in those cases. Therefore, validation in larger, prospective cohorts is needed to confirm the prognostic relevance of TEM infiltration and candidate biomarkers (CCR7, PDGFRA, CITED2, CPE, Ang1). Advanced spatial multi-omics (e.g., multiplex immunofluorescence, spatial transcriptomics) and functional studies like co-cultures, organoids, and Tie2-targeted models will help elucidate TEM contributions to angiogenesis, metastasis, and therapy response. These efforts may uncover novel biomarkers or therapeutic strategies, including Ang/Tie2 pathway modulation, to improve outcomes in metastatic CRC.

## Figures and Tables

**Table 1:** Clinicopathological characteristics of patients included in the study.

Variable	Value (%)
Total Number of patients	30
Gender	
Female	13 (43.33%)
Male	17 (56.67%)
Patient age	
60 or less	5 (16.7%)
>60	25 (83.3%)
Pathologic T stage	
T1/T2	13 (43.33%)
T3/T4	17 (56.67%)
Pathologic N stage	
positive	17 (56.67%)
Negative	13 (43.33%)
Histological differentiation	
Well	13 (43.3%)
Moderate/poor	17 (56.7%)
Metastasis	
With	6 (20%)
Without	24 (80%)

**Table 2:** Correlation of Tie2-expressing macrophages in CRC with clinicopathological characteristics of patients, CD14^+^ and CD68^+^ TAM infiltration, and microvessel density (MVD).

Variable	TEMs^+^	TEMs^−^	*p*-Value
No. of patients	18 (60%)	12 (40%)	
Gender			
Female	9 (69.23%)	4 (30.77%)	0.4651
Male	9 (52.94%)	8 (47.06%)	
Patient age			
60 or less	1 (20%)	4 (80%)	0.1282
>60	17 (68%)	8 (32%)	
Pathologic T stage			
T1/T2	2 (15.38%)	11 (84.62%)	<0.0001 ****
T3/T4	16 (94.12%)	1 (5.88%)	
Pathologic N stage			
Positive	16 (94.12%)	1 (5.88%)	<0.0001 ****
Negative	2 (15.38%)	11 (84.62%)	
Histological differentiation			
Well	4 (22.22%)	9 (75%)	0.008 **
Moderate/poor	14 (77.78%)	3 (25%)	
Metastasis			
With	6 (100%)	0 (0%)	0.0568
Without	12 (50%)	12 (5%)	
CD14			
Positive	18 (100%)	12 (100%)	0.99
Negative	0 (0%)	0 (0%)	
CD68			
Positive	18 (100%)	12 (100%)	0.99
Negative	0	0	
MVD			
High	16 (88.89%)	3 (25%)	0.0012 **
Low	2 (11.11%)	9 (75%)	

Data were analyzed using Fisher’s exact test on GraphPad Prism 10.6.1 (892), and a *p*-value of 0.05 was determined as a cutoff point for significance. ** (*p* ≤ 0.01), **** (*p* ≤ 0.0001).

**Table 3:** Patient characteristics for the 30 biopsies collected from CRC patients in the UAE.

No.	Gender	Age	CRC Grade	TNM Stage
1	Female	62	Well differentiated	1
2	Male	58	Well differentiated	1
3	Female	67	Well differentiated	1
4	Male	71	Well differentiated	1
5	Male	55	Well differentiated	1
6	Female	69	Moderately differentiated	3
7	Male	73	Moderately differentiated	3
8	Female	61	Well differentiated	1
9	Male	70	Poorly differentiated	4
10	Female	63	Well differentiated	1
11	Male	76	Poorly differentiated	4
12	Female	68	Moderately differentiated	3
13	Male	59	Well differentiated	1
14	Male	65	Well differentiated	1
15	Female	72	Well differentiated	1
16	Male	63	Well differentiated	1
17	Female	74	Moderately differentiated	3
18	Male	69	Moderately differentiated	3
19	Female	57	Moderately differentiated	3
20	Male	62	Well differentiated	1
21	Female	78	Poorly differentiated	4
22	Male	66	Poorly differentiated	3
23	Male	60	Well differentiated	1
24	Male	75	Poorly differentiated	4
25	Female	68	Moderately differentiated	3
26	Female	64	Moderately differentiated	3
27	Male	77	Poorly differentiated	4
28	Female	71	Poorly differentiated	3
29	Male	69	Moderately differentiated	3
30	Male	73	Poorly differentiated	4

**Table 4:** Antibody details.

Antigen	Monoclonal/Polyclonal	Species	Dilution	Company	Catalog Number
CD68	Monoclonal	Mouse	1:6000	Proteitech^®^ (Rosemont, IL, USA)	66231-2-IG
Tie2	Polyclonal	Rabbit	1:4000	Abcam^®^ (Cambridge, UK)	Ab227219
CD14	Polyclonal	Rabbit	1:2000	Proteitech^®^ (Rosemont, IL, USA)	17000-1-AP
CD31	Monoclonal	Mouse	1:300	Leica Microsystems^®^ (Wetzlar, Germany)	NCL-CD31-1A10

**Table 5:** Sequences of qPCR validation.

Gene ID	Forward Primer	Reverse Primer
*CCR7*	CTCTCCTTGTCATTTTCCAG	ACAAAGTGTAGTCCACTGTG
*PDGFRA*	CTTTCGCCAAAGTGGAGGAG	AGCCACCGTGAGTTCAGAAC
*CITED2*	ATGGCAGACCATATGATGGC	TGCTGCTGCTGGTGGTGA
*CPE*	GATCCACAGTACCCGCATTC	ACCAGTCCTTGAGTTCACCA
*Ang1*	GATTTCCAAAGAGGCTGGAAGG	GTACTGCCTCTGACTGGTAATG
*Ang2*	GTGCTGGAGAACATCATGGAAA	CTGCATTCTGCTGTATCTCTAC
*GAPDH*	TGTCAGTGGTGGACCTGACCT	TCGCTGTTGAAGTCAGACGAG

## Data Availability

The transcriptomic data used in this work has been obtained from the Gene Expression Omnibus “https://www.ncbi.nlm.nih.gov/geo (accessed on 15 January 2025)”, accession number: GSE41568. All other supporting data of this study are either included in the manuscript or available on request from the corresponding author. Please turn to the CRediT taxonomy for the term explanation.

## Supplementary Materials

The following supporting information can be downloaded at: https://www.mdpi.com/article/10.3390/ijms27083645/s1.
