# Supplementary material for: Integrative In Silico and FFPE Tissue Analyses Elucidate Upregulated Genes in Colorectal Cancer Enriched for Tie2-Expressing Macrophages/Monocytes
Source: Int J Mol Sci. 2026 Apr 19;27(8):3645. doi: 10.3390/ijms27083645 (PMC13116895; doi:10.3390/ijms27083645)
Supplement: Supplementary file 1 [file ijms-27-03645-s001.zip › ijms-4182376-supplementary.pdf]

Note: all pathways can be checked at (https://www.gsea-msigdb.org)

Table S1

Gene sets used (C1-C8) from the MSigDB database and their description

Gene Set Used	Description
C1	Positional Gene Sets
C2	Curated Gene Sets
C3	Regulatory-Related Gene Sets
C4	Computational Gene Sets
C5	Gene Ontology Gene Sets (Biological Processes, Molecular Functions, and Cellular Components)
C6	Oncogenic Related Gene Sets
C7	Immunology-Related Gene Sets
C8	Cell Type Signature Gene Sets

Table S2

Significantly enriched pathways from C1

Number	Enriched Pathway
1	chr10q26
2	chr12q21
3	chr1q23
4	chr2p16
5	chr5q35
6	chr6p12

Table S3

Significantly enriched pathways from C2

Number	Enriched Pathway
1	ACEVEDO_METHYLATED_IN_LIVER_CANCER_DN
2	ALONSO_METASTASIS_EMT_UP
3	ALONSO_METASTASIS_UP
4	BAKKER_FOXO3_TARGETS_DN
5	BANDRES_RESPONSE_TO_CARMUSTIN_MGMT_48HR_DN
6	BARRIER_CANCER_RELAPSE_NORMAL_SAMPLE_UP

7	BECKER_TAMOXIFEN_RESISTANCE_DN
8	BECKER_TAMOXIFEN_RESISTANCE_UP
9	BENPORATH_ES_WITH_H3K27ME3
10	BENPORATH_OCT4_TARGETS
11	BENPORATH_PRC2_TARGETS
12	BENPORATH_SUZ12_TARGETS
13	BILD_E2F3_ONCOGENIC_SIGNATURE
14	BLALOCK_ALZHEIMERS_DISEASE_INCIPIENT_UP
15	BLALOCK_ALZHEIMERS_DISEASE_UP
16	BLANCO_MELO_BETA_INTERFERON_TREATED_BRONCHIAL_EPITHELIAL_CELLS_DN
17	BLANCO_MELO_MERS_COV_INFECTION_MCR5_CELLS_UP
18	BONOME_OVARIAN_CANCER_SURVIVAL_OPTIMAL_DEBULKING
19	BOYAULT_LIVER_CANCER_SUBCLASS_G2
20	BOYLAN_MULTIPLE_MYELOMA_C_CLUSTER_DN
21	BOYLAN_MULTIPLE_MYELOMA_C_D_DN
22	BRIDEAU_IMPRINTED_GENES
23	BROWNE_HCMV_INFECTION_1HR_DN
24	BROWNE_HCMV_INFECTION_4HR_DN
25	BROWNE_HCMV_INFECTION_6HR_DN
26	BRUINS_UVC_RESPONSE_VIA_TP53_GROUP_C
27	BYSTRYKH_HEMATOPOIESIS_STEM_CELL_QTL_TRANS
28	CHARAFE_BREAST_CANCER_LUMINAL_VS_MESENCHYMAL_UP
29	CHYLA_CBFA2T3_TARGETS_UP
30	COLDREN_GEFITINIB_RESISTANCE_DN
31	DACOSTA_ERCC3_ALLELE_XPCS_VS_TTD_UP
32	DARWICHE_PAPILLOMA_RISK_HIGH_DN
	DARWICHE_SKIN_TUMOR_PROMOTER_DN
33	DARWICHE_SQUAMOUS_CELL_CARCINOMA_DN
34	DAVICIONI_MOLECULAR_ARMS_VS_ERMS_UP
35	DODD_NASOPHARYNGEAL_CARCINOMA_UP
36	DURAND_STROMA_NS_UP
37	ENK_UV_RESPONSE_EPIDERMIS_UP
38	EPPERT_CE_HSC_LSC
39	EPPERT_HSC_R
40	FIGUEROA_AML_METHYLATION_CLUSTER_1_DN
41	FIRESTEIN_CTNNB1_PATHWAY
42	FLORIO_NEOCORTEX_BASAL_RADIAL_GLIA_UP
43	FOSTER_KDM1A_TARGETS_UP
44	GARGALOVIC_RESPONSE_TO_OXIDIZED_PHOSPHOLIPIDS_PINK_DN
45	GAUSSMANN_MLL_AF4_FUSION_TARGETS_G_UP
46	GRABARCZYK_BCL11B_TARGETS_UP
47	GUTIERREZ_CHRONIC_LYMPHOCYTIC_LEUKEMIA_DN

48	HANN_RESISTANCE_TO_BCL2_INHIBITOR_UP
49	HAN_SATB1_TARGETS_DN
50	HATADA_METHYLATED_IN_LUNG_CANCER_UP
51	HELLER_HDAC_TARGETS_UP
52	HERNANDEZ_MITOTIC_ARREST_BY_DOCETAXEL_2_UP
53	HOLLERN_MICROACINAR_BREAST_TUMOR_UP
54	HUTTMANN_B_CLL_POOR_SURVIVAL_UP
55	IBRAHIM_NRF2_DOWN
56	IWANAGA_CARCINOGENESIS_BY_KRAS_DN
57	JAEGER_METASTASIS_DN
58	JI_METASTASIS_REPRESSED_BY_STK11
59	KATSANOU_ELAVL1_TARGETS_DN
60	KIM_ALL_DISORDERS_OLIGODENDROCYTE_NUMBER_CORR_UP
61	KIM_WT1_TARGETS_8HR_DN
62	KONDO_PROSTATE_CANCER_WITH_H3K27ME3
63	KRASNOSELSKAYA_ILF3_TARGETS_DN
64	KYNG_DNA_DAMAGE_BY_GAMMA_RADIATION
65	KYNG_DNA_DAMAGE_UP
66	LANDIS_ERBB2_BREAST_TUMORS_324_UP
67	LEE_BMP2_TARGETS_UP
68	LEE_TARGETS_OF_PTCH1_AND_SUFU_DN
69	LEIN_CEREBELLUM_MARKERS
70	LIEN_BREAST_CARCINOMA_METAPLASTIC_VS_DUCTAL_DN
71	LIM_MAMMARY_LUMINAL_MATURE_UP
72	LIU_LIVER_CANCER
73	LIU_PROSTATE_CANCER_UP
74	LI_PROSTATE_CANCER_EPIGENETIC
75	LOPEZ_MBD_TARGETS
76	MAGRANGEAS_MULTIPLE_MYELOMA_IGLL_VS_IGLK_UP
77	MARTENS_TRETINOIN_RESPONSE_UP
78	MARTINEZ_TP53_TARGETS_DN
79	MARTIN_VIRAL_GPCR_SIGNALING_DN
80	MATSUDA_NATURAL_KILLER_DIFFERENTIATION
81	MCCABE_BOUND_BY_HOXC6
82	MEISSNER_BRAIN_HCP_WITH_H3K27ME3
83	MEISSNER_BRAIN_HCP_WITH_H3K4ME3_AND_H3K27ME3
84	MEISSNER_NPC_HCP_WITH_H3K4ME2
85	MEISSNER_NPC_HCP_WITH_H3_UNMETHYLATED
86	MIKKELSEN_ES_ICP_WITH_H3K4ME3
87	MIKKELSEN_ES_ICP_WITH_H3K4ME3_AND_H3K27ME3
88	MIKKELSEN_ES_LCP_WITH_H3K4ME3
89	MIKKELSEN_IPS_LCP_WITH_H3K4ME3

90	MIKKELSEN_IPS_WITH_HCP_H3K27ME3
91	MIKKELSEN_MCV6_HCP_WITH_H3K27ME3
92	MIKKELSEN_MCV6_ICP_WITH_H3K27ME3
93	MIKKELSEN_MEF_HCP_WITH_H3K27ME3
94	MIKKELSEN_MEF_ICP_WITH_H3K27ME3
95	MIKKELSEN_NPC_HCP_WITH_H3K4ME3_AND_H3K27ME3
96	MOREAUX_MULTIPLE_MYELOMA_BY_TACI_UP
97	MULLIGHAN_NPM1_MUTATED_SIGNATURE_1_DN
98	MULLIGHAN_NPM1_MUTATED_SIGNATURE_1_UP
99	MULLIGHAN_NPM1_MUTATED_SIGNATURE_2_UP
100	MULLIGHAN_NPM1_SIGNATURE_3_DN
101	MULLIGHAN_NPM1_SIGNATURE_3_UP
102	NIKOLSKY_BREAST_CANCER_12Q13_Q21_AMPLICON
103	NIKOLSKY_BREAST_CANCER_16P13_AMPLICON
104	NOURUZI_NEPC_ASCL1_TARGETS
105	ODONNELL_TFRC_TARGETS_UP
106	OHM_METHYLATED_IN_ADULT_CANCERS
107	OUELLET_CULTURED_OVARIAN_CANCER_INVASIVE_VS_LMP_UP
108	PEREZ_TP63_TARGETS
109	PLASARI_TGFB1_SIGNALING_VIA_NFIC_10HR_DN
110	PURBEY_TARGETS_OF_CTBP1_NOT_SATB1_DN
111	RAO_BOUND_BY_SALL4_ISOFORM_A
112	RICKMAN_HEAD_AND_NECK_CANCER_A
113	RIGGINS_TAMOXIFEN_RESISTANCE_UP
114	RIZKI_TUMOR_INVASIVENESS_3D_UP
115	ROVERSI_GLIOMA_COPY_NUMBER_DN
116	ROVERSI_GLIOMA_LOH_REGIONS
117	SAFFORD_T_LYMPHOCYTE_ANERGY
118	SCHAEFFER_PROSTATE_DEVELOPMENT_48HR_UP
119	SHEN_SMARCA2_TARGETS_DN
120	SHETH_LIVER_CANCER_VS_TXNIP_LOSS_PAM1
121	SMID_BREAST_CANCER_ERBB2_UP
122	TANAKA_METHYLATED_IN_ESOPHAGEAL_CARCINOMA
123	VALK_AML_CLUSTER_1
124	VALK_AML_WITH_FLT3_ITD
125	VERHAAK_GLIOBLASTOMA_PRONEURAL
126	WANG_MLL_TARGETS
127	XU_GH1_AUTOCRINE_TARGETS_UP
128	XU_GH1_EXOGENOUS_TARGETS_UP
129	YAMASHITA_LIVER_CANCER_WITH_EPCAM_UP
130	YANG_BCL3_TARGETS_UP
131	YAUCH_HEDGEHOG_SIGNALING_PARACRINE_DN

132	YAUCH_HEDGEHOG_SIGNALING_PARACRINE_UP
133	ZHANG_TARGETS_OF_EWSR1_FLI1_FUSION
134	ZHAN_LATE_DIFFERENTIATION_GENES_UP
135	ZHAN_MULTIPLE_MYELOMA_LB_UP
136	ZWANG_EGF_INTERVAL_DN
137	ZWANG_TRANSIENTLY_UP_BY_2ND_EGF_PULSE_ONLY
138	SIG_PIP3_SIGNALING_IN_CARDIAC_MYOCTES
139	KEGG_ADHERENS_JUNCTION
140	KEGG_ALDOSTERONE_REGULATED_SODIUM_REABSORPTION
141	KEGG_ENDOCYTOSIS
142	KEGG_ERBB_SIGNALING_PATHWAY
143	KEGG_GLUTATHIONE_METABOLISM
144	KEGG_GNRH_SIGNALING_PATHWAY
145	KEGG_LONG_TERM_DEPRESSION
146	KEGG_NEUROACTIVE_LIGAND_RECEPTOR_INTERACTION
147	KEGG_TIGHT_JUNCTION
148	KEGG_VIBRIO_CHOLERAE_INFECTION
149	KEGG_MEDICUS_REFERENCE_GF_RTK_PI3K_SIGNALING_PATHWAY
150	KEGG_MEDICUS_REFERENCE_GF_RTK_RAS_ERK_SIGNALING_PATHWAY
151	KEGG_MEDICUS_REFERENCE_RTK_PLCG_ITPR_SIGNALING_PATHWAY
152	PID_ARF6_PATHWAY
153	PID_FGF_PATHWAY
154	REACTOME_CELL_CELL_COMMUNICATION
155	REACTOME_CLASS_A_1_RHODOPSIN_LIKE_RECEPTORS
156	REACTOME_CONSTITUTIVE_SIGNALING_BY_ABERRANT_PI3K_IN_CANCER
157	REACTOME_GAP_JUNCTION_TRAFFICKING_AND_REGULATION
158	REACTOME_GLUTATHIONE_CONJUGATION
159	REACTOME_GPCR_LIGAND_BINDING
160	REACTOME_G_ALPHA_Q_SIGNALLING_EVENTS
161	REACTOME_INTRA_GOLGI_TRAFFIC
162	REACTOME_MAP2K_AND_MAPK_ACTIVATION
163	REACTOME_MAPK_FAMILY_SIGNALING_CASCADES
164	REACTOME_NEURONAL_SYSTEM
165	REACTOME_NEUROTRANSMITTER_RECEPTORS_AND_POSTSYNAPTIC_SIGNAL_TRANSMISSION
166	REACTOME_ONCOGENIC_MAPK_SIGNALING
167	REACTOME_POTASSIUM_CHANNELS
168	REACTOME_RAB_GEFS_EXCHANGE_GTP_FOR_GDP_ON_RABS
169	REACTOME_RHOU_GTPASE_CYCLE
170	REACTOME_TRANSMISSION_ACROSS_CHEMICAL_SYNAPSES
171	REACTOME_VESICLE_MEDIATED_TRANSPORT
172	WP_EGFR_TYROSINE_KINASE_INHIBITOR_RESISTANCE
173	WP_GPCRS_OTHER

174	WP_HAIR_FOLLICLE_DEVELOPMENT_CYTODIFFERENTIATION_PART_3_OF_3
175	WP_HIPPO_SIGNALING_REGULATION_PATHWAYS
176	WP_METABOLIC_PATHWAYS_OF_FIBROBLASTS
177	WP_NETRIN_UNC5B_SIGNALING_PATHWAY
178	WP_PRE_IMPLANTATION_EMBRYO
179	WP_RAS_SIGNALING
180	WP_SYNAPTIC_VESICLE_PATHWAY
181	WP_THERMOGENESIS

Table S4

Significantly enriched pathways from C3

**Number**	**Enriched Pathway**
1	LET_7C_3P
2	MIR10226
3	MIR10399_5P
4	MIR1197
5	MIR1202
6	MIR12128
7	MIR12129
8	MIR1224_3P
9	MIR1244
10	MIR1248
11	MIR124_5P
12	MIR1252_5P
13	MIR1266_5P
14	MIR1275
15	MIR1276
16	MIR1298_5P
17	MIR1321

18	MIR143_3P_MIR4770
19	MIR185_5P
20	MIR194_3P
21	MIR198
22	MIR2110
23	MIR2113
24	MIR219A_5P
25	MIR2355_5P
26	MIR26A_1_3P
27	MIR26A_2_3P
28	MIR302A_3P_MIR302B_3P_MIR302C_3P_MIR302D_3P
29	MIR3059_5P
30	MIR3121_3P
31	MIR3121_5P
32	MIR3142
33	MIR3144_3P
34	MIR3182
35	MIR320A_5P
36	MIR323A_3P
37	MIR326
38	MIR3529_3P
39	MIR3618
40	MIR363_5P
41	MIR3692_3P
42	MIR372_3P
43	MIR373_3P
44	MIR3915
45	MIR3928_3P
46	MIR3972
47	MIR3974
48	MIR412_3P
49	MIR424_5P
50	MIR4256
51	MIR4274
52	MIR4303
53	MIR4306
54	MIR4469
55	MIR4476
56	MIR4478
57	MIR4492
58	MIR4500
59	MIR450A_1_3P

60	MIR450A_2_3P
61	MIR4510
62	MIR4516
63	MIR4517
64	MIR4524A_3P
65	MIR4525
66	MIR452_3P
67	MIR4533
68	MIR4644
69	MIR4667_5P
70	MIR4675
71	MIR4679
72	MIR4682
73	MIR4700_5P
74	MIR4725_3P
75	MIR4726_3P
76	MIR4728_5P
77	MIR4731_5P
78	MIR4733_3P
79	MIR4739
80	MIR4741
81	MIR4756_5P
82	MIR4761_5P
83	MIR4782_3P
84	MIR4790_3P
85	MIR4795_3P
86	MIR4795_5P
87	MIR4796_5P
88	MIR486_3P
89	MIR497_5P
90	MIR4999_5P
91	MIR5004_5P
92	MIR5006_5P
93	MIR5010_5P
94	MIR503_3P
95	MIR5093
96	MIR5193
97	MIR519A_2_5P_MIR520B_5P
98	MIR520A_3P
99	MIR520B_3P_MIR520C_3P_MIR520E_3P
100	MIR520D_3P
101	MIR548AS_3P

102	MIR552_3P
103	MIR556_3P
104	MIR5586_5P
105	MIR592
106	MIR597_3P
107	MIR606
108	MIR6071
109	MIR6074
110	MIR6077
111	MIR610
112	MIR6127
113	MIR6129
114	MIR6130
115	MIR6133
116	MIR631
117	MIR6504_3P
118	MIR653_5P
119	MIR654_3P
120	MIR6716_5P
121	MIR6766_3P
122	MIR6769A_5P
123	MIR6769B_5P
124	MIR6780B_5P
125	MIR6785_5P
126	MIR6815_5P
127	MIR6838_3P
128	MIR6838_5P
129	MIR6848_5P
130	MIR6857_5P
131	MIR6865_5P
132	MIR6868_5P
133	MIR6873_3P
134	MIR6876_5P
135	MIR6883_5P
136	MIR7109_5P
137	MIR7110_3P
138	MIR7152_3P
139	MIR764
140	MIR8075
141	MIR8077
142	MIR8089
143	MIR934

144	MIR9500
145	MIR9718
146	AACATTC_MIR4093P
147	ACATTCC_MIR1_MIR206
148	CACCAGC_MIR138
149	CAGCCTC_MIR4855P
150	CCTGAGT_MIR510
151	CTCTGGA_MIR520A_MIR525
152	GACTGTT_MIR212_MIR132
153	GTGGTGA_MIR197
154	TCATCTC_MIR143
155	TCTGGAC_MIR198
156	TGCACTT_MIR519C_MIR519B_MIR519A
157	BDP1_TARGET_GENES
158	CBX7_TARGET_GENES
159	CENPT_TARGET_GENES
160	CUX1_TARGET_GENES
161	GLI1_TARGET_GENES
162	GREB1_TARGET_GENES
163	IGLV5_37_TARGET_GENES
164	INSM2_TARGET_GENES
165	LMTK3_TARGET_GENES
166	MAFG_TARGET_GENES
167	METTL14_TARGET_GENES
168	MIER1_TARGET_GENES
169	MZF1_TARGET_GENES
170	NCOA2_TARGET_GENES
171	NFKBIA_TARGET_GENES
172	NKX2_5_TARGET_GENES
173	PHB2_TARGET_GENES
174	SIX1_TARGET_GENES
175	SMN1_SMN2_TARGET_GENES
176	SOX10_TARGET_GENES
177	SS18_SSX1_FUSION_UNIPROT_Q8IZH1_UNREVIEWED_TARGET_GENES
178	TBPL1_TARGET_GENES
179	ZBTB18_TARGET_GENES
180	ZFP36L1_TARGET_GENES
181	ZIM3_TARGET_GENES
182	ZNF165_TARGET_GENES
183	ZNF197_TARGET_GENES
184	ZNF202_TARGET_GENES

185	ZNF22_TARGET_GENES
186	ZNF274_TARGET_GENES
187	ZNF320_TARGET_GENES
188	ZNF37A_TARGET_GENES
189	ZNF436_TARGET_GENES
190	ZNF512_TARGET_GENES
191	ZNF529_TARGET_GENES
192	ZNF549_TARGET_GENES
193	ZNF561_TARGET_GENES
194	ZNF577_TARGET_GENES
195	ZNF584_TARGET_GENES
196	ZNF589_TARGET_GENES
197	ZNF660_TARGET_GENES
198	ZNF766_TARGET_GENES
199	ZNF768_TARGET_GENES
200	ZNF92_TARGET_GENES
201	ZSCAN4_TARGET_GENES
202	AAANWWTGC_UNKNOWN
203	ACCTGTTG_UNKNOWN
204	AHR_Q5
205	AMEF2_Q6
206	AP1_01
207	AP1_C
208	AP4_Q5
209	AREB6_01
210	AREB6_02
211	AREB6_03
212	AREB6_04
213	AR_Q6
214	CACCCBINDINGFACTOR_Q6
215	CAGGTA_AREB6_01
216	CATRRAGC_UNKNOWN
217	CCCNNGGGAR_OLF1_01
218	CCCNNNNNNAAGWT_UNKNOWN
219	CDP_02
220	CDX2_Q5
221	CEBPB_02
222	CEBPDELTA_Q6
223	CEBP_C
224	CEBP_Q2
225	CEBP_Q3
226	CHOP_01

227	CHX10_01
228	COMP1_01
229	CP2_02
230	CRX_Q4
231	CTGYNNCTYTAA_UNKNOWN
232	CTTTAAR_UNKNOWN
233	CTTTGA_LEF1_Q2
234	E12_Q6
235	E2A_Q2
236	ELK1_01
237	ERR1_Q2
238	ER_Q6_01
239	ER_Q6_02
240	EVI1_05
241	FOXM1_01
242	FXR_IR1_Q6
243	FXR_Q3
244	GATA1_02
245	GATA1_04
246	GATA1_05
247	GATA3_01
248	GATA6_01
249	GATAAGR_GATA_C
250	GCANCTGNY_MYOD_Q6
251	GCM_Q2
252	GFI1_01
253	GGGNRMNNYCAT_UNKNOWN
254	GGGTGGRR_PAX4_03
255	GGGYGTGNY_UNKNOWN
256	GTGGGTGK_UNKNOWN
257	GTTGNYNNRGNAAC_UNKNOWN
258	HAND1E47_01
259	HEN1_02
260	HOXA4_Q2
261	IRF1_01
262	LFA1_Q6
263	LMO2COM_01
264	LXR_DR4_Q3
265	MEF2_03
266	MEF2_Q6_01
267	MEIS1BHOXA9_01
268	MYB_Q3

269	MYOD_Q6
270	MYOD_Q6_01
271	MYOGENIN_Q6
272	NFE2_01
273	NFY_Q6
274	NKX61_01
275	OCT1_05
276	OCT1_Q6
277	OSF2_Q6
278	PAX8_B
279	PBX1_01
280	PTF1BETA_Q6
281	PU1_Q6
282	PXR_Q2
283	RACCACAR_AML_Q6
284	RFX1_02
285	RNGTGGGC_UNKNOWN
286	RTAAACA_FREAC2_01
287	RTTTNNNYTGGM_UNKNOWN
288	S8_01
289	SMAD4_Q6
290	STAT5A_04
291	STAT_Q6
292	TAATTA_CHX10_01
293	TAAYNRNNTCC_UNKNOWN
294	TAL1ALPHAE47_01
295	TATA_C
296	TCANNTGAY_SREBP1_01
297	TCCATTKW_UNKNOWN
298	TCF4_Q5
299	TGACCTTG_SF1_Q6
300	TGACCTY_ERR1_Q2
301	TGANTCA_AP1_C
302	TGATTTRY_GFI1_01
303	TGGNNNNNNKCCAR_UNKNOWN
304	TGTTTGY_HNF3_Q6
305	TTANTCA_UNKNOWN
306	TTCYRGAA_UNKNOWN
307	TTF1_Q6
308	USF_02
309	WCAANNNYCAG_UNKNOWN
310	WGGAATGY_TEF1_Q6

311	WTTGKCTG_UNKNOWN
312	YAATNANRNNNCAG_UNKNOWN
313	YATTNATC_UNKNOWN
314	YCATTAA_UNKNOWN
315	YGCANTGCR_UNKNOWN
316	ZIC3_01
317	ZID_01

Table S5

Significantly enriched pathways from C4

Number	Enriched pathway
**1**	GCM_FANCC
**2**	GCM_HMGA2
**3**	GCM_PRKCG
**4**	GCM_PTPRU
**5**	GCM_SMARCD1
**6**	GCM_SUPT4H1
**7**	MORF_CAMK4
**8**	MORF_DCC
**9**	MORF_FSHR
**10**	MORF_IFNA1
**11**	MORF_IL4
**12**	MORF_MAGEA8
**13**	MORF_MAGEA9
**14**	MORF_MDM2
**15**	MORF_PRKCA
**16**	MORF_RAD51L3
**17**	MORF_SUPT3H

**18**	MORF_TFDP2
**19**	MORF_TNFRSF25
**20**	MORF_TNFRSF6
**21**	MODULE_104
**22**	MODULE_13
**23**	MODULE_136
**24**	MODULE_139
**25**	MODULE_163
**26**	MODULE_174
**27**	MODULE_179
**28**	MODULE_180
**29**	MODULE_181
**30**	MODULE_192
**31**	MODULE_199
**32**	MODULE_241
**33**	MODULE_242
**34**	MODULE_279
**35**	MODULE_291
**36**	MODULE_33
**37**	MODULE_334
**38**	MODULE_341
**39**	MODULE_350
**40**	MODULE_37
**41**	MODULE_379
**42**	MODULE_41
**43**	MODULE_411
**44**	MODULE_427
**45**	MODULE_455
**46**	MODULE_480
**47**	MODULE_521
**48**	MODULE_525
**49**	MODULE_67
**50**	MODULE_69
**51**	MODULE_7
**52**	MODULE_85
**53**	MODULE_94
**54**	MODULE_95

Table S6

Significantly enriched pathways from C5

Number	Enriched pathway
1	GOBP_ACTIN_FILAMENT_BASED_MOVEMENT
2	GOBP_ACTIN_FILAMENT_BUNDLE_ORGANIZATION
3	GOBP_ACTIN_FILAMENT_ORGANIZATION
4	GOBP_ACTION_POTENTIAL
5	GOBP_ANATOMICAL_STRUCTURE_MATURATION
6	GOBP_APOPTOTIC_PROCESS
7	GOBP_BIOLOGICAL_PROCESS_INVOLVED_IN_INTERACTION_WITH_HOST
8	GOBP_BIOLOGICAL_PROCESS_INVOLVED_IN_SYMBIOTIC_INTERACTION
9	GOBP_CALCIUM_MEDIATED_SIGNALING
10	GOBP_CAMERA_TYPE_EYE_MORPHOGENESIS
11	GOBP_CARBOHYDRATE_DERIVATIVE_BIOSYNTHETIC_PROCESS
12	GOBP_CARDIAC_MUSCLE_CELL_ACTION_POTENTIAL
13	GOBP_CELLULAR_RESPONSE_TO_ALCOHOL
14	GOBP_CELLULAR_RESPONSE_TO_ORGANIC_CYCLIC_COMPOUND
15	GOBP_CELL_CELL_JUNCTION_ASSEMBLY
16	GOBP_CELL_CELL_JUNCTION_ORGANIZATION
17	GOBP_CELL_CELL_RECOGNITION
18	GOBP_CELL_CELL_SIGNALING
19	GOBP_CELL_COMMUNICATION_INVOLVED_IN_CARDIAC_CONDUCTION
20	GOBP_CELL_MATURATION
21	GOBP_CELL_PROJECTION_ASSEMBLY
22	GOBP_CYTOSKELETON_ORGANIZATION
23	GOBP_DETECTION_OF_STIMULUS
24	GOBP_DEVELOPMENTAL_MATURATION
25	GOBP_EMBRYONIC_PLACENTA_DEVELOPMENT
26	GOBP_ENDOMEMBRANE_SYSTEM_ORGANIZATION
27	GOBP_ENTRY_INTO_HOST
28	GOBP_EPITHELIAL_CELL_DIFFERENTIATION
29	GOBP_EPITHELIAL_CELL_PROLIFERATION
30	GOBP_ERK1_AND_ERK2_CASCADE
31	GOBP_ERYTHROCYTE_HOMEOSTASIS
32	GOBP_ESTABLISHMENT_OF_CELL_POLARITY
33	GOBP_ESTABLISHMENT_OR_MAINTENANCE_OF_CELL_POLARITY
34	GOBP_EXOCYTOSIS
35	GOBP_EXPORT_FROM_CELL
36	GOBP_FLUID_TRANSPORT
37	GOBP_GAMETE_GENERATION
38	GOBP_GLAND_DEVELOPMENT
39	GOBP_GLYCOPROTEIN_BIOSYNTHETIC_PROCESS
40	GOBP_GLYCOPROTEIN_METABOLIC_PROCESS
41	GOBP_G_PROTEIN_COUPLED_RECEPTOR_SIGNALING_PATHWAY
42	GOBP_HINDBRAIN_DEVELOPMENT

**43**	GOBP_HIPPO_SIGNALING
**44**	GOBP_HOMOTYPIC_CELL_CELL_ADHESION
**45**	GOBP_HORMONE_TRANSPORT
**46**	GOBP_ICOSANOID_BIOSYNTHETIC_PROCESS
**47**	GOBP_ICOSANOID_SECRETION
**48**	GOBP_ICOSANOID_TRANSPORT
**49**	GOBP_INFLAMMASOME_MEDIATED_SIGNALING_PATHWAY
**50**	GOBP_INTRACELLULAR_RECEPTOR_SIGNALING_PATHWAY
**51**	GOBP_IN_UTERO_EMBRYONIC_DEVELOPMENT
**52**	GOBP_JNK_CASCADE
**53**	GOBP_LABYRINTHINE_LAYER_DEVELOPMENT
**54**	GOBP_LAMELLIPODIUM_ORGANIZATION
**55**	GOBP_LEUKOCYTE_ADHESION_TO_VASCULAR_ENDOTHELIAL_CELL
**56**	GOBP_LOCALIZATION_WITHIN_MEMBRANE
**57**	GOBP_LOCOMOTORY_BEHAVIOR
**58**	GOBP_LONG_TERM_SYNAPTIC_POTENTIATION
**59**	GOBP_L_GLUTAMATE_IMPORT
**60**	GOBP_MAMMARY_GLAND_DEVELOPMENT
**61**	GOBP_MAMMARY_GLAND_EPITHELIUM_DEVELOPMENT
**62**	GOBP_MAPK_CASCADE
**63**	GOBP_MEMBRANE_ORGANIZATION
**64**	GOBP_MEMBRANE_REPOLARIZATION
**65**	GOBP_MODULATION_BY_SYMBIONT_OF_ENTRY_INTO_HOST
**66**	GOBP_MONOATOMIC_CATION_TRANSMEMBRANE_TRANSPORT
**67**	GOBP_MONOATOMIC_CATION_TRANSPORT
**68**	GOBP_MONOATOMIC_ION_TRANSMEMBRANE_TRANSPORT
**69**	GOBP_MONOATOMIC_ION_TRANSPORT
**70**	GOBP_MULTICELLULAR_ORGANISMAL_LEVEL_HOMEOSTASIS
**71**	GOBP_MULTICELLULAR_ORGANISM_REPRODUCTION
**72**	GOBP_MYELOID_CELL_HOMEOSTASIS
**73**	GOBP_NEGATIVE_REGULATION_OF_INTRACELLULAR_PROTEIN_TRANSPORT
**74**	GOBP_NEGATIVE_REGULATION_OF_MAPK_CASCADE
**75**	GOBP_NEGATIVE_REGULATION_OF_MUSCLE_CELL_APOPTOTIC_PROCESS
**76**	GOBP_NEGATIVE_REGULATION_OF_ORGANELLE_ASSEMBLY
**77**	GOBP_NEGATIVE_REGULATION_OF_PEPTIDYL_TYROSINE_PHOSPHORYLATION
**78**	GOBP_NEGATIVE_REGULATION_OF_PLASMA_MEMBRANE_BOUNDED_CELL_PROJECTION_ASSEMBLY
**79**	GOBP_NEGATIVE_REGULATION_OF_PROGRAMMED_CELL_DEATH
**80**	GOBP_NEGATIVE_REGULATION_OF_STEM_CELL_PROLIFERATION
**81**	GOBP_NERVOUS_SYSTEM_PROCESS
**82**	GOBP_NEUROTRANSMITTER_SECRETION
**83**	GOBP_NEUROTRANSMITTER_TRANSPORT
**84**	GOBP_OLIGODENDROCYTE_DEVELOPMENT

**85**	GOBP_OLIGODENDROCYTE_DIFFERENTIATION
**86**	GOBP_PEPTIDE_TRANSPORT
**87**	GOBP_PEPTIDYL_AMINO_ACID_MODIFICATION
**88**	GOBP_PEPTIDYL_TYROSINE_MODIFICATION
**89**	GOBP_PHOSPHOLIPID_TRANSLOCATION
**90**	GOBP_PHOSPHORYLATION
**91**	GOBP_PLACENTA_DEVELOPMENT
**92**	GOBP_PLASMA_MEMBRANE_ORGANIZATION
**93**	GOBP_POSITIVE_REGULATION_OF_ACTIN_FILAMENT_BUNDLE_ASSEMBLY
**94**	GOBP_POSITIVE_REGULATION_OF_CELLULAR_COMPONENT_BIOGENESIS
**95**	GOBP_POSITIVE_REGULATION_OF_CELLULAR_COMPONENT_ORGANIZATION
**96**	GOBP_POSITIVE_REGULATION_OF_CELL_MATRIX_ADHESION
**97**	GOBP_POSITIVE_REGULATION_OF_CELL_SUBSTRATE_ADHESION
**98**	GOBP_POSITIVE_REGULATION_OF_CYTOSKELETON_ORGANIZATION
**99**	GOBP_POSITIVE_REGULATION_OF_ESTABLISHMENT_OF_PROTEIN_LOCALIZATION
**100**	GOBP_POSITIVE_REGULATION_OF_HEART_GROWTH
**101**	GOBP_POSITIVE_REGULATION_OF_HORMONE_SECRETION
**102**	GOBP_POSITIVE_REGULATION_OF_JNK_CASCADE
**103**	GOBP_POSITIVE_REGULATION_OF_LAMELLIPODIUM_ORGANIZATION
**104**	GOBP_POSITIVE_REGULATION_OF_MAPK_CASCADE
**105**	GOBP_POSITIVE_REGULATION_OF_MONOATOMIC_ION_TRANSMEMBRANE_TRANSPORT
**106**	GOBP_POSITIVE_REGULATION_OF_MONOATOMIC_ION_TRANSPORT
**107**	GOBP_POSITIVE_REGULATION_OF_ORGANELLE_ORGANIZATION
**108**	GOBP_POSITIVE_REGULATION_OF_OSTEOBLAST_DIFFERENTIATION
**109**	GOBP_POSITIVE_REGULATION_OF_PATTERN_RECOGNITION_RECEPTOR_SIGNALING_PATHWAY
**110**	GOBP_POSITIVE_REGULATION_OF_PHOSPHORUS_METABOLIC_PROCESS
**111**	GOBP_POSITIVE_REGULATION_OF_PLASMA_MEMBRANE_BOUNDED_CELL_PROJECTION_ASSEMBLY
**112**	GOBP_POSITIVE_REGULATION_OF_PROTEIN_CONTAINING_COMPLEX_ASSEMBLY
**113**	GOBP_POSITIVE_REGULATION_OF_PROTEIN_LOCALIZATION
**114**	GOBP_POSITIVE_REGULATION_OF_PROTEIN_LOCALIZATION_TO_CELL_PERIPHERY
**115**	GOBP_POSITIVE_REGULATION_OF_PROTEIN_LOCALIZATION_TO_MEMBRANE
**116**	GOBP_POSITIVE_REGULATION_OF_PROTEIN_LOCALIZATION_TO_PLASMA_MEMBRANE
**117**	GOBP_POSITIVE_REGULATION_OF_PROTEIN_POLYMERIZATION
**118**	GOBP_POSITIVE_REGULATION_OF_PROTEIN_SECRETION
**119**	GOBP_POSITIVE_REGULATION_OF_REPRODUCTIVE_PROCESS
**120**	GOBP_POSITIVE_REGULATION_OF_SECRETION
**121**	GOBP_POSITIVE_REGULATION_OF_SIGNALING
**122**	GOBP_POSITIVE_REGULATION_OF_SMALL_GTPASE_MEDIATED_SIGNAL_TRANSDUCTION
**123**	GOBP_POSITIVE_REGULATION_OF_STRESS_FIBER_ASSEMBLY
**124**	GOBP_POSITIVE_REGULATION_OF_SYNAPTIC_TRANSMISSION
**125**	GOBP_POSITIVE_REGULATION_OF_TRANSCRIPTION_BY_RNA_POLYMERASE_II

**126**	GOBP_POSITIVE_REGULATION_OF_TRANSMEMBRANE_TRANSPORT
**127**	GOBP_POTASSIUM_ION_TRANSPORT
**128**	GOBP_PROSTAGLANDIN_TRANSPORT
**129**	GOBP_PROTEIN_AUTOPHOSPHORYLATION
**130**	GOBP_PROTEIN_CONTAINING_COMPLEX_ASSEMBLY
**131**	GOBP_PROTEIN_LOCALIZATION_TO_CELL_PERIPHERY
**132**	GOBP_PROTEIN_LOCALIZATION_TO_PLASMA_MEMBRANE
**133**	GOBP_PROTEIN_PHOSPHORYLATION
**134**	GOBP_PROTEIN_POLYMERIZATION
**135**	GOBP_RAS_PROTEIN_SIGNAL_TRANSDUCTION
**136**	GOBP_REGULATION_OF_ACTION_POTENTIAL
**137**	GOBP_REGULATION_OF_ACUTE_INFLAMMATORY_RESPONSE
**138**	GOBP_REGULATION_OF_ANATOMICAL_STRUCTURE_SIZE
**139**	GOBP_REGULATION_OF_BIOLOGICAL_PROCESS_INVOLVED_IN_SYMBIOTIC_INTERACTION
**140**	GOBP_REGULATION_OF_CELLULAR_COMPONENT_SIZE
**141**	GOBP_REGULATION_OF_CELLULAR_LOCALIZATION
**142**	GOBP_REGULATION_OF_CELLULAR_RESPONSE_TO_OXIDATIVE_STRESS
**143**	GOBP_REGULATION_OF_CELL_DIVISION
**144**	GOBP_REGULATION_OF_CELL_PROJECTION_ASSEMBLY
**145**	GOBP_REGULATION_OF_CELL_SIZE
**146**	GOBP_REGULATION_OF_DNA_BINDING
**147**	GOBP_REGULATION_OF_EPITHELIAL_CELL_DIFFERENTIATION
**148**	GOBP_REGULATION_OF_EPITHELIAL_CELL_PROLIFERATION
**149**	GOBP_REGULATION_OF_ESTABLISHMENT_OF_PROTEIN_LOCALIZATION
**150**	GOBP_REGULATION_OF_EXOCYTOSIS
**151**	GOBP_REGULATION_OF_FATTY_ACID_TRANSPORT
**152**	GOBP_REGULATION_OF_GLYCOPROTEIN_METABOLIC_PROCESS
**153**	GOBP_REGULATION_OF_HORMONE_SECRETION
**154**	GOBP_REGULATION_OF_INTRACELLULAR_SIGNAL_TRANSDUCTION
**155**	GOBP_REGULATION_OF_JNK_CASCADE
**156**	GOBP_REGULATION_OF_KINASE_ACTIVITY
**157**	GOBP_REGULATION_OF_LEUKOCYTE_ADHESION_TO_VASCULAR_ENDOTHELIAL_CELL
**158**	GOBP_REGULATION_OF_LONG_TERM_SYNAPTIC_POTENTIATION
**159**	GOBP_REGULATION_OF_MEMBRANE_LIPID_DISTRIBUTION
**160**	GOBP_REGULATION_OF_MEMBRANE_POTENTIAL
**161**	GOBP_REGULATION_OF_MICROTUBULE_POLYMERIZATION
**162**	GOBP_REGULATION_OF_MITOCHONDRIAL_MEMBRANE_POTENTIAL
**163**	GOBP_REGULATION_OF_MONOATOMIC_ION_TRANSMEMBRANE_TRANSPORT
**164**	GOBP_REGULATION_OF_MONOATOMIC_ION_TRANSPORT
**165**	GOBP_REGULATION_OF_PEPTIDYL_TYROSINE_PHOSPHORYLATION
**166**	GOBP_REGULATION_OF_PHOSPHORUS_METABOLIC_PROCESS
**167**	GOBP_REGULATION_OF_PHOSPHORYLATION
**168**	GOBP_REGULATION_OF_PROTEIN_CONTAINING_COMPLEX_ASSEMBLY

**169**	GOBP_REGULATION_OF_PROTEIN_LOCALIZATION_TO_MEMBRANE
**170**	GOBP_REGULATION_OF_PROTEIN_LOCALIZATION_TO_PLASMA_MEMBRANE
**171**	GOBP_REGULATION_OF_PROTEIN_MODIFICATION_PROCESS
**172**	GOBP_REGULATION_OF_PROTEIN_SECRETION
**173**	GOBP_REGULATION_OF_PROTEIN_TYROSINE_KINASE_ACTIVITY
**174**	GOBP_REGULATION_OF_RAS_PROTEIN_SIGNAL_TRANSDUCTION
**175**	GOBP_REGULATION_OF_REPRODUCTIVE_PROCESS
**176**	GOBP_REGULATION_OF_RESPONSE_TO_OXIDATIVE_STRESS
**177**	GOBP_REGULATION_OF_RESPONSE_TO_STRESS
**178**	GOBP_REGULATION_OF_RHO_PROTEIN_SIGNAL_TRANSDUCTION
**179**	GOBP_REGULATION_OF_SMALL_GTPASE_MEDIATED_SIGNAL_TRANSDUCTION
**180**	GOBP_REGULATION_OF_STRIATED_MUSCLE_CONTRACTION
**181**	GOBP_REGULATION_OF_SUBSTRATE_ADHESION_DEPENDENT_CELL_SPREADING
**182**	GOBP_REGULATION_OF_SUPEROXIDE_METABOLIC_PROCESS
**183**	GOBP_REGULATION_OF_TRANSMEMBRANE_TRANSPORT
**184**	GOBP_REGULATION_OF_TRANSPORTER_ACTIVITY
**185**	GOBP_RENAL_SYSTEM_PROCESS
**186**	GOBP_REPRODUCTION
**187**	GOBP_RESPONSE_TO_ABIOTIC_STIMULUS
**188**	GOBP_RESPONSE_TO_CALCIUM_ION
**189**	GOBP_RESPONSE_TO_CATECHOLAMINE
**190**	GOBP_RESPONSE_TO_ESTRADIOL
**191**	GOBP_SCF_DEPENDENT_PROTEASOMAL_UBIQUITIN_DEPENDENT_PROTEIN_CATABOLIC_PROCESS
**192**	GOBP_SENSORY_PERCEPTION
**193**	GOBP_SENSORY_PERCEPTION_OF_MECHANICAL_STIMULUS
**194**	GOBP_SIGNAL_RELEASE
**195**	GOBP_SMALL_GTPASE_MEDIATED_SIGNAL_TRANSDUCTION
**196**	GOBP_SUBSTRATE_ADHESION_DEPENDENT_CELL_SPREADING
**197**	GOBP_SYNAPTIC_VESICLE_EXOCYTOSIS
**198**	GOBP_TISSUE_HOMEOSTASIS
**199**	GOBP_TRABECULA_FORMATION
**200**	GOBP_TRABECULA_MORPHOGENESIS
**201**	GOBP_TRANSMEMBRANE_RECEPTOR_PROTEIN_TYROSINE_KINASE_SIGNALING_PATHWAY
**202**	GOBP_TRANSMEMBRANE_TRANSPORT
**203**	GOBP_VENTRICULAR_CARDIAC_MUSCLE_CELL_ACTION_POTENTIAL
**204**	GOBP_VESICLE_CYTOSKELETAL_TRAFFICKING
**205**	GOBP_VESICLE_DOCKING
**206**	GOBP_VIRAL_LIFE_CYCLE
**207**	GOBP_VIRAL_TRANSCRIPTION
**208**	GOCC_ACTIN_BASED_CELL_PROJECTION
**209**	GOCC_APICAL_PART_OF_CELL
**210**	GOCC_CELL_BODY

**211**	GOCC_CELL_CELL_JUNCTION
**212**	GOCC_CELL_PROJECTION_MEMBRANE
**213**	GOCC_CILIARY_BASE
**214**	GOCC_CIS_GOLGI_NETWORK
**215**	GOCC_DENDRITIC_TREE
**216**	GOCC_EARLY_ENDOSOME
**217**	GOCC_EXOCYTIC_VESICLE
**218**	GOCC_GOLGI_APPARATUS
**219**	GOCC_GOLGI_ASSOCIATED_VESICLE_MEMBRANE
**220**	GOCC_LEADING_EDGE_MEMBRANE
**221**	GOCC_MICROVILLUS
**222**	GOCC_MYOSIN_COMPLEX
**223**	GOCC_NEURON_TO_NEURON_SYNAPSE
**224**	GOCC_NUCLEAR_ENVELOPE
**225**	GOCC_NUCLEAR_MEMBRANE
**226**	GOCC_PERINUCLEAR_REGION_OF_CYTOPLASM
**227**	GOCC_PLASMA_MEMBRANE_REGION
**228**	GOCC_POSTSYNAPSE
**229**	GOCC_POSTSYNAPTIC_DENSITY_MEMBRANE
**230**	GOCC_POSTSYNAPTIC_MEMBRANE
**231**	GOCC_POSTSYNAPTIC_SPECIALIZATION
**232**	GOCC_PRESYNAPTIC_ACTIVE_ZONE
**233**	GOCC_PRESYNAPTIC_MEMBRANE
**234**	GOCC_RUFFLE_MEMBRANE
**235**	GOCC_SOMATODENDRITIC_COMPARTMENT
**236**	GOCC_SYNAPTIC_MEMBRANE
**237**	GOMF_ACTIN_BINDING
**238**	GOMF_ACTIN_FILAMENT_BINDING
**239**	GOMF_ALCOHOL_BINDING
**240**	GOMF_CELL_ADHESION_MEDIATOR_ACTIVITY
**241**	GOMF_CELL_CELL_ADHESION_MEDIATOR_ACTIVITY
**242**	GOMF_CHOLESTEROL_BINDING
**243**	GOMF_CLATHRIN_BINDING
**244**	GOMF_DNA_BINDING_TRANSCRIPTION_ACTIVATOR_ACTIVITY
**245**	GOMF_EXOGENOUS_PROTEIN_BINDING
**246**	GOMF_EXOPEPTIDASE_ACTIVITY
**247**	GOMF_GATED_CHANNEL_ACTIVITY
**248**	GOMF_GUANYL_NUCLEOTIDE_EXCHANGE_FACTOR_ACTIVITY
**249**	GOMF_G_PROTEIN_COUPLED_RECEPTOR_ACTIVITY
**250**	GOMF_LIPASE_ACTIVITY
**251**	GOMF_METALLOEXOPEPTIDASE_ACTIVITY
**252**	GOMF_MICROFILAMENT_MOTOR_ACTIVITY
**253**	GOMF_MOLECULAR_ADAPTOR_ACTIVITY

**254**	GOMF_MOLECULAR_TRANSDUCER_ACTIVITY
**255**	GOMF_MONOATOMIC_CATION_CHANNEL_ACTIVITY
**256**	GOMF_MONOATOMIC_ION_TRANSMEMBRANE_TRANSPORTER_ACTIVITY
**257**	GOMF_PALMITOYLTRANSFERASE_ACTIVITY
**258**	GOMF_PASSIVE_TRANSMEMBRANE_TRANSPORTER_ACTIVITY
**259**	GOMF_PEPTIDASE_ACTIVITY
**260**	GOMF_PHOSPHATASE_BINDING
**261**	GOMF_PHOSPHATIDYLINOSITOL_BINDING
**262**	GOMF_PHOSPHATIDYLINOSITOL_PHOSPHATE_BINDING
**263**	GOMF_PHOSPHOLIPASE_ACTIVITY
**264**	GOMF_PHOSPHOLIPASE_C_ACTIVITY
**265**	GOMF_PHOSPHOLIPID_BINDING
**266**	GOMF_PHOSPHORIC_DIESTER_HYDROLASE_ACTIVITY
**267**	GOMF_PHOSPHORIC_ESTER_HYDROLASE_ACTIVITY
**268**	GOMF_POTASSIUM_CHANNEL_ACTIVITY
**269**	GOMF_PROTEIN_KINASE_ACTIVITY
**270**	GOMF_PROTEIN_MACROMOLECULE_ADAPTOR_ACTIVITY
**271**	GOMF_PROTEIN_PHOSPHATASE_BINDING
**272**	GOMF_PROTEIN_TYROSINE_KINASE_ACTIVITY
**273**	GOMF_PROTEIN_TYROSINE_KINASE_BINDING
**274**	GOMF_SALT_TRANSMEMBRANE_TRANSPORTER_ACTIVITY
**275**	GOMF_SIGNALING_RECEPTOR_BINDING
**276**	GOMF_TRANSMEMBRANE_RECEPTOR_PROTEIN_KINASE_ACTIVITY
**277**	GOMF_TRANSMEMBRANE_RECEPTOR_PROTEIN_TYROSINE_KINASE_ACTIVITY
**278**	GOMF_VOLTAGE_GATED_MONOATOMIC_CATION_CHANNEL_ACTIVITY
**279**	GOMF_ZINC_ION_BINDING
**280**	HP_11_PAIRS_OF_RIBS
**281**	HP_2_3_TOE_SYNDACTYLY
**282**	HP_ABNORMALITIES_OF_PLACENTA_OR_UMBILICAL_CORD
**283**	HP_ABNORMALITY_OF_DENTAL_ERUPTION
**284**	HP_ABNORMALITY_OF_DIGESTIVE_SYSTEM_MORPHOLOGY
**285**	HP_ABNORMALITY_OF_FLUID_REGULATION
**286**	HP_ABNORMALITY_OF_IMMUNE_SYSTEM_PHYSIOLOGY
**287**	HP_ABNORMALITY_OF_LIMB_BONE
**288**	HP_ABNORMALITY_OF_REPRODUCTIVE_SYSTEM_PHYSIOLOGY
**289**	HP_ABNORMALITY_OF_SALIVATION
**290**	HP_ABNORMALITY_OF_THE_AMNIOTIC_FLUID
**291**	HP_ABNORMALITY_OF_THE_AUDITORY_CANAL
**292**	HP_ABNORMALITY_OF_THE_CHIN
**293**	HP_ABNORMALITY_OF_THE_CHOANAE
**294**	HP_ABNORMALITY_OF_THE_DIENCEPHALON
**295**	HP_ABNORMALITY_OF_THE_GENITAL_SYSTEM
**296**	HP_ABNORMALITY_OF_THE_MIDDLE_EAR

**297**	HP_ABNORMALITY_OF_THE_ORBITAL_REGION
**298**	HP_ABNORMALITY_OF_THE_PERIPHERAL_NERVOUS_SYSTEM
**299**	HP_ABNORMALITY_OF_THE_SPLEEN
**300**	HP_ABNORMALITY_OF_THE_TONGUE
**301**	HP_ABNORMALITY_OF_THE_TONSILS
**302**	HP_ABNORMALITY_OF_THE_UMBILICAL_CORD
**303**	HP_ABNORMALITY_OF_THE_UPPER_URINARY_TRACT
**304**	HP_ABNORMALITY_OF_THE_WRIST
**305**	HP_ABNORMALITY_OF_TIBIA_MORPHOLOGY
**306**	HP_ABNORMAL_1ST_METACARPAL_MORPHOLOGY
**307**	HP_ABNORMAL_BASAL_GANGLIA_MORPHOLOGY
**308**	HP_ABNORMAL_BLOOD_SODIUM_CONCENTRATION
**309**	HP_ABNORMAL_CEREBROSPINAL_FLUID_MORPHOLOGY
**310**	HP_ABNORMAL_CHOROID_MORPHOLOGY
**311**	HP_ABNORMAL_CLAVICLE_MORPHOLOGY
**312**	HP_ABNORMAL_CORNEAL_STROMA_MORPHOLOGY
**313**	HP_ABNORMAL_CORNEA_MORPHOLOGY
**314**	HP_ABNORMAL_CORTICAL_BONE_MORPHOLOGY
**315**	HP_ABNORMAL_CRANIAL_NERVE_PHYSIOLOGY
**316**	HP_ABNORMAL_EATING_BEHAVIOR
**317**	HP_ABNORMAL_EXTERNAL_NOSE_MORPHOLOGY
**318**	HP_ABNORMAL_EYELID_MORPHOLOGY
**319**	HP_ABNORMAL_FACIAL_SKELETON_MORPHOLOGY
**320**	HP_ABNORMAL_FETAL_MORPHOLOGY
**321**	HP_ABNORMAL_FOOT_MORPHOLOGY
**322**	HP_ABNORMAL_FRONTAL_BONE_MORPHOLOGY
**323**	HP_ABNORMAL_HALLUX_MORPHOLOGY
**324**	HP_ABNORMAL_HALLUX_PHALANX_MORPHOLOGY
**325**	HP_ABNORMAL_HAND_MORPHOLOGY
**326**	HP_ABNORMAL_INTESTINE_MORPHOLOGY
**327**	HP_ABNORMAL_JAW_MORPHOLOGY
**328**	HP_ABNORMAL_JOINT_MORPHOLOGY
**329**	HP_ABNORMAL_LONG_BONE_MORPHOLOGY
**330**	HP_ABNORMAL_LUNG_DEVELOPMENT
**331**	HP_ABNORMAL_MOVEMENTS_OF_FACE_AND_HEAD
**332**	HP_ABNORMAL_NASAL_DORSUM_MORPHOLOGY
**333**	HP_ABNORMAL_ORAL_PHYSIOLOGY
**334**	HP_ABNORMAL_PATTERN_OF_RESPIRATION
**335**	HP_ABNORMAL_PERIPHERAL_MYELINATION
**336**	HP_ABNORMAL_POSTERIOR_EYE_SEGMENT_MORPHOLOGY
**337**	HP_ABNORMAL_PYRAMIDAL_SIGN
**338**	HP_ABNORMAL_REFLEX
**339**	HP_ABNORMAL_RESPIRATORY_SYSTEM_MORPHOLOGY

**340**	HP_ABNORMAL_RESPIRATORY_SYSTEM_PHYSIOLOGY
**341**	HP_ABNORMAL_RETINAL_MORPHOLOGY
**342**	HP_ABNORMAL_RIB_MORPHOLOGY
**343**	HP_ABNORMAL_SACCADIC_EYE_MOVEMENTS
**344**	HP_ABNORMAL_SACRUM_MORPHOLOGY
**345**	HP_ABNORMAL_SOCIAL_BEHAVIOR
**346**	HP_ABNORMAL_TENDON_MORPHOLOGY
**347**	HP_ABNORMAL_THUMB_MORPHOLOGY
**348**	HP_ABNORMAL_TOE_MORPHOLOGY
**349**	HP_ABNORMAL_TONGUE_MORPHOLOGY
**350**	HP_ABNORMAL_URETER_PHYSIOLOGY
**351**	HP_ADULT_ONSET
**352**	HP_AGENESIS_OF_INCISOR
**353**	HP_ANNULAR_PANCREAS
**354**	HP_APHASIA
**355**	HP_APLASIA_HYPOPLASIA_AFFECTING_THE_FUNDUS
**356**	HP_APLASIA_HYPOPLASIA_INVOLVING_BONES_OF_THE_THORAX
**357**	HP_APLASIA_HYPOPLASIA_OF_THE_CLAVICLES
**358**	HP_APLASIA_HYPOPLASIA_OF_THE_LUNGS
**359**	HP_APLASIA_HYPOPLASIA_OF_THE_NIPPLES
**360**	HP_APLASIA_HYPOPLASIA_OF_THE_OPTIC_NERVE
**361**	HP_APLASIA_HYPOPLASIA_OF_THE_RIBS
**362**	HP_AREFLEXIA_OF_LOWER_LIMBS
**363**	HP_BABINSKI_SIGN
**364**	HP_BILATERAL_PTOSIS
**365**	HP_BLURRED_VISION
**366**	HP_BROAD_TOE
**367**	HP_CARDIAC_ARREST
**368**	HP_CARDIAC_CONDUCTION_ABNORMALITY
**369**	HP_CENTRAL_HYPOTHYROIDISM
**370**	HP_CHILDHOOD_ONSET
**371**	HP_CHOANAL_STENOSIS
**372**	HP_CLINODACTYLY
**373**	HP_COLOR_VISION_DEFECT
**374**	HP_CONGENITAL_ONSET
**375**	HP_CONGENITAL_SENSORINEURAL_HEARING_IMPAIRMENT
**376**	HP_CORNEAL_OPACITY
**377**	HP_CUTANEOUS_SYNDACTYLY
**378**	HP_DECREASED_ABSENT_ANKLE_REFLEXES
**379**	HP_DECREASED_BODY_WEIGHT
**380**	HP_DECREASED_FERTILITY
**381**	HP_DECREASED_NUMBER_OF_PERIPHERAL_MYELINATED_NERVE_FIBERS
**382**	HP_DEHYDRATION

**383**	HP_DENTAL_MALOCCLUSION
**384**	HP_DEVIATION_OF_THE_HAND_OR_OF_FINGERS_OF_THE_HAND
**385**	HP_DIABETES_MELLITUS
**386**	HP_DISTAL_SENSORY_IMPAIRMENT
**387**	HP_DUPLICATION_OF_HAND_BONES
**388**	HP_DYSMETRIA
**389**	HP_DYSPNEA
**390**	HP_DYSTONIA
**391**	HP_EEG_WITH_POLYSPIKE_WAVE_COMPLEXES
**392**	HP_EEG_WITH_SPIKE_WAVE_COMPLEXES
**393**	HP_ESODEVIATION
**394**	HP_EXCESSIVE_SALIVATION
**395**	HP_FACIAL_ASYMMETRY
**396**	HP_FAILURE_TO_THRIVE
**397**	HP_FAILURE_TO_THRIVE_IN_INFANCY
**398**	HP_FETAL_ANOMALY
**399**	HP_FINGER_SYNDACTYLY
**400**	HP_FUNCTIONAL_ABNORMALITY_OF_THE_INNER_EAR
**401**	HP_GAIT_ATAXIA
**402**	HP_GASTROINTESTINAL_ATRESIA
**403**	HP_GASTROINTESTINAL_INFLAMMATION
**404**	HP_GASTROSTOMY_TUBE_FEEDING_IN_INFANCY
**405**	HP_GENERALIZED_ABNORMALITY_OF_SKIN
**406**	HP_HEART_BLOCK
**407**	HP_HEMIPLEGIA_HEMIPARESIS
**408**	HP_HIP_DYSPLASIA
**409**	HP_HORIZONTAL_NYSTAGMUS
**410**	HP_HYDROCEPHALUS
**411**	HP_HYPERTELORISM
**412**	HP_HYPOHIDROSIS
**413**	HP_HYPOPLASTIC_HEART
**414**	HP_HYPOPLASTIC_NIPPLES
**415**	HP_IMPAIRED_PROPRIOCEPTION
**416**	HP_IMPAIRED_SOCIAL_INTERACTIONS
**417**	HP_IMPAIRED_VIBRATION_SENSATION_IN_THE_LOWER_LIMBS
**418**	HP_IMPAIRED_VIBRATORY_SENSATION
**419**	HP_INFERTILITY
**420**	HP_INTELLECTUAL_DISABILITY_MODERATE
**421**	HP_INTESTINAL_MALROTATION
**422**	HP_JOINT_STIFFNESS
**423**	HP_LACRIMATION_ABNORMALITY
**424**	HP_LOWER_LIMB_HYPERREFLEXIA
**425**	HP_MACROGLOSSIA

**426**	HP_MACROTIA
**427**	HP_MALNUTRITION
**428**	HP_MANDIBULAR_PROGNATHIA
**429**	HP_MIDDLE_AGE_ONSET
**430**	HP_MIGRAINE
**431**	HP_NASAL_CONGESTION
**432**	HP_NEONATAL_DEATH
**433**	HP_NEONATAL_RESPIRATORY_DISTRESS
**434**	HP_NEOPLASM
**435**	HP_NON_MENDELIAN_INHERITANCE
**436**	HP_NON_MIDLINE_CLEFT_LIP
**437**	HP_OPHTHALMOPARESIS
**438**	HP_ORAL_PHARYNGEAL_DYSPHAGIA
**439**	HP_OROFACIAL_CLEFT
**440**	HP_PERIPHERAL_AXONAL_DEGENERATION
**441**	HP_POINTED_CHIN
**442**	HP_POLYDACTYLY
**443**	HP_POLYPHAGIA
**444**	HP_POOR_SUCK
**445**	HP_PREMATURE_LOSS_OF_TEETH
**446**	HP_PRIMARY_AMENORRHEA
**447**	HP_PTERYGIUM
**448**	HP_PTOSIS
**449**	HP_RADIOULNAR_SYNOSTOSIS
**450**	HP_RECURRENT_URINARY_TRACT_INFECTIONS
**451**	HP_REGIONAL_ABNORMALITY_OF_SKIN
**452**	HP_RESPIRATORY_INSUFFICIENCY
**453**	HP_SANDAL_GAP
**454**	HP_SELECTIVE_TOOTH_AGENESIS
**455**	HP_SEVERE_INFECTION
**456**	HP_SHORT_RIBS
**457**	HP_SKELETAL_MUSCLE_HYPERTROPHY
**458**	HP_SOMATIC_SENSORY_DYSFUNCTION
**459**	HP_SPASTIC_PARAPLEGIA
**460**	HP_SPEECH_ARTICULATION_DIFFICULTIES
**461**	HP_SPORADIC
**462**	HP_STENOSIS_OF_THE_EXTERNAL_AUDITORY_CANAL
**463**	HP_SUDDEN_DEATH
**464**	HP_SYNDACTYLY
**465**	HP_SYNOSTOSIS_INVOLVING_BONES_OF_THE_UPPER_LIMBS
**466**	HP_SYNOSTOSIS_OF_JOINTS
**467**	HP_TETRAPLEGIA_TETRAPARESIS
**468**	HP_TOE_DEFORMITY

**469**	HP_TOE_SYNDACTYLY
**470**	HP_TREMOR_BY_ANATOMICAL_SITE
**471**	HP_UNILATERAL_RENAL_AGENESIS
**472**	HP_URETHRAL_OBSTRUCTION
**473**	HP_VERTEBRAL_SEGMENTATION_DEFECT
**474**	HP_VOCAL_CORD_PARALYSIS
**475**	HP_WEAKNESS_DUE_TO_UPPER_MOTOR_NEURON_DYSFUNCTION
**476**	HP_YOUNG_ADULT_ONSET

Table S7

Significantly enriched pathways from C6

Number	Enriched pathway
**1**	ALK_DN.V1_DN
**2**	ATF2_S_UP.V1_UP
**3**	ATM_DN.V1_DN
**4**	BCAT.100_UP.V1_UP
**5**	BCAT_GDS748_UP
**6**	BRCA1_DN.V1_DN
**7**	BRCA1_DN.V1_UP
**8**	CAHOY_NEURONAL
**9**	CAHOY_OLIGODENDROCUTIC
**10**	CRX_DN.V1_UP
**11**	CRX_NRL_DN.V1_UP
**12**	CYCLIN_D1_UP.V1_DN
**13**	DCA_UP.V1_UP
**14**	GCNP_SHH_UP_EARLY.V1_DN
**15**	GCNP_SHH_UP_LATE.V1_DN
**16**	IL15_UP.V1_DN
**17**	IL21_UP.V1_DN
**18**	IL21_UP.V1_UP
**19**	IL2_UP.V1_DN
**20**	JNK_DN.V1_UP
**21**	KRAS.DF.V1_DN
**22**	KRAS.LUNG_UP.V1_DN
**23**	LEF1_UP.V1_DN
**24**	MEK_UP.V1_UP
**25**	NRL_DN.V1_UP
**26**	P53_DN.V2_DN
**27**	PGF_UP.V1_DN
**28**	PRC2_SUZ12_UP.V1_UP
**29**	PTEN_DN.V1_UP

**30**	RAPA_EARLY_UP.V1_UP
**31**	RELA_DN.V1_DN
**32**	STK33_DN
**33**	STK33_NOMO_DN
**34**	TGFB_UP.V1_DN
**35**	VEGF_A_UP.V1_UP
**36**	WNT_UP.V1_DN

Table S8

Significantly enriched pathways from C7

Number	Enriched pathway
**1**	GOLDRATH_EFF_VS_MEMORY_CD8_TCELL_DN
**2**	GSE10094_LCMV_VS_LISTERIA_IND_EFF_CD4_TCELL_DN
**3**	GSE10239_KLRG1INT_VS_KLRG1HIGH_EFF_CD8_TCELL_UP
**4**	GSE10239_MEMORY_VS_DAY4.5_EFF_CD8_TCELL_UP
**5**	GSE10239_MEMORY_VS_KLRG1HIGH_EFF_CD8_TCELL_UP
**6**	GSE11057_NAIVE_VS_CENT_MEMORY_CD4_TCELL_UP
**7**	GSE1112_OT1_CD8AB_VS_HY_CD8AA_THYMOCYTE_RTOC_CULTURE_UP
**8**	GSE11367_CTRL_VS_IL17_TREATED_SMOOTH_MUSCLE_CELL_UP
**9**	GSE11864_CSF1_PAM3CYS_VS_CSF1_IFNG_PAM3CYS_IN_MAC_UP
**10**	GSE11864_CSF1_VS_CSF1_PAM3CYS_IN_MAC_UP
**11**	GSE11924_TFH_VS_TH1_CD4_TCELL_UP
**12**	GSE11924_TH1_VS_TH17_CD4_TCELL_DN
**13**	GSE11924_TH1_VS_TH17_CD4_TCELL_UP
**14**	GSE11961_FOLLICULAR_BCELL_VS_MARGINAL_ZONE_BCELL_UP
**15**	GSE11961_GERMINAL_CENTER_BCELL_DAY7_VS_MEMORY_BCELL_DAY40_DN
**16**	GSE11961_GERMINAL_CENTER_BCELL_DAY7_VS_MEMORY_BCELL_DAY40_UP
**17**	GSE11961_MARGINAL_ZONE_BCELL_VS_GERMINAL_CENTER_BCELL_DAY40_UP
**18**	GSE11961_MARGINAL_ZONE_BCELL_VS_GERMINAL_CENTER_BCELL_DAY7_DN
**19**	GSE11961_MARGINAL_ZONE_BCELL_VS_GERMINAL_CENTER_BCELL_DAY7_UP
**20**	GSE11961_MARGINAL_ZONE_BCELL_VS_MEMORY_BCELL_DAY40_DN
**21**	GSE11961_MEMORY_BCELL_DAY40_VS_GERMINAL_CENTER_BCELL_DAY40_UP
**22**	GSE11961_MEMORY_BCELL_DAY7_VS_MEMORY_BCELL_DAY40_UP
**23**	GSE11961_PLASMA_CELL_DAY7_VS_GERMINAL_CENTER_BCELL_DAY40_DN
**24**	GSE11961_PLASMA_CELL_DAY7_VS_GERMINAL_CENTER_BCELL_DAY40_UP
**25**	GSE11961_PLASMA_CELL_DAY7_VS_MEMORY_BCELL_DAY40_UP
**26**	GSE12003_4D_VS_8D_CULTURE_MIR223_KO_BM_PROGENITOR_UP
**27**	GSE12003_MIR223_KO_VS_WT_BM_PROGENITOR_4D_CULTURE_DN
**28**	GSE12003_MIR223_KO_VS_WT_BM_PROGENITOR_4D_CULTURE_UP
**29**	GSE12198_CTRL_VS_LOW_IL2_STIM_NK_CELL_DN

**30**	GSE12198_LOW_IL2_STIM_NK_CELL_VS_HIGH_IL2_STIM_NK_CELL_DN
**31**	GSE12198_LOW_IL2_STIM_NK_CELL_VS_HIGH_IL2_STIM_NK_CELL_UP
**32**	GSE12366_NAIVE_VS_MEMORY_BCELL_UP
**33**	GSE12484_HEALTHY_VS_PERIDONTITIS_NEUTROPHILS_UP
**34**	GSE12505_WT_VS_E2_2_HET_PDC_DN
**35**	GSE13306_LAMINA_PROPRIA_VS_SPLEEN_TREG_DN
**36**	GSE13306_RA_VS_UNTREATED_MEM_CD4_TCELL_UP
**37**	GSE13306_TREG_RA_VS_TCONV_RA_DN
**38**	GSE13306_TREG_RA_VS_TCONV_RA_UP
**39**	GSE13411_IGM_VS_SWITCHED_MEMORY_BCELL_UP
**40**	GSE13411_NAIVE_VS_IGM_MEMORY_BCELL_DN
**41**	GSE13411_NAIVE_VS_IGM_MEMORY_BCELL_UP
**42**	GSE13411_NAIVE_VS_MEMORY_BCELL_UP
**43**	GSE13411_NAIVE_VS_SWITCHED_MEMORY_BCELL_UP
**44**	GSE13411_SWITCHED_MEMORY_BCELL_VS_PLASMA_CELL_DN
**45**	GSE13484_UNSTIM_VS_12H_YF17D_VACCINE_STIM_PBMC_UP
**46**	GSE13485_CTRL_VS_DAY1_YF17D_VACCINE_PBMC_DN
**47**	GSE13485_CTRL_VS_DAY21_YF17D_VACCINE_PBMC_DN
**48**	GSE13485_DAY1_VS_DAY3_YF17D_VACCINE_PBMC_UP
**49**	GSE13485_DAY1_VS_DAY7_YF17D_VACCINE_PBMC_UP
**50**	GSE13485_DAY3_VS_DAY21_YF17D_VACCINE_PBMC_DN
**51**	GSE13493_CD4INTCD8POS_VS_CD8POS_THYMOCYTE_DN
**52**	GSE13493_CD4INTCD8POS_VS_CD8POS_THYMOCYTE_UP
**53**	GSE13522_CTRL_VS_T_CRUZI_Y_STRAIN_INF_SKIN_129_MOUSE_UP
**54**	GSE13522_CTRL_VS_T_CRUZI_Y_STRAIN_INF_SKIN_IFNG_KO_DN
**55**	GSE13522_WT_VS_IFNG_KO_SKIN_DN
**56**	GSE13547_CTRL_VS_ANTI_IGM_STIM_BCELL_2H_DN
**57**	GSE13738_RESTING_VS_BYSTANDER_ACTIVATED_CD4_TCELL_UP
**58**	GSE13762_CTRL_VS_125_VITAMIND_DAY5_DC_DN
**59**	GSE13887_ACT_CD4_VS_NO_TREATED_CD4_TCELL_DN
**60**	GSE13887_HEALTHY_VS_LUPUS_RESTING_CD4_TCELL_DN
**61**	GSE13887_HEALTHY_VS_LUPUS_RESTING_CD4_TCELL_UP
**62**	GSE14000_TRANSLATED_RNA_VS_MRNA_16H_LPS_DC_UP
**63**	GSE14026_TH1_VS_TH17_UP
**64**	GSE14308_NAIVE_CD4_TCELL_VS_NATURAL_TREG_UP
**65**	GSE14308_TH2_VS_NATURAL_TREG_DN
**66**	GSE14308_TH2_VS_NATURAL_TREG_UP
**67**	GSE14308_TH2_VS_TH17_DN
**68**	GSE14350_TREG_VS_TEFF_DN
**69**	GSE14413_UNSTIM_VS_IFNB_STIM_L929_CELLS_DN
**70**	GSE14413_UNSTIM_VS_IFNB_STIM_RAW264_CELLS_UP
**71**	GSE14415_FOXP3_KO_NATURAL_TREG_VS_TCONV_UP
**72**	GSE1448_CTRL_VS_ANTI_VALPHA2_DP_THYMOCYTE_UP

**73**	GSE1460_CD4_THYMOCYTE_VS_NAIVE_CD4_TCELL_ADULT_BLOOD_DN
**74**	GSE1460_CD4_THYMOCYTE_VS_NAIVE_CD4_TCELL_CORD_BLOOD_DN
**75**	GSE1460_CORD_VS_ADULT_BLOOD_NAIVE_CD4_TCELL_UP
**76**	GSE1460_DP_THYMOCYTE_VS_NAIVE_CD4_TCELL_ADULT_BLOOD_DN
**77**	GSE1460_DP_THYMOCYTE_VS_NAIVE_CD4_TCELL_CORD_BLOOD_DN
**78**	GSE1460_DP_THYMOCYTE_VS_THYMIC_STROMAL_CELL_UP
**79**	GSE1460_INTRATHYMIC_T_PROGENITOR_VS_NAIVE_CD4_TCELL_CORD_BLOOD_DN
**80**	GSE1460_NAIVE_CD4_TCELL_CORD_BLOOD_VS_THYMIC_STROMAL_CELL_UP
**81**	GSE14769_20MIN_VS_360MIN_LPS_BMDM_DN
**82**	GSE14769_UNSTIM_VS_20MIN_LPS_BMDM_UP
**83**	GSE14908_RESTING_VS_HDM_STIM_CD4_TCELL_NONATOPIC_PATIENT_UP
**84**	GSE15324_ELF4_KO_VS_WT_ACTIVATED_CD8_TCELL_DN
**85**	GSE15324_NAIVE_VS_ACTIVATED_CD8_TCELL_DN
**86**	GSE15324_NAIVE_VS_ACTIVATED_CD8_TCELL_UP
**87**	GSE15330_HSC_VS_GRANULOCYTE_MONOCYTE_PROGENITOR_IKAROS_KO_DN
**88**	GSE15330_LYMPHOID_MULTIPOTENT_VS_GRANULOCYTE_MONOCYTE_PROGENITOR_DN
**89**	GSE15330_LYMPHOID_MULTIPOTENT_VS_MEGAKARYOCYTE_ERYTHROID_PROGENITOR_DN
**90**	GSE15330_LYMPHOID_MULTIPOTENT_VS_PRO_BCELL_UP
**91**	GSE15330_MEGAKARYOCYTE_ERYTHROID_VS_GRANULOCYTE_MONOCYTE_PROGENITOR_DN
**92**	GSE15330_WT_VS_IKAROS_KO_MEGAKARYOCYTE_ERYTHROID_PROGENITOR_DN
**93**	GSE15624_3H_VS_6H_HALOFUGINONE_TREATED_CD4_TCELL_UP
**94**	GSE15659_CD45RA_NEG_CD4_TCELL_VS_ACTIVATED_TREG_UP
**95**	GSE15659_CD45RA_NEG_CD4_TCELL_VS_NONSUPPRESSIVE_TCELL_UP
**96**	GSE15659_NAIVE_CD4_TCELL_VS_NONSUPPRESSIVE_TCELL_UP
**97**	GSE15659_NAIVE_VS_PTPRC_NEG_CD4_TCELL_UP
**98**	GSE15659_NONSUPPRESSIVE_TCELL_VS_ACTIVATED_TREG_UP
**99**	GSE15659_TREG_VS_TCONV_DN
**100**	GSE1566_WT_VS_EZH2_KO_LN_TCELL_UP
**101**	GSE15750_WT_VS_TRAF6KO_DAY10_EFF_CD8_TCELL_DN
**102**	GSE15750_WT_VS_TRAF6KO_DAY6_EFF_CD8_TCELL_UP
**103**	GSE15930_NAIVE_VS_72H_IN_VITRO_STIM_CD8_TCELL_UP
**104**	GSE15930_STIM_VS_STIM_AND_IFNAB_24H_CD8_T_CELL_UP
**105**	GSE16266_LPS_VS_HEATSHOCK_AND_LPS_STIM_MEF_UP
**106**	GSE16385_IFNG_TNF_VS_IL4_STIM_MACROPHAGE_DN
**107**	GSE16385_IFNG_TNF_VS_ROSIGLITAZONE_STIM_MACROPHAGE_DN
**108**	GSE16385_IL4_VS_ROSIGLITAZONE_STIM_MACROPHAGE_DN
**109**	GSE16385_MONOCYTE_VS_12H_IFNG_TNF_TREATED_MACROPHAGE_DN
**110**	GSE16385_MONOCYTE_VS_MACROPHAGE_DN
**111**	GSE16385_MONOCYTE_VS_MACROPHAGE_UP
**112**	GSE16385_ROSIGLITAZONE_IL4_VS_IFNG_TNF_STIM_MACROPHAGE_DN
**113**	GSE16385_ROSIGLITAZONE_IL4_VS_ROSIGLITAZONE_ALONE_STIM_MACROPHAGE_UP
**114**	GSE16385_ROSIGLITAZONE_VS_UNTREATED_IFNG_TNF_STIM_MACROPHAGE_UP

**115**	GSE16385_UNTREATED_VS_12H_IL4_TREATED_MACROPHAGE_DN
**116**	GSE16385_UNTREATED_VS_12H_IL4_TREATED_MACROPHAGE_UP
**117**	GSE16386_IL4_VS_IL4_AND_ROSIGLITAZONE_STIM_MACROPHAGE_6H_DN
**118**	GSE16450_CTRL_VS_IFNA_12H_STIM_MATURE_NEURON_CELL_LINE_UP
**119**	GSE16450_IMMATURE_VS_MATURE_NEURON_CELL_LINE_12H_IFNA_STIM_UP
**120**	GSE17186_BLOOD_VS_CORD_BLOOD_CD21HIGH_TRANSITIONAL_BCELL_DN
**121**	GSE17301_CTRL_VS_48H_ACD3_ACD28_STIM_CD8_TCELL_UP
**122**	GSE17301_CTRL_VS_48H_IFNA2_STIM_CD8_TCELL_DN
**123**	GSE1740_UNSTIM_VS_IFNA_STIMULATED_MCSF_IFNG_DERIVED_MACROPHAGE_UP
**124**	GSE17580_TREG_VS_TEFF_DN
**125**	GSE17721_0.5H_VS_12H_CPG_BMDC_UP
**126**	GSE17721_0.5H_VS_24H_LPS_BMDC_DN
**127**	GSE17721_0.5H_VS_8H_POLYIC_BMDC_UP
**128**	GSE17721_CPG_VS_GARDIQUIMOD_0.5H_BMDC_UP
**129**	GSE17721_CPG_VS_GARDIQUIMOD_12H_BMDC_UP
**130**	GSE17721_CPG_VS_GARDIQUIMOD_16H_BMDC_UP
**131**	GSE17721_CPG_VS_GARDIQUIMOD_8H_BMDC_UP
**132**	GSE17721_CTRL_VS_CPG_24H_BMDC_UP
**133**	GSE17721_CTRL_VS_CPG_8H_BMDC_DN
**134**	GSE17721_CTRL_VS_GARDIQUIMOD_1H_BMDC_UP
**135**	GSE17721_CTRL_VS_GARDIQUIMOD_6H_BMDC_UP
**136**	GSE17721_CTRL_VS_PAM3CSK4_0.5H_BMDC_DN
**137**	GSE17721_CTRL_VS_PAM3CSK4_24H_BMDC_DN
**138**	GSE17721_CTRL_VS_POLYIC_12H_BMDC_DN
**139**	GSE17721_CTRL_VS_POLYIC_1H_BMDC_DN
**140**	GSE17721_CTRL_VS_POLYIC_8H_BMDC_DN
**141**	GSE17721_LPS_VS_CPG_0.5H_BMDC_DN
**142**	GSE17721_LPS_VS_CPG_0.5H_BMDC_UP
**143**	GSE17721_LPS_VS_CPG_2H_BMDC_DN
**144**	GSE17721_LPS_VS_CPG_6H_BMDC_DN
**145**	GSE17721_LPS_VS_GARDIQUIMOD_1H_BMDC_UP
**146**	GSE17721_LPS_VS_GARDIQUIMOD_4H_BMDC_UP
**147**	GSE17721_LPS_VS_PAM3CSK4_0.5H_BMDC_UP
**148**	GSE17721_LPS_VS_PAM3CSK4_4H_BMDC_UP
**149**	GSE17721_LPS_VS_POLYIC_0.5H_BMDC_UP
**150**	GSE17721_LPS_VS_POLYIC_1H_BMDC_DN
**151**	GSE17721_LPS_VS_POLYIC_1H_BMDC_UP
**152**	GSE17721_PAM3CSK4_VS_CPG_16H_BMDC_UP
**153**	GSE17721_PAM3CSK4_VS_CPG_4H_BMDC_DN
**154**	GSE17721_PAM3CSK4_VS_GADIQUIMOD_0.5H_BMDC_UP
**155**	GSE17721_POLYIC_VS_CPG_0.5H_BMDC_UP
**156**	GSE17721_POLYIC_VS_CPG_2H_BMDC_DN
**157**	GSE17721_POLYIC_VS_GARDIQUIMOD_16H_BMDC_UP

**158**	GSE17721_POLYIC_VS_PAM3CSK4_16H_BMDC_DN
**159**	GSE17974_0H_VS_1H_IN_VITRO_ACT_CD4_TCELL_UP
**160**	GSE17974_IL4_AND_ANTI_IL12_VS_UNTREATED_0.5H_ACT_CD4_TCELL_UP
**161**	GSE17974_IL4_AND_ANTI_IL12_VS_UNTREATED_1H_ACT_CD4_TCELL_UP
**162**	GSE17974_IL4_AND_ANTI_IL12_VS_UNTREATED_24H_ACT_CD4_TCELL_UP
**163**	GSE17974_IL4_AND_ANTI_IL12_VS_UNTREATED_2H_ACT_CD4_TCELL_DN
**164**	GSE17974_IL4_AND_ANTI_IL12_VS_UNTREATED_2H_ACT_CD4_TCELL_UP
**165**	GSE17974_IL4_AND_ANTI_IL12_VS_UNTREATED_4H_ACT_CD4_TCELL_DN
**166**	GSE18281_CORTEX_VS_MEDULLA_THYMUS_DN
**167**	GSE18281_CORTICAL_VS_MEDULLARY_THYMOCYTE_DN
**168**	GSE18281_PERIMEDULLARY_CORTICAL_REGION_VS_WHOLE_CORTEX_THYMUS_DN
**169**	GSE18281_PERIMEDULLARY_CORTICAL_REGION_VS_WHOLE_MEDULLA_THYMUS_UP
**170**	GSE18281_SUBCAPSULAR_CORTICAL_REGION_VS_WHOLE_CORTEX_THYMUS_DN
**171**	GSE18893_CTRL_VS_TNF_TREATED_TCONV_24H_DN
**172**	GSE18893_CTRL_VS_TNF_TREATED_TCONV_2H_UP
**173**	GSE18893_TCONV_VS_TREG_2H_TNF_STIM_UP
**174**	GSE19198_1H_VS_6H_IL21_TREATED_TCELL_UP
**175**	GSE19401_RETINOIC_ACID_VS_RETINOIC_ACID_AND_PAM2CSK4_STIM_FOLLICULAR_DC_UP
**176**	GSE19512_NAUTRAL_VS_INDUCED_TREG_UP
**177**	GSE19772_HCMV_INFL_VS_HCMV_INF_MONOCYTES_AND_PI3K_INHIBITION_DN
**178**	GSE19825_NAIVE_VS_IL2RAHIGH_DAY3_EFF_CD8_TCELL_UP
**179**	GSE19888_ADENOSINE_A3R_INH_PRETREAT_AND_ACT_BY_A3R_VS_TCELL_MEMBRANES_ACT_MAST_CELL_DN
**180**	GSE19888_CTRL_VS_A3R_ACTIVATION_MAST_CELL_DN
**181**	GSE19888_CTRL_VS_A3R_ACT_TREATED_MAST_CELL_PRETREATED_WITH_A3R_INH_UP
**182**	GSE19923_E2A_KO_VS_E2A_AND_HEB_KO_DP_THYMOCYTE_UP
**183**	GSE19923_WT_VS_E2A_KO_DP_THYMOCYTE_UP
**184**	GSE19941_LPS_VS_LPS_AND_IL10_STIM_IL10_KO_MACROPHAGE_DN
**185**	GSE19941_LPS_VS_LPS_AND_IL10_STIM_IL10_KO_NFKBP50_KO_MACROPHAGE_DN
**186**	GSE19941_UNSTIM_VS_LPS_AND_IL10_STIM_IL10_KO_NFKBP50_KO_MACROPHAGE_UP
**187**	GSE20366_CD103_POS_VS_CD103_KLRG1_DP_TREG_UP
**188**	GSE20366_EX_VIVO_VS_DEC205_CONVERSION_NAIVE_CD4_TCELL_UP
**189**	GSE20366_EX_VIVO_VS_HOMEOSTATIC_CONVERSION_NAIVE_CD4_TCELL_DN
**190**	GSE20366_TREG_VS_NAIVE_CD4_TCELL_DEC205_CONVERSION_DN
**191**	GSE20366_TREG_VS_NAIVE_CD4_TCELL_DN
**192**	GSE20366_TREG_VS_TCONV_DN
**193**	GSE20715_WT_VS_TLR4_KO_6H_OZONE_LUNG_DN
**194**	GSE21033_3H_VS_12H_POLYIC_STIM_DC_UP
**195**	GSE21063_CTRL_VS_ANTI_IGM_STIM_BCELL_16H_DN
**196**	GSE21063_CTRL_VS_ANTI_IGM_STIM_BCELL_8H_DN
**197**	GSE21063_WT_VS_NFATC1_KO_16H_ANTI_IGM_STIM_BCELL_DN
**198**	GSE21063_WT_VS_NFATC1_KO_BCELL_UP

**199**	GSE2128_C57BL6_VS_NOD_THYMOCYTE_MIMETOPE_NEGATIVE_SELECTION_UP
**200**	GSE21379_TFH_VS_NON_TFH_CD4_TCELL_DN
**201**	GSE21546_UNSTIM_VS_ANTI_CD3_STIM_ELK1_KO_DP_THYMOCYTES_DN
**202**	GSE21546_UNSTIM_VS_ANTI_CD3_STIM_SAP1A_KO_AND_ELK1_KO_DP_THYMOCYTES_DN
**203**	GSE21546_UNSTIM_VS_ANTI_CD3_STIM_SAP1A_KO_DP_THYMOCYTES_DN
**204**	GSE21546_WT_VS_ELK1_KO_DP_THYMOCYTES_DN
**205**	GSE21670_TGFB_VS_TGFB_AND_IL6_TREATED_STAT3_KO_CD4_TCELL_UP
**206**	GSE21670_UNTREATED_VS_IL6_TREATED_CD4_TCELL_DN
**207**	GSE21670_UNTREATED_VS_TGFB_TREATED_CD4_TCELL_UP
**208**	GSE21927_GMCSF_IL6_VS_GMCSF_GCSF_TREATED_BONE_MARROW_UP
**209**	GSE21927_SPLENIC_C26GM_TUMOROUS_VS_4T1_TUMOR_MONOCYTES_UP
**210**	GSE22025_UNTREATED_VS_PROGESTERONE_TREATED_CD4_TCELL_UP
**211**	GSE22033_UNTREATED_VS_MRL24_TREATED_MEF_UP
**212**	GSE22045_TREG_VS_TCONV_DN
**213**	GSE22045_TREG_VS_TCONV_UP
**214**	GSE22229_UNTREATED_VS_IMMUNOSUPP_THERAPY_RENAL_TRANSPLANT_PATIENT_PBMC_DN
**215**	GSE22342_CD11C_HIGH_VS_LOW_DECIDUAL_MACROPHAGES_DN
**216**	GSE22443_IL2_VS_IL12_TREATED_ACT_CD8_TCELL_UP
**217**	GSE22443_NAIVE_VS_ACT_AND_IL2_TREATED_CD8_TCELL_DN
**218**	GSE22443_NAIVE_VS_ACT_AND_IL2_TREATED_CD8_TCELL_UP
**219**	GSE22601_DOUBLE_NEGATIVE_VS_IMMATURE_CD4_SP_THYMOCYTE_DN
**220**	GSE22601_DOUBLE_POSITIVE_VS_CD4_SINGLE_POSITIVE_THYMOCYTE_UP
**221**	GSE22601_IMMATURE_CD4_SINGLE_POSITIVE_VS_CD4_SINGLE_POSITIVE_THYMOCYTE_UP
**222**	GSE22601_IMMATURE_CD4_SINGLE_POSITIVE_VS_DOUBLE_POSITIVE_THYMOCYTE_UP
**223**	GSE22611_MUTANT_NOD2_TRANSDUCED_VS_CTRL_HEK293T_STIMULATED_WITH_MDP_6H_DN
**224**	GSE22611_NOD2_VS_MUTANT_NOD2_TRANSDUCED_HEK293T_CELL_UP
**225**	GSE22886_CD8_VS_CD4_NAIVE_TCELL_DN
**226**	GSE22886_IL2_VS_IL15_STIM_NKCELL_UP
**227**	GSE22886_NAIVE_BCELL_VS_MONOCYTE_UP
**228**	GSE22886_NAIVE_CD4_TCELL_VS_48H_ACT_TH2_UP
**229**	GSE22886_NAIVE_CD4_TCELL_VS_MEMORY_TCELL_UP
**230**	GSE22886_NAIVE_CD4_TCELL_VS_NKCELL_UP
**231**	GSE22886_NAIVE_TCELL_VS_NKCELL_UP
**232**	GSE22886_NAIVE_VS_MEMORY_TCELL_UP
**233**	GSE22886_NEUTROPHIL_VS_DC_UP
**234**	GSE22886_NEUTROPHIL_VS_MONOCYTE_UP
**235**	GSE22886_TCELL_VS_BCELL_NAIVE_DN
**236**	GSE22935_UNSTIM_VS_12H_MBOVIS_BCG_STIM_MYD88_KO_MACROPHAGE_UP
**237**	GSE22935_UNSTIM_VS_48H_MBOVIS_BCG_STIM_MYD88_KO_MACROPHAGE_DN
**238**	GSE23321_CD8_STEM_CELL_MEMORY_VS_CENTRAL_MEMORY_CD8_TCELL_UP
**239**	GSE23502_BM_VS_COLON_TUMOR_HDC_KO_MYELOID_DERIVED_SUPPRESSOR_CELL_UP

**240**	GSE23502_WT_VS_HDC_KO_MYELOID_DERIVED_SUPPRESSOR_CELL_COLON_TUMOR_DN
**241**	GSE23505_IL6_IL1_IL23_VS_IL6_IL1_TGFB_TREATED_CD4_TCELL_UP
**242**	GSE23505_IL6_IL1_VS_IL6_IL1_TGFB_TREATED_CD4_TCELL_UP
**243**	GSE23925_LIGHT_ZONE_VS_DARK_ZONE_BCELL_DN
**244**	GSE2405_0H_VS_3H_A_PHAGOCYTOPHILUM_STIM_NEUTROPHIL_DN
**245**	GSE2405_0H_VS_6H_A_PHAGOCYTOPHILUM_STIM_NEUTROPHIL_DN
**246**	GSE2405_0H_VS_9H_A_PHAGOCYTOPHILUM_STIM_NEUTROPHIL_UP
**247**	GSE2405_HEAT_KILLED_LYSATE_VS_LIVE_A_PHAGOCYTOPHILUM_STIM_NEUTROPHIL_9H_DN
**248**	GSE2405_HEAT_KILLED_VS_LIVE_A_PHAGOCYTOPHILUM_STIM_NEUTROPHIL_24H_UP
**249**	GSE2405_S_AUREUS_VS_A_PHAGOCYTOPHILUM_NEUTROPHIL_UP
**250**	GSE24142_ADULT_VS_FETAL_DN3_THYMOCYTE_DN
**251**	GSE24142_ADULT_VS_FETAL_EARLY_THYMIC_PROGENITOR_DN
**252**	GSE24210_IL35_TREATED_VS_RESTING_TREG_DN
**253**	GSE24210_IL35_TREATED_VS_RESTING_TREG_UP
**254**	GSE24210_IL35_TREATED_VS_UNTREATED_TCONV_CD4_TCELL_UP
**255**	GSE24210_TCONV_VS_TREG_UP
**256**	GSE24574_BCL6_HIGH_TFH_VS_TCONV_CD4_TCELL_DN
**257**	GSE24574_BCL6_HIGH_TFH_VS_TCONV_CD4_TCELL_UP
**258**	GSE24574_BCL6_LOW_TFH_VS_TCONV_CD4_TCELL_UP
**259**	GSE24634_NAIVE_CD4_TCELL_VS_DAY10_IL4_CONV_TREG_UP
**260**	GSE24634_NAIVE_CD4_TCELL_VS_DAY3_IL4_CONV_TREG_UP
**261**	GSE24634_TEFF_VS_TCONV_DAY5_IN_CULTURE_DN
**262**	GSE24814_STAT5_KO_VS_WT_PRE_BCELL_UP
**263**	GSE24972_MARGINAL_ZONE_BCELL_VS_FOLLICULAR_BCELL_IRF8_KO_UP
**264**	GSE24972_MARGINAL_ZONE_BCELL_VS_FOLLICULAR_BCELL_UP
**265**	GSE24972_WT_VS_IRF8_KO_SPLEEN_FOLLICULAR_BCELL_UP
**266**	GSE25087_TREG_VS_TCONV_ADULT_DN
**267**	GSE25088_CTRL_VS_IL4_AND_ROSIGLITAZONE_STIM_MACROPHAGE_UP
**268**	GSE25088_ROSIGLITAZONE_VS_IL4_AND_ROSIGLITAZONE_STIM_MACROPHAGE_DAY10_DN
**269**	GSE25088_WT_VS_STAT6_KO_MACROPHAGE_UP
**270**	GSE25123_CTRL_VS_IL4_STIM_PPARG_KO_MACROPHAGE_DN
**271**	GSE25123_IL4_VS_IL4_AND_ROSIGLITAZONE_STIM_MACROPHAGE_DAY10_UP
**272**	GSE25123_WT_VS_PPARG_KO_MACROPHAGE_IL4_AND_ROSIGLITAZONE_STIM_UP
**273**	GSE25146_UNSTIM_VS_HELIOBACTER_PYLORI_LPS_STIM_AGS_CELL_DN
**274**	GSE25147_UNSTIM_VS_HELIOBACTER_PYLORI_LPS_STIM_MKN45_CELL_DN
**275**	GSE2585_AIRE_KO_VS_WT_CD80_HIGH_MTEC_UP
**276**	GSE2585_THYMIC_MACROPHAGE_VS_MTEC_DN
**277**	GSE26023_PHD3_KO_VS_WT_NEUTROPHIL_HYPOXIA_UP
**278**	GSE26343_UNSTIM_VS_LPS_STIM_MACROPHAGE_UP
**279**	GSE26488_WT_VS_VP16_TRANSGENIC_HDAC7_KO_DOUBLE_POSITIVE_THYMOCYTE_UP
**280**	GSE26495_NAIVE_VS_PD1HIGH_CD8_TCELL_UP
**281**	GSE26495_NAIVE_VS_PD1LOW_CD8_TCELL_UP

**282**	GSE26495_PD1HIGH_VS_PD1LOW_CD8_TCELL_UP
**283**	GSE26559_TCF1_KO_VS_WT_LIN_NEG_CELL_DN
**284**	GSE26669_CD4_VS_CD8_TCELL_IN_MLR_COSTIM_BLOCK_UP
**285**	GSE26669_CTRL_VS_COSTIM_BLOCK_MLR_CD4_TCELL_DN
**286**	GSE26928_EFF_MEMORY_VS_CXCR5_POS_CD4_TCELL_UP
**287**	GSE26928_EFF_MEM_VS_CENTR_MEM_CD4_TCELL_DN
**288**	GSE26928_EFF_MEM_VS_CENTR_MEM_CD4_TCELL_UP
**289**	GSE2706_R848_VS_LPS_2H_STIM_DC_UP
**290**	GSE2706_R848_VS_LPS_8H_STIM_DC_UP
**291**	GSE2770_IL12_ACT_VS_ACT_CD4_TCELL_6H_DN
**292**	GSE2770_IL12_AND_TGFB_ACT_VS_ACT_CD4_TCELL_6H_UP
**293**	GSE2770_IL12_AND_TGFB_VS_IL4_TREATED_ACT_CD4_TCELL_6H_DN
**294**	GSE2770_TGFB_AND_IL4_VS_IL4_TREATED_ACT_CD4_TCELL_6H_UP
**295**	GSE2770_TGFB_AND_IL4_VS_TGFB_AND_IL12_TREATED_ACT_CD4_TCELL_2H_UP
**296**	GSE2770_TGFB_AND_IL4_VS_TGFB_AND_IL12_TREATED_ACT_CD4_TCELL_6H_UP
**297**	GSE2770_UNTREATED_VS_TGFB_AND_IL12_TREATED_ACT_CD4_TCELL_2H_UP
**298**	GSE2770_UNTREATED_VS_TGFB_AND_IL12_TREATED_ACT_CD4_TCELL_6H_DN
**299**	GSE2770_UNTREATED_VS_TGFB_AND_IL4_TREATED_ACT_CD4_TCELL_48H_DN
**300**	GSE27786_BCELL_VS_ERYTHROBLAST_DN
**301**	GSE27786_BCELL_VS_MONO_MAC_DN
**302**	GSE27786_BCELL_VS_NEUTROPHIL_DN
**303**	GSE27786_CD4_TCELL_VS_ERYTHTROBLAST_DN
**304**	GSE27786_CD4_TCELL_VS_NEUTROPHIL_DN
**305**	GSE27786_CD4_VS_CD8_TCELL_DN
**306**	GSE27786_LIN_NEG_VS_ERYTHROBLAST_DN
**307**	GSE27786_LIN_NEG_VS_MONO_MAC_DN
**308**	GSE27786_LSK_VS_NEUTROPHIL_DN
**309**	GSE27786_LSK_VS_NKTCELL_DN
**310**	GSE27786_NKCELL_VS_NEUTROPHIL_DN
**311**	GSE27786_NKTCELL_VS_NEUTROPHIL_UP
**312**	GSE28130_ACTIVATED_VS_INDUCEED_TREG_DN
**313**	GSE28237_EARLY_VS_LATE_GC_BCELL_DN
**314**	GSE2826_WT_VS_XID_BCELL_DN
**315**	GSE28449_WT_VS_LRF_KO_GERMINAL_CENTER_BCELL_UP
**316**	GSE28737_BCL6_HET_VS_BCL6_KO_MARGINAL_ZONE_BCELL_UP
**317**	GSE28737_FOLLICULAR_VS_MARGINAL_ZONE_BCELL_BCL6_HET_DN
**318**	GSE28737_WT_VS_BCL6_HET_FOLLICULAR_BCELL_UP
**319**	GSE28737_WT_VS_BCL6_KO_FOLLICULAR_BCELL_UP
**320**	GSE28783_ANTI_MIR33_VS_CTRL_ATHEROSCLEROSIS_MACROPHAGE_UP
**321**	GSE29164_CD8_TCELL_VS_CD8_TCELL_AND_IL12_TREATED_MELANOMA_DAY7_DN
**322**	GSE29164_DAY3_VS_DAY7_CD8_TCELL_AND_IL12_TREATED_MELANOMA_DN
**323**	GSE29164_DAY3_VS_DAY7_CD8_TCELL_TREATED_MELANOMA_UP
**324**	GSE29164_UNTREATED_VS_CD8_TCELL_TREATED_MELANOMA_DAY3_DN

**325**	GSE29614_CTRL_VS_DAY7_TIV_FLU_VACCINE_PBMC_UP
**326**	GSE29614_DAY3_VS_DAY7_TIV_FLU_VACCINE_PBMC_DN
**327**	GSE29615_CTRL_VS_DAY3_LAIV_IFLU_VACCINE_PBMC_DN
**328**	GSE29615_CTRL_VS_DAY7_LAIV_FLU_VACCINE_PBMC_DN
**329**	GSE29615_DAY3_VS_DAY7_LAIV_FLU_VACCINE_PBMC_UP
**330**	GSE29617_CTRL_VS_DAY7_TIV_FLU_VACCINE_PBMC_2008_DN
**331**	GSE29618_LAIV_VS_TIV_FLU_VACCINE_DAY7_MONOCYTE_DN
**332**	GSE29618_PDC_VS_MDC_DAY7_FLU_VACCINE_UP
**333**	GSE29618_PRE_VS_DAY7_FLU_VACCINE_MDC_DN
**334**	GSE29618_PRE_VS_DAY7_FLU_VACCINE_MDC_UP
**335**	GSE29618_PRE_VS_DAY7_FLU_VACCINE_PDC_UP
**336**	GSE29618_PRE_VS_DAY7_POST_LAIV_FLU_VACCINE_BCELL_DN
**337**	GSE29618_PRE_VS_DAY7_POST_LAIV_FLU_VACCINE_PDC_UP
**338**	GSE29618_PRE_VS_DAY7_POST_TIV_FLU_VACCINE_MDC_DN
**339**	GSE29618_PRE_VS_DAY7_POST_TIV_FLU_VACCINE_MDC_UP
**340**	GSE29618_PRE_VS_DAY7_POST_TIV_FLU_VACCINE_MONOCYTE_DN
**341**	GSE29949_CD8_NEG_DC_SPLEEN_VS_MONOCYTE_BONE_MARROW_DN
**342**	GSE29949_DC_BRAIN_VS_MONOCYTE_BONE_MARROW_DN
**343**	GSE29949_MICROGLIA_BRAIN_VS_CD8_NEG_DC_SPLEEN_UP
**344**	GSE29949_MICROGLIA_BRAIN_VS_CD8_POS_DC_SPLEEN_DN
**345**	GSE29949_MICROGLIA_VS_DC_BRAIN_DN
**346**	GSE30083_SP1_VS_SP2_THYMOCYTE_DN
**347**	GSE30083_SP2_VS_SP3_THYMOCYTE_DN
**348**	GSE30083_SP2_VS_SP3_THYMOCYTE_UP
**349**	GSE30083_SP2_VS_SP4_THYMOCYTE_UP
**350**	GSE30153_LUPUS_VS_HEALTHY_DONOR_BCELL_DN
**351**	GSE3039_ALPHAALPHA_CD8_TCELL_VS_B1_BCELL_DN
**352**	GSE3039_ALPHAALPHA_VS_ALPHABETA_CD8_TCELL_DN
**353**	GSE3039_CD4_TCELL_VS_B2_BCELL_UP
**354**	GSE3039_NKT_CELL_VS_B1_BCELL_DN
**355**	GSE30971_2H_VS_4H_LPS_STIM_MACROPHAGE_WBP7_HET_UP
**356**	GSE31622_WT_VS_KLF3_KO_BCELL_DN
**357**	GSE32034_UNTREATED_VS_ROSIGLIZATONE_TREATED_LY6C_LOW_MONOCYTE_DN
**358**	GSE3203_HEALTHY_VS_INFLUENZA_INFECTED_LN_BCELL_DN
**359**	GSE3203_INFLUENZA_INF_VS_IFNB_TREATED_LN_BCELL_UP
**360**	GSE3203_WT_VS_IFNAR1_KO_INFLUENZA_INFECTED_LN_BCELL_DN
**361**	GSE3203_WT_VS_IFNAR1_KO_INFLUENZA_INFECTED_LN_BCELL_UP
**362**	GSE32423_CTRL_VS_IL7_IL4_MEMORY_CD8_TCELL_DN
**363**	GSE32423_CTRL_VS_IL7_MEMORY_CD8_TCELL_DN
**364**	GSE32423_CTRL_VS_IL7_MEMORY_CD8_TCELL_UP
**365**	GSE32423_IL7_VS_IL7_IL4_NAIVE_CD8_TCELL_UP
**366**	GSE32423_MEMORY_VS_NAIVE_CD8_TCELL_IL7_IL4_UP
**367**	GSE32423_MEMORY_VS_NAIVE_CD8_TCELL_IL7_UP

**368**	GSE32533_MIR17_KO_VS_MIR17_OVEREXPRESS_ACT_CD4_TCELL_DN
**369**	GSE32901_NAIVE_VS_TH17_NEG_CD4_TCELL_DN
**370**	GSE32901_NAIVE_VS_TH1_CD4_TCELL_UP
**371**	GSE33162_HDAC3_KO_VS_HDAC3_KO_4H_LPS_STIM_MACROPHAGE_DN
**372**	GSE33162_UNTREATED_VS_4H_LPS_STIM_HDAC3_HET_MACROPHAGE_DN
**373**	GSE33292_DN3_THYMOCYTE_VS_TCELL_LYMPHOMA_FROM_TCF1_KO_DN
**374**	GSE33292_WT_VS_TCF1_KO_DN3_THYMOCYTE_UP
**375**	GSE33374_CD8_ALPHAALPHA_VS_ALPHABETA_CD161_HIGH_TCELL_UP
**376**	GSE33425_CD161_HIGH_VS_INT_CD8_TCELL_DN
**377**	GSE33513_TCF7_KO_VS_HET_EARLY_THYMIC_PROGENITOR_DN
**378**	GSE339_CD4POS_VS_CD4CD8DN_DC_DN
**379**	GSE34156_NOD2_LIGAND_VS_NOD2_AND_TLR1_TLR2_LIGAND_24H_TREATED_MONOCYTE_DN
**380**	GSE34156_NOD2_LIGAND_VS_TLR1_TLR2_LIGAND_24H_TREATED_MONOCYTE_DN
**381**	GSE34156_NOD2_LIGAND_VS_TLR1_TLR2_LIGAND_24H_TREATED_MONOCYTE_UP
**382**	GSE34156_TLR1_TLR2_LIGAND_VS_NOD2_AND_TLR1_TLR2_LIGAND_6H_TREATED_MONOCYTE_DN
**383**	GSE34156_UNTREATED_VS_24H_NOD2_AND_TLR1_TLR2_LIGAND_TREATED_MONOCYTE_DN
**384**	GSE34156_UNTREATED_VS_24H_NOD2_LIGAND_TREATED_MONOCYTE_UP
**385**	GSE34156_UNTREATED_VS_24H_TLR1_TLR2_LIGAND_TREATED_MONOCYTE_DN
**386**	GSE34205_HEALTHY_VS_RSV_INF_INFANT_PBMC_UP
**387**	GSE34205_RSV_VS_FLU_INF_INFANT_PBMC_UP
**388**	GSE34217_MIR17_92_OVEREXPRESS_VS_WT_ACT_CD8_TCELL_DN
**389**	GSE34217_MIR17_92_OVEREXPRESS_VS_WT_ACT_CD8_TCELL_UP
**390**	GSE35543_IN_VITRO_ITREG_VS_CONVERTED_EX_ITREG_DN
**391**	GSE35543_IN_VIVO_NTREG_VS_IN_VITRO_ITREG_UP
**392**	GSE35685_CD34POS_CD38NEG_VS_CD34POS_CD10NEG_CD62LPOS_BONE_MARROW_DN
**393**	GSE35825_IFNA_VS_IFNG_STIM_MACROPHAGE_DN
**394**	GSE36009_UNSTIM_VS_LPS_STIM_NLRP10_KO_DC_DN
**395**	GSE36078_UNTREATED_VS_AD5_INF_MOUSE_LUNG_DC_UP
**396**	GSE36078_UNTREATED_VS_AD5_T425A_HEXON_INF_IL1R_KO_MOUSE_LUNG_DC_UP
**397**	GSE36078_WT_VS_IL1R_KO_LUNG_DC_AFTER_AD5_INF_UP
**398**	GSE36078_WT_VS_IL1R_KO_LUNG_DC_DN
**399**	GSE36078_WT_VS_IL1R_KO_LUNG_DC_UP
**400**	GSE360_CTRL_VS_B_MALAYI_LOW_DOSE_DC_UP
**401**	GSE360_HIGH_VS_LOW_DOSE_B_MALAYI_DC_UP
**402**	GSE360_LOW_DOSE_B_MALAYI_VS_M_TUBERCULOSIS_MAC_UP
**403**	GSE360_L_DONOVANI_VS_L_MAJOR_MAC_DN
**404**	GSE360_L_DONOVANI_VS_T_GONDII_DC_DN
**405**	GSE360_L_MAJOR_VS_B_MALAYI_HIGH_DOSE_DC_DN
**406**	GSE360_L_MAJOR_VS_B_MALAYI_LOW_DOSE_MAC_UP
**407**	GSE360_L_MAJOR_VS_M_TUBERCULOSIS_DC_DN

**408**	GSE360_L_MAJOR_VS_M_TUBERCULOSIS_MAC_DN
**409**	GSE360_L_MAJOR_VS_M_TUBERCULOSIS_MAC_UP
**410**	GSE36392_EOSINOPHIL_VS_NEUTROPHIL_IL25_TREATED_LUNG_DN
**411**	GSE36392_TYPE_2_MYELOID_VS_EOSINOPHIL_IL25_TREATED_LUNG_UP
**412**	GSE36392_TYPE_2_MYELOID_VS_MAC_IL25_TREATED_LUNG_UP
**413**	GSE36392_TYPE_2_MYELOID_VS_NEUTROPHIL_IL25_TREATED_LUNG_DN
**414**	GSE36476_CTRL_VS_TSST_ACT_72H_MEMORY_CD4_TCELL_OLD_UP
**415**	GSE36476_YOUNG_VS_OLD_DONOR_MEMORY_CD4_TCELL_40H_TSST_ACT_UP
**416**	GSE36476_YOUNG_VS_OLD_DONOR_MEMORY_CD4_TCELL_72H_TSST_ACT_UP
**417**	GSE36527_CD62L_HIGH_CD69_NEG_VS_CD62L_LOW_CD69_POS_TREG_KLRG1_NEG_UP
**418**	GSE36888_UNTREATED_VS_IL2_TREATED_STAT5_AB_KNOCKIN_TCELL_17H_DN
**419**	GSE36888_UNTREATED_VS_IL2_TREATED_STAT5_AB_KNOCKIN_TCELL_2H_DN
**420**	GSE3691_CONVENTIONAL_VS_PLASMACYTOID_DC_SPLEEN_DN
**421**	GSE3691_CONVENTIONAL_VS_PLASMACYTOID_DC_SPLEEN_UP
**422**	GSE3691_IFN_PRODUCING_KILLER_DC_VS_CONVENTIONAL_DC_SPLEEN_DN
**423**	GSE3691_IFN_PRODUCING_KILLER_DC_VS_PLASMACYTOID_DC_SPLEEN_DN
**424**	GSE369_PRE_VS_POST_IL6_INJECTION_IFNG_KO_LIVER_DN
**425**	GSE369_PRE_VS_POST_IL6_INJECTION_IFNG_KO_LIVER_UP
**426**	GSE3720_LPS_VS_PMA_STIM_VD1_GAMMADELTA_TCELL_DN
**427**	GSE3720_LPS_VS_PMA_STIM_VD2_GAMMADELTA_TCELL_DN
**428**	GSE3720_VD1_VS_VD2_GAMMADELTA_TCELL_WITH_LPS_STIM_DN
**429**	GSE37301_COMMON_LYMPHOID_PROGENITOR_VS_GRAN_MONO_PROGENITOR_DN
**430**	GSE37301_COMMON_LYMPHOID_PROGENITOR_VS_RAG2_KO_NK_CELL_DN
**431**	GSE37301_HEMATOPOIETIC_STEM_CELL_VS_MULTIPOTENT_PROGENITOR_UP
**432**	GSE37301_LYMPHOID_PRIMED_MPP_VS_COMMON_LYMPHOID_PROGENITOR_UP
**433**	GSE37301_LYMPHOID_PRIMED_MPP_VS_RAG2_KO_NK_CELL_DN
**434**	GSE37301_MULTIPOTENT_PROGENITOR_VS_GRAN_MONO_PROGENITOR_UP
**435**	GSE37336_LY6C_POS_VS_NEG_NAIVE_CD4_TCELL_DN
**436**	GSE37336_LY6C_POS_VS_NEG_NAIVE_CD4_TCELL_UP
**437**	GSE37416_0H_VS_12H_F_TULARENSIS_LVS_NEUTROPHIL_UP
**438**	GSE37416_12H_VS_48H_F_TULARENSIS_LVS_NEUTROPHIL_DN
**439**	GSE37416_CTRL_VS_0H_F_TULARENSIS_LVS_NEUTROPHIL_DN
**440**	GSE37416_CTRL_VS_0H_F_TULARENSIS_LVS_NEUTROPHIL_UP
**441**	GSE37416_CTRL_VS_3H_F_TULARENSIS_LVS_NEUTROPHIL_UP
**442**	GSE37416_CTRL_VS_6H_F_TULARENSIS_LVS_NEUTROPHIL_UP
**443**	GSE37532_WT_VS_PPARG_KO_LN_TREG_DN
**444**	GSE37532_WT_VS_PPARG_KO_LN_TREG_UP
**445**	GSE37533_PPARG2_FOXP3_VS_FOXP3_TRANSDUCED_CD4_TCELL_PIOGLITAZONE_TREATED_UP
**446**	GSE37533_UNTREATED_VS_PIOGLIZATONE_TREATED_CD4_TCELL_PPARG2_AND_FOXP3_TRASDUCED_UP
**447**	GSE37534_GW1929_VS_PIOGLITAZONE_TREATED_CD4_TCELL_PPARG1_FOXP3_TRANSDUCED_UP

**448**	GSE37534_GW1929_VS_ROSIGLITAZONE_TREATED_CD4_TCELL_PPARG1_FOXP3_TRANSDUCED_DN
**449**	GSE37534_UNTREATED_VS_ROSIGLITAZONE_TREATED_CD4_TCELL_PPARG1_AND_FOXP3_TRASDUCED_UP
**450**	GSE37605_C57BL6_VS_NOD_FOXP3_IRES_GFP_TREG_UP
**451**	GSE37605_TREG_VS_TCONV_C57BL6_FOXP3_FUSION_GFP_UP
**452**	GSE37605_TREG_VS_TCONV_C57BL6_FOXP3_IRES_GFP_UP
**453**	GSE38304_MYC_NEG_VS_POS_GC_BCELL_UP
**454**	GSE39110_UNTREATED_VS_IL2_TREATED_CD8_TCELL_DAY6_POST_IMMUNIZATION_UP
**455**	GSE3920_UNTREATED_VS_IFNG_TREATED_FIBROBLAST_UP
**456**	GSE39820_IL1B_IL6_VS_IL1B_IL6_IL23A_TREATED_CD4_TCELL_UP
**457**	GSE39820_TGFBETA1_VS_TGFBETA3_IN_IL6_IL23A_TREATED_CD4_TCELL_DN
**458**	GSE39820_TGFBETA1_VS_TGFBETA3_IN_IL6_IL23A_TREATED_CD4_TCELL_UP
**459**	GSE39820_TGFBETA1_VS_TGFBETA3_IN_IL6_TREATED_CD4_TCELL_DN
**460**	GSE3982_BASOPHIL_VS_CENT_MEMORY_CD4_TCELL_DN
**461**	GSE3982_BASOPHIL_VS_EFF_MEMORY_CD4_TCELL_DN
**462**	GSE3982_BASOPHIL_VS_NKCELL_DN
**463**	GSE3982_BCELL_VS_BASOPHIL_DN
**464**	GSE3982_BCELL_VS_CENT_MEMORY_CD4_TCELL_DN
**465**	GSE3982_BCELL_VS_NKCELL_UP
**466**	GSE3982_DC_VS_BCELL_DN
**467**	GSE3982_DC_VS_CENT_MEMORY_CD4_TCELL_DN
**468**	GSE3982_DC_VS_EFF_MEMORY_CD4_TCELL_DN
**469**	GSE3982_DC_VS_MAC_DN
**470**	GSE3982_DC_VS_NEUTROPHIL_DN
**471**	GSE3982_DC_VS_NEUTROPHIL_UP
**472**	GSE3982_EFF_MEMORY_CD4_TCELL_VS_NKCELL_UP
**473**	GSE3982_EFF_MEMORY_VS_CENT_MEMORY_CD4_TCELL_DN
**474**	GSE3982_EOSINOPHIL_VS_CENT_MEMORY_CD4_TCELL_DN
**475**	GSE3982_EOSINOPHIL_VS_EFF_MEMORY_CD4_TCELL_DN
**476**	GSE3982_EOSINOPHIL_VS_MAST_CELL_UP
**477**	GSE3982_EOSINOPHIL_VS_NEUTROPHIL_DN
**478**	GSE3982_EOSINOPHIL_VS_NKCELL_DN
**479**	GSE3982_MAC_VS_CENT_MEMORY_CD4_TCELL_DN
**480**	GSE3982_MAC_VS_NKCELL_DN
**481**	GSE3982_MAST_CELL_VS_BCELL_DN
**482**	GSE3982_MAST_CELL_VS_CENT_MEMORY_CD4_TCELL_DN
**483**	GSE3982_MAST_CELL_VS_EFF_MEMORY_CD4_TCELL_DN
**484**	GSE3982_MAST_CELL_VS_NKCELL_DN
**485**	GSE3982_NEUTROPHIL_VS_EFF_MEMORY_CD4_TCELL_DN
**486**	GSE3994_WT_VS_PAC1_KO_ACTIVATED_MAST_CELL_UP
**487**	GSE40273_XBP1_KO_VS_WT_TREG_DN
**488**	GSE40274_CTRL_VS_FOXP3_AND_GATA1_TRANSDUCED_ACTIVATED_CD4_TCELL_UP

**489**	GSE40274_CTRL_VS_FOXP3_AND_PBX1_TRANSDUCED_ACTIVATED_CD4_TCELL_UP
**490**	GSE40274_CTRL_VS_FOXP3_AND_SATB1_TRANSDUCED_ACTIVATED_CD4_TCELL_DN
**491**	GSE40274_FOXP3_VS_FOXP3_AND_GATA1_TRANSDUCED_ACTIVATED_CD4_TCELL_UP
**492**	GSE40274_FOXP3_VS_FOXP3_AND_IRF4_TRANSDUCED_ACTIVATED_CD4_TCELL_DN
**493**	GSE40274_FOXP3_VS_FOXP3_AND_PBX1_TRANSDUCED_ACTIVATED_CD4_TCELL_UP
**494**	GSE40277_EOS_AND_LEF1_TRANSDUCED_VS_CTRL_CD4_TCELL_DN
**495**	GSE40441_NRP1_POS_INDUCED_TREG_VS_NRP1_NEG_NATURAL_TREG_UP
**496**	GSE40493_BCL6_KO_VS_WT_TREG_DN
**497**	GSE40655_FOXO1_KO_VS_WT_NTREG_DN
**498**	GSE40666_UNTREATED_VS_IFNA_STIM_STAT1_KO_CD8_TCELL_90MIN_UP
**499**	GSE41176_UNSTIM_VS_ANTI_IGM_STIM_TAK1_KO_BCELL_3H_DN
**500**	GSE41176_UNSTIM_VS_ANTI_IGM_STIM_TAK1_KO_BCELL_3H_UP
**501**	GSE41176_UNSTIM_VS_ANTI_IGM_STIM_TAK1_KO_BCELL_6H_DN
**502**	GSE41176_WT_VS_TAK1_KO_ANTI_IGM_STIM_BCELL_1H_UP
**503**	GSE411_WT_VS_SOCS3_KO_MACROPHAGE_IL6_STIM_400MIN_UP
**504**	GSE4142_NAIVE_BCELL_VS_PLASMA_CELL_DN
**505**	GSE4142_PLASMA_CELL_VS_GC_BCELL_UP
**506**	GSE41867_DAY6_VS_DAY15_LCMV_ARMSTRONG_EFFECTOR_CD8_TCELL_UP
**507**	GSE41867_DAY8_VS_DAY15_LCMV_ARMSTRONG_EFFECTOR_CD8_TCELL_UP
**508**	GSE41867_NAIVE_VS_DAY30_LCMV_CLONE13_EXHAUSTED_CD8_TCELL_UP
**509**	GSE41867_NAIVE_VS_DAY6_LCMV_ARMSTRONG_EFFECTOR_CD8_TCELL_DN
**510**	GSE41978_WT_VS_BIM_KO_KLRG1_LOW_EFFECTOR_CD8_TCELL_UP
**511**	GSE41978_WT_VS_ID2_KO_AND_BIM_KO_KLRG1_LOW_EFFECTOR_CD8_TCELL_DN
**512**	GSE42724_B1_BCELL_VS_PLASMABLAST_UP
**513**	GSE43863_TFH_VS_LY6C_INT_CXCR5POS_EFFECTOR_CD4_TCELL_DN
**514**	GSE43863_TH1_VS_TFH_EFFECTOR_CD4_TCELL_UP
**515**	GSE43955_10H_VS_60H_ACT_CD4_TCELL_DN
**516**	GSE43955_1H_VS_42H_ACT_CD4_TCELL_DN
**517**	GSE43955_1H_VS_42H_ACT_CD4_TCELL_WITH_TGFB_IL6_DN
**518**	GSE43955_TH0_VS_TGFB_IL6_TH17_ACT_CD4_TCELL_10H_DN
**519**	GSE43955_TH0_VS_TGFB_IL6_TH17_ACT_CD4_TCELL_60H_DN
**520**	GSE43956_WT_VS_SGK1_KO_TH17_DIFFERENTIATED_CD4_TCELL_UP
**521**	GSE44649_WT_VS_MIR155_KO_ACTIVATED_CD8_TCELL_DN
**522**	GSE44649_WT_VS_MIR155_KO_NAIVE_CD8_TCELL_UP
**523**	GSE45365_CD8A_DC_VS_CD11B_DC_IFNAR_KO_MCMV_INFECTION_DN
**524**	GSE45365_CD8A_DC_VS_CD11B_DC_IFNAR_KO_MCMV_INFECTION_UP
**525**	GSE45365_HEALTHY_VS_MCMV_INFECTION_CD11B_DC_IFNAR_KO_UP
**526**	GSE45365_HEALTHY_VS_MCMV_INFECTION_CD8A_DC_IFNAR_KO_UP
**527**	GSE45365_HEALTHY_VS_MCMV_INFECTION_CD8A_DC_UP
**528**	GSE45365_NK_CELL_VS_CD11B_DC_UP
**529**	GSE45365_NK_CELL_VS_CD8A_DC_DN
**530**	GSE45365_NK_CELL_VS_CD8A_DC_MCMV_INFECTION_DN
**531**	GSE45365_NK_CELL_VS_CD8A_DC_MCMV_INFECTION_UP

**532**	GSE45365_WT_VS_IFNAR_KO_CD8A_DC_MCMV_INFECTION_DN
**533**	GSE45739_UNSTIM_VS_ACD3_ACD28_STIM_WT_CD4_TCELL_UP
**534**	GSE45881_CXCR6HI_VS_CXCR1LO_COLONIC_LAMINA_PROPRIA_DN
**535**	GSE4590_PRE_BCELL_VS_SMALL_PRE_BCELL_UP
**536**	GSE4590_SMALL_VS_VPREB_POS_LARGE_PRE_BCELL_DN
**537**	GSE46242_CTRL_VS_EGR2_DELETED_TH1_CD4_TCELL_DN
**538**	GSE46242_TH1_VS_ANERGIC_TH1_CD4_TCELL_WITH_EGR2_DELETED_DN
**539**	GSE46606_DAY1_VS_DAY3_CD40L_IL2_IL5_STIMULATED_BCELL_UP
**540**	GSE46606_DAY1_VS_DAY3_CD40L_IL2_IL5_STIMULATED_IRF4HIGH_BCELL_DN
**541**	GSE46606_DAY1_VS_DAY3_CD40L_IL2_IL5_STIMULATED_IRF4_KO_BCELL_UP
**542**	GSE46606_IRF4HIGH_VS_IRF4MID_CD40L_IL2_IL5_DAY1_STIMULATED_BCELL_DN
**543**	GSE46606_IRF4_KO_VS_WT_CD40L_IL2_IL5_1DAY_STIMULATED_BCELL_DN
**544**	GSE46606_UNSTIM_VS_CD40L_IL2_IL5_1DAY_STIMULATED_IRF4HIGH_SORTED_BCELL_UP
**545**	GSE46606_UNSTIM_VS_CD40L_IL2_IL5_1DAY_STIMULATED_IRF4MID_SORTED_BCELL_DN
**546**	GSE46606_UNSTIM_VS_CD40L_IL2_IL5_1DAY_STIMULATED_IRF4_KO_BCELL_DN
**547**	GSE46606_UNSTIM_VS_CD40L_IL2_IL5_3DAY_STIMULATED_IRF4HIGH_SORTED_BCELL_DN
**548**	GSE4748_LPS_VS_LPS_AND_CYANOBACTERIUM_LPSLIKE_STIM_DC_3H_DN
**549**	GSE4984_GALECTIN1_VS_LPS_STIM_DC_DN
**550**	GSE4984_LPS_VS_VEHICLE_CTRL_TREATED_DC_UP
**551**	GSE4984_UNTREATED_VS_GALECTIN1_TREATED_DC_DN
**552**	GSE5099_MONOCYTE_VS_CLASSICAL_M1_MACROPHAGE_DN
**553**	GSE5099_UNSTIM_VS_MCSF_TREATED_MONOCYTE_DAY3_DN
**554**	GSE5503_LIVER_DC_VS_PLN_DC_ACTIVATED_ALLOGENIC_TCELL_UP
**555**	GSE5503_LIVER_DC_VS_SPLEEN_DC_ACTIVATED_ALLOGENIC_TCELL_DN
**556**	GSE5542_IFNA_VS_IFNA_AND_IFNG_TREATED_EPITHELIAL_CELLS_24H_UP
**557**	GSE5542_IFNA_VS_IFNA_AND_IFNG_TREATED_EPITHELIAL_CELLS_6H_DN
**558**	GSE5542_IFNG_VS_IFNA_AND_IFNG_TREATED_EPITHELIAL_CELLS_24H_DN
**559**	GSE5542_IFNG_VS_IFNA_TREATED_EPITHELIAL_CELLS_6H_DN
**560**	GSE5542_UNTREATED_VS_IFNA_AND_IFNG_TREATED_EPITHELIAL_CELLS_6H_UP
**561**	GSE5542_UNTREATED_VS_IFNG_TREATED_EPITHELIAL_CELLS_6H_UP
**562**	GSE5589_IL6_KO_VS_IL10_KO_LPS_STIM_MACROPHAGE_180MIN_UP
**563**	GSE5589_IL6_KO_VS_IL10_KO_LPS_STIM_MACROPHAGE_45MIN_DN
**564**	GSE5589_LPS_AND_IL10_VS_LPS_AND_IL6_STIM_IL10_KO_MACROPHAGE_45MIN_UP
**565**	GSE5589_LPS_VS_LPS_AND_IL6_STIM_IL10_KO_MACROPHAGE_45MIN_UP
**566**	GSE5589_WT_VS_IL10_KO_LPS_AND_IL10_STIM_MACROPHAGE_180MIN_UP
**567**	GSE5589_WT_VS_IL10_KO_LPS_AND_IL6_STIM_MACROPHAGE_45MIN_DN
**568**	GSE5589_WT_VS_IL6_KO_LPS_STIM_MACROPHAGE_180MIN_DN
**569**	GSE6090_UNSTIM_VS_DC_SIGN_STIM_DC_DN
**570**	GSE6092_UNSTIM_VS_IFNG_STIM_AND_B_BURGDORFERI_INF_ENDOTHELIAL_CELL_DN
**571**	GSE6259_33D1_POS_DC_VS_BCELL_UP
**572**	GSE6259_DEC205_POS_DC_VS_CD8_TCELL_DN
**573**	GSE6269_HEALTHY_VS_STAPH_PNEUMO_INF_PBMC_UP
**574**	GSE6566_STRONG_VS_WEAK_DC_STIMULATED_CD4_TCELL_DN

**575**	GSE6566_STRONG_VS_WEAK_DC_STIMULATED_CD4_TCELL_UP
**576**	GSE6674_CPG_VS_PL2_3_STIM_BCELL_UP
**577**	GSE6875_TCONV_VS_TREG_DN
**578**	GSE7460_CD8_TCELL_VS_CD4_TCELL_ACT_DN
**579**	GSE7460_CD8_TCELL_VS_TREG_ACT_UP
**580**	GSE7460_CTRL_VS_FOXP3_OVEREXPR_TCONV_UP
**581**	GSE7460_CTRL_VS_TGFB_TREATED_ACT_CD8_TCELL_UP
**582**	GSE7460_CTRL_VS_TGFB_TREATED_ACT_FOXP3_MUT_TCONV_UP
**583**	GSE7460_CTRL_VS_TGFB_TREATED_ACT_TCONV_UP
**584**	GSE7460_FOXP3_MUT_VS_HET_ACT_TCONV_UP
**585**	GSE7460_FOXP3_MUT_VS_WT_ACT_WITH_TGFB_TCONV_UP
**586**	GSE7460_TREG_VS_TCONV_ACT_WITH_TGFB_DN
**587**	GSE7568_CTRL_VS_3H_TGFB_TREATED_MACROPHAGES_WITH_IL4_AND_DEXAMETHASONE_UP
**588**	GSE7596_AKT_TRANSD_VS_CTRL_CD4_TCONV_WITH_TGFB_DN
**589**	GSE7764_IL15_TREATED_VS_CTRL_NK_CELL_24H_DN
**590**	GSE7831_CPG_VS_INFLUENZA_STIM_PDC_4H_DN
**591**	GSE7831_CPG_VS_INFLUENZA_STIM_PDC_4H_UP
**592**	GSE7831_UNSTIM_VS_INFLUENZA_STIM_PDC_1H_UP
**593**	GSE7852_LN_VS_THYMUS_TREG_DN
**594**	GSE7852_TREG_VS_TCONV_LN_DN
**595**	GSE8515_CTRL_VS_IL6_4H_STIM_MAC_DN
**596**	GSE8621_LPS_STIM_VS_LPS_PRIMED_AND_LPS_STIM_MACROPHAGE_UP
**597**	GSE8678_IL7R_LOW_VS_HIGH_EFF_CD8_TCELL_DN
**598**	GSE8685_IL15_ACT_IL2_STARVED_VS_IL21_ACT_IL2_STARVED_CD4_TCELL_UP
**599**	GSE8835_CD4_VS_CD8_TCELL_DN
**600**	GSE8921_UNSTIM_VS_TLR1_2_STIM_MONOCYTE_24H_DN
**601**	GSE9006_HEALTHY_VS_TYPE_1_DIABETES_PBMC_1MONTH_POST_DX_DN
**602**	GSE9006_HEALTHY_VS_TYPE_1_DIABETES_PBMC_4MONTH_POST_DX_DN
**603**	GSE9006_HEALTHY_VS_TYPE_1_DIABETES_PBMC_AT_DX_UP
**604**	GSE9006_TYPE_1_DIABETES_AT_DX_VS_1MONTH_POST_DX_PBMC_DN
**605**	GSE9037_CTRL_VS_LPS_4H_STIM_BMDM_UP
**606**	GSE9037_WT_VS_IRAK4_KO_BMDM_DN
**607**	GSE9509_10MIN_VS_30MIN_LPS_AND_IL10_STIM_IL10_KO_MACROPHAGE_UP
**608**	GSE9509_LPS_VS_LPS_AND_IL10_STIM_IL10_KO_MACROPHAGE_20MIN_UP
**609**	GSE9650_EXHAUSTED_VS_MEMORY_CD8_TCELL_UP
**610**	GSE9946_IMMATURE_VS_LISTERIA_INF_MATURE_DC_DN
**611**	GSE9946_IMMATURE_VS_LISTERIA_INF_MATURE_DC_UP
**612**	GSE9946_IMMATURE_VS_PROSTAGLANDINE2_TREATED_MATURE_DC_DN
**613**	GSE9946_IMMATURE_VS_PROSTAGLANDINE2_TREATED_MATURE_DC_UP
**614**	GSE9946_LISTERIA_INF_MATURE_VS_PROSTAGLANDINE2_TREATED_MATURE_DC_UP
**615**	GSE9960_HEALTHY_VS_GRAM_NEG_AND_POS_SEPSIS_PBMC_UP
**616**	GSE9988_LPS_VS_LOW_LPS_MONOCYTE_DN

**617**	KAECH_DAY15_EFF_VS_MEMORY_CD8_TCELL_UP
**618**	FOURATI_BLOOD_TWINRIX_AGE_25_83YO_RESPONDERS_VS_POOR_RESPONDERS_0DY_DN
**619**	FOURATI_BLOOD_TWINRIX_AGE_25_83YO_RESPONDERS_VS_POOR_RESPONDERS_0DY_UP
**620**	HOEK_NEUTROPHIL_2011_2012_TIV_ADULT_1DY_DN
**621**	HOWARD_NEUTROPHIL_INACT_MONOV_INFLUENZA_A_INDONESIA_05_2005_H5N1_AGE_18_49YO_1DY_DN
**622**	NAKAYA_B_CELL_FLUARIX_FLUVIRIN_AGE_18_50YO_7DY_DN
**623**	NAKAYA_MYELOID_DENDRITIC_CELL_FLUMIST_AGE_18_50YO_7DY_UP
**624**	NAKAYA_PLASMACYTOID_DENDRITIC_CELL_FLUMIST_AGE_18_50YO_7DY_DN

Table S9

Significantly enriched pathways from C8

Number	Enriched pathway
**1**	BUSSLINGER_DUODENAL_BCHE_CELLS
**2**	BUSSLINGER_DUODENAL_TUFT_CELLS
**3**	DESCARTES_FETAL_ADRENAL_CSH1_CSH2_POSITIVE_CELLS
**4**	DESCARTES_FETAL_CEREBRUM_MEGAKARYOCYTES
**5**	DESCARTES_FETAL_CEREBRUM_OLIGODENDROCYTES
**6**	DESCARTES_FETAL_EYE_RETINAL_PIGMENT_CELLS
**7**	DESCARTES_FETAL_INTESTINE_ENS_NEURONS
**8**	DESCARTES_FETAL_INTESTINE_MESOTHELIAL_CELLS
**9**	DESCARTES_FETAL_INTESTINE_STROMAL_CELLS
**10**	DESCARTES_FETAL_KIDNEY_MESANGIAL_CELLS
**11**	DESCARTES_FETAL_MUSCLE_SCHWANN_CELLS
**12**	DESCARTES_FETAL_PANCREAS_MESOTHELIAL_CELLS
**13**	DESCARTES_FETAL_PLACENTA_TROPHOBLAST_GIANT_CELLS
**14**	DESCARTES_FETAL_SPLEEN_ERYTHROBLASTS
**15**	DESCARTES_FETAL_SPLEEN_VASCULAR_ENDOTHELIAL_CELLS
**16**	DESCARTES_FETAL_THYMUS_THYMIC_EPITHELIAL_CELLS
**17**	DESCARTES_MAIN_FETAL_CCL19_CCL21_POSITIVE_CELLS
**18**	DESCARTES_MAIN_FETAL_EPICARDIAL_FAT_CELLS
**19**	DESCARTES_MAIN_FETAL_THYMIC_EPITHELIAL_CELLS
**20**	FAN_EMBRYONIC_CTX_BIG_GROUPS_CAJAL_RETZIUS

**21**	GAO_ESOPHAGUS_25W_C1_CILIATED_EPITHELIAL_CELLS
**22**	GAO_SMALL_INTESTINE_24W_C9_ENTEROENDOCRINE_CELL
**23**	GAUTAM_EYE_IRIS_CILIARY_BODY_CYTOTOXIC_T_CELLS
**24**	HAY_BONE_MARROW_CD34_POS_PRE_B
**25**	HAY_BONE_MARROW_EARLY_ERYTHROBLAST
**26**	HE_LIM_SUN_FETAL_LUNG_C0_EARLY_MESOTHELIAL_CELL
**27**	HE_LIM_SUN_FETAL_LUNG_C0_LATE_MESOTHELIAL_CELL
**28**	HE_LIM_SUN_FETAL_LUNG_C0_MID_MESOTHELIAL_CELL
**29**	HE_LIM_SUN_FETAL_LUNG_C1_MUC5AC_POS_ASCL1_POS_PROGENITOR_CELL
**30**	HE_LIM_SUN_FETAL_LUNG_C1_SMG_CELL
**31**	HE_LIM_SUN_FETAL_LUNG_C4_ILC2_CELL
**32**	HE_LIM_SUN_FETAL_LUNG_C7_SST_POS_NEURON_CELL
**33**	HE_LIM_SUN_FETAL_LUNG_C7_TM4SF4_POS_CHODL_POS_NEURON_CELL
**34**	HU_FETAL_RETINA_PHOTORECEPTOR
**35**	MANNO_MIDBRAIN_NEUROTYPES_HDA
**36**	MANNO_MIDBRAIN_NEUROTYPES_HGABA
**37**	MANNO_MIDBRAIN_NEUROTYPES_HNBGABA
**38**	MANNO_MIDBRAIN_NEUROTYPES_HNBM
**39**	MANNO_MIDBRAIN_NEUROTYPES_HOPC
**40**	MANNO_MIDBRAIN_NEUROTYPES_HRGL2B
**41**	MURARO_PANCREAS_PANCREATIC_POLYPEPTIDE_CELL
**42**	TRAVAGLINI_LUNG_CILIATED_CELL
**43**	TRAVAGLINI_LUNG_PROXIMAL_BASAL_CELL

Table S10

Top 20 frequent genes in differentially enriched pathways between (Tie2+/CD14+) group and (Tie2-/CD14+) controls

Number	GENE	SET	FREQ
**1**	CITED2	C3	7
**2**	RARB	C3	6
**3**	ARFGEF2	C3	5
**4**	CADM1	C3	5
**5**	HOXB5	C3	5
**6**	HOXB8	C3	5
**7**	MAP1A	C3	5
**8**	MEIS1	C3	5
**9**	SEMA6D	C3	5
**10**	COL14A1	C4	8
**11**	DDX11	C4	8

**12**	PDE4D	C4	8
**13**	CDH5	C4	8
**14**	GEMIN7	C4	7
**15**	MAGI1	C4	7
**16**	MPZL2	C4	7
**17**	PIGL	C4	7
**18**	CCR7	C5	6
**19**	PDGFRA	C5	6
**20**	CPE	C5	6
